# Facile N-functionalization and strong magnetic communication in a diuranium(v) bis-nitride complex†
†Electronic supplementary information (ESI) available: Supplementary NMR, IR, UV-Vis-NIR and magnetic and crystallographic data. CCDC 1883481–1883486. For ESI and crystallographic data in CIF or other electronic format see DOI: 10.1039/c8sc05721d


**DOI:** 10.1039/c8sc05721d

**Published:** 2019-02-18

**Authors:** Luciano Barluzzi, Lucile Chatelain, Farzaneh Fadaei-Tirani, Ivica Zivkovic, Marinella Mazzanti

**Affiliations:** a Institut des Sciences et Ingénierie Chimiques , Ecole Polytechnique Fédérale de Lausanne (EPFL) , CH-1015 Lausanne , Switzerland . Email: marinella.mazzanti@epfl.ch; b Laboratory for Quantum Magnetism , Institute of Physics , Ecole Polytechnique Fédérale de Lausanne (EPFL) , CH-1015 Lausanne , Switzerland

## Abstract

Uranium nitride complexes are of high interest because of their ability to effect dinitrogen reduction and functionalization and to promote magnetic communication, but studies of their properties and reactivity remain rare. Here we have prepared in 73% yield the diuranium(v) bis-nitride complex [K_2_{[U(OSi(O^*t*^Bu)_3_)_3_]_2_(μ-N)_2_}], **4**, from the thermal decomposition of the nitride-, azide-bridged diuranium(iv) complex [K_2_{[U(OSi(O^*t*^Bu)_3_)_3_]_2_(μ-N)(μ-N_3_)}], **3.** The bis-nitride **4** reacts in ambient conditions with 1 equiv. of CS_2_ and 1 equiv. of CO_2_ resulting in N–C bond formation to afford the diuranium(v) complexes [K_2_{[U(OSi(O^*t*^Bu)_3_)_3_]_2_(μ-N)(μ-S)(μ-NCS)}], **5** and [K_2_{[U(OSi(O^*t*^Bu)_3_)_3_]_2_(μ-N)(μ-O)(μ-NCO)}], **6**, respectively. Both nitrides in **4** react with CO resulting in oxidative addition of CO to one nitride and CO cleavage by the second nitride to afford the diuranium(iv) complex [K_2_{[U(OSi(O^*t*^Bu)_3_)_3_]_2_(μ-CN)(μ-O)(μ-NCO)}], **7**. Complex **4** also effects the remarkable oxidative cleavage of H_2_ in mild conditions to afford the bis-imido bridged diuranium(iv) complex [K_2_{[U(OSi(O^*t*^Bu)_3_)_3_]_2_(μ-NH)_2_}], **8** that can be further protonated to afford ammonia in 73% yield. Complex **8** provides a good model for hydrogen cleavage by metal nitrides in the Haber–Bosch process. The measured magnetic data show an unusually strong antiferromagnetic coupling between uranium(v) ions in the complexes **4** and **6** with Neel temperatures of 77 K and 60 K respectively, demonstrating that nitrides are attractives linkers for promoting magnetic communication in uranium complexes.

## Abstract 2

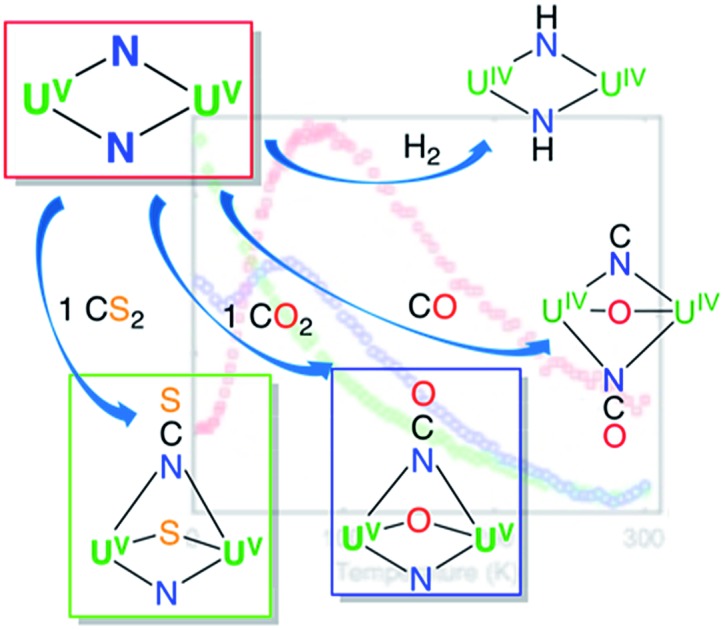
A diuranium(v) bis-nitride complex supported by siloxide ligands displays remarkable reactivity in ambient conditions with small molecules such as CS_2_, CO_2_, CO and H_2_ resulting in N–C and N–H bond formation. The nitride linker also leads to an unusually strong antiferromagnetic coupling between uranium(v) ions.

## Introduction

Metal nitrides are of interest because of their role as intermediates in biological and industrial dinitrogen fixation,^1^ and of their potential application in nitrogen transfer chemistry and in small molecule activation.1c,^2^ N–H and N–C bond formation reactions are particularly important in the context of industrial ammonia production and in the synthesis of value-added organic compounds respectively.^3^ However examples of nitride functionalization by CO,2d,j,^4^ CO_2_, CS_2_ 2f,i,^5^ or H_2_ 2a–e,^6^ remain surprisingly rare.

The high activity of uranium nitride materials (UN) as catalysts for the transformation of N_2_ and H_2_ into ammonia in the Haber–Bosch process and their potential application as nuclear fuels has attracted large interest in the synthesis and reactivity of molecular uranium nitrides.^7^

Uranium nitrides are also of interest in the fundamental study of U–N multiple bonding^8^ and magnetic communication between uranium centers.^9^ In recent years several mononuclear, dinuclear and polynuclear molecular mono(nitride) compounds of uranium in oxidation states ranging from +3 to +6 have been prepared and characterized.4c,d,7e,9a,^10^ Highly reactive transient terminal uranium nitrides that promote C–H activation of the supporting ligand were also identified.^11^ The few reported reactivity studies of isolated molecular uranium nitrides demonstrated a surprisingly high activity of terminal and bridging nitrides towards the activation of unreactive small molecules resulting in the formation of new N–H and N–C bonds.4b–d,5a,g,^6^,10h Notably, the reactions of the diuranium(iv) nitride bridged [Cs{[U(OSi(O^*t*^Bu)_3_)_3_]_2_(μ-N)}] complex with CO_2_, CS_2_, CO and H_2_ are all ligand centered and involve electrophilic addition to the highly nucleophilic bridging nitride. In contrast, the reactions of a terminal U(v) nitride, [U(Tren^TIPS^)(N)][K(B15C5)_2_], with CS_2_ and CO show a nucleophilic reactivity of the nitride associated to redox changes at the metal center. Moreover, electrophilic reactivity towards cyanide was also reported for a nitride bridged diuranium(v).10*h* However, examples of functionalization of bis(μ-nitride) complexes are lacking in uranium chemistry and are extremely rare for d-metals^12^ in spite of the fact that such species have been reported as the product of bimetallic dinitrogen reduction in reducing conditions.12b,^13^

Here, we report a reproducible synthetic route to the diuranium(v) bis-nitride complex [K_2_{[U(OSi(O^*t*^Bu)_3_)_3_]_2_(μ-N)_2_}]. This rare example of nitride bridged U(v) complex was previously crystallographically characterized,10*i* but could not be prepared analytically clean in reasonable amounts preventing further characterization and reactivity studies. We now report the reactivity of the diuranium(v) bis-nitride with small molecules (CO, CO_2_, CS_2_, H_2_) that has resulted in N–C and N–H bond formation. We also report the isolation and characterization of the products obtained from these reactions where the bridging nitride group acts as a strong nucleophile, yielding cyanate, thiocyanate, isocyanide, sulphide and imide groups bridging uranium(iv) or uranium(v) cations.

The greater radial extension of 5f orbitals compared to 4f orbitals may provide enough orbital overlap with ligand orbitals to implement magnetic communication between two metal centers through multiply bound bridging atoms.10b,^14^ However, clear cut examples of magnetic communications in uranium chemistry remain limited and magnetic exchange is usually quite weak in chalcogenide or nitride bridged diuranium complexes.9b,^15^ In contrast, unusually strong antiferromagnetic exchange interactions, have been reported for a bis-oxide bridged diuranium(v) complex with a diamond shaped U(O)_2_U core.^16^ The development of a reproducible synthetic route to the U(v)/U(v) bis-nitride complex has now rendered possible to investigate its magnetic properties and to compare them with those of other nitride bridged complex obtained from the reaction of the bis-nitride with small molecules.

## Results and discussion

### Synthesis of a dinuclear U(v) bis-nitride complex

**Scheme 1:**
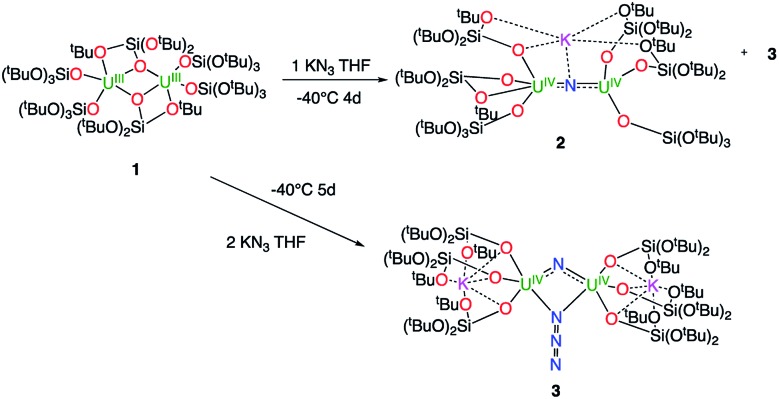
Reactivity of complex [U(OSi(O^*t*^Bu)_3_)_3_]_2_, **1** with 1 and 2 equivalents of KN_3_.

**Fig. 1:**
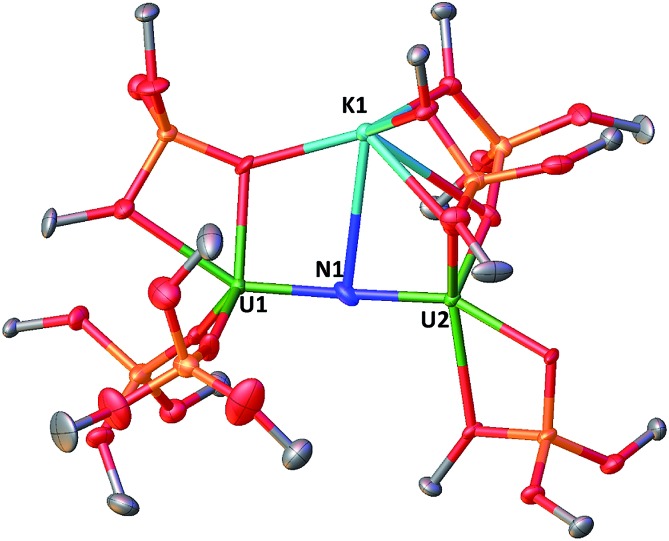
Molecular structure of [K{[U(OSi(O^*t*^Bu)_3_)_3_]_2_(μ-N)}] in crystal of **2** shown with 50% probability thermal ellipsoids. Hydrogen atoms and the methyl groups of the *tert*-butyl moieties have been omitted for clarity.

^1^H NMR studies show that the reaction of the dinuclear uranium(iii) complex [U(OSi(O^*t*^Bu)_3_)_3_]_2_, **1** 17 with one equivalent of KN_3_ in THF at –40 °C for 4 days leads to the formation of the nitride-bridged diuranium(iv) complex [K{[U(OSi(O^*t*^Bu)_3_)_3_]_2_(μ-N)}], **2** and the nitride-, azide-bridged diuranium(iv) complex [K_2_{[U(OSi(O^*t*^Bu)_3_)_3_]_2_(μ-N)(μ-N_3_)}], **3** together with unreacted starting material (Scheme 1). Successive recrystallizations of the reaction mixture in THF at –40 °C allows the isolation of pure crystalline [K{[U(OSi(O^*t*^Bu)_3_)_3_]_2_(μ-N)}], **2** in 21% yield. The solid-state structure of **2** is presented in Fig. 1. It consists of a heterometallic complex (U_2_K) in which two U(iv) cations are held together by a nitrido ligand (N^3–^) arranged in an almost linear fashion (UN[combining circumflex]U = 170.1(5)°). The values of the U–N bond distances (2.089(9) Å and 2.076(9) Å) are very close to those found in the previously reported diuranium(iv) nitride [Cs{[U(OSi(O^*t*^Bu)_3_)_3_]_2_(μ-N)}] (2.058(5) and 2.079(5) Å) that presents a similar linear U–N–U arrangement (170.2(3)°).10*i* The K^+^ cation is held at the apical position of the nitrido ligand by 5 donor atoms from three siloxide ligands.

When two equivalents of KN_3_ are reacted with [U(OSi(O^*t*^Bu)_3_)_3_]_2_, **1**, only the formation of [K_2_{[U(OSi(O^*t*^Bu)_3_)_3_]_2_(μ-N)(μ-N_3_)}], **3** could be observed. The reaction probably proceeds through the reaction of **1** with a first equivalent of azide to afford the diuranium(iv) nitride **2** that reacts faster than unreacted **1** with a second equivalent of KN_3_ to afford complex **3**. Crystals of complex **3** suitable for X-ray diffraction were isolated from a cold (–40 °C) toluene solution in 72% yield.

**Fig. 2:**
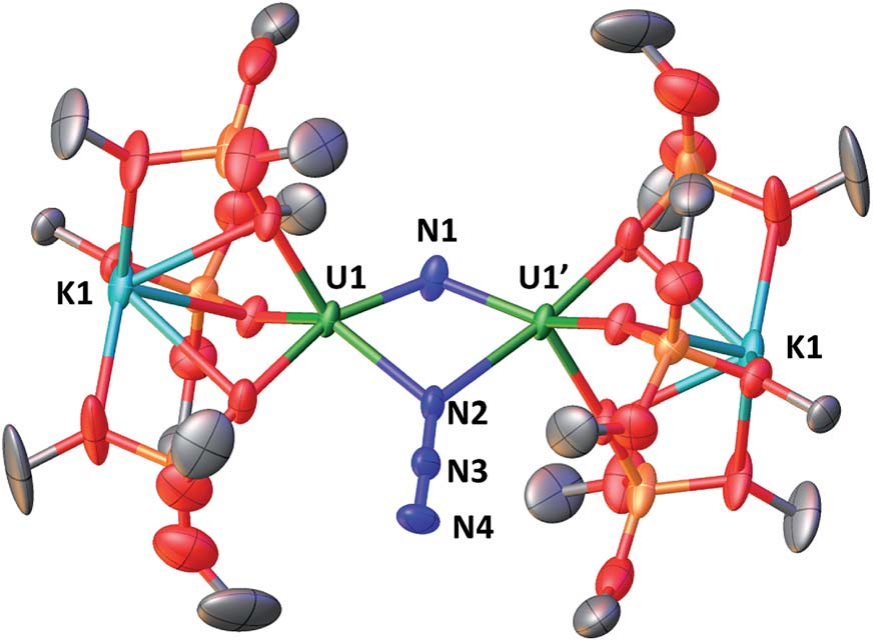
Molecular structure of [K_2_{[U(OSi(O^*t*^Bu)_3_)_3_]_2_(μ-N)(μ-N_3_)}] in crystal of **3** shown with 50% probability thermal ellipsoids. Hydrogen atoms, the methyl groups of the *tert*-butyl moieties and lattice solvent have been omitted for clarity.

The solid-state structure of **3** is presented in Fig. 2. It consists of a heterometallic complex (U_2_K_2_) in which two U(iv) cations are held together by a nitrido and an azido (N_3_^–^) ligand arranged in a diamond core fashion (U–N_nitride_–U = 124.6(6)° and U–N_azide_–U = 91.4(5)°). The U–nitride bond distances (2.018(12) Å, 2.086(11) Å) in **3** compare well with the U–N distances found in the U(v)/U(v) bis-nitride complex **4** (2.101(6) and 2.022(5) Å)10*i* and in the anionic U(v)/(Uiv) mixed-valent bis-nitride complex, [{K(dme)(calix[4]tetrapyrrole)U}_2_(μ-NK)_2_][K(dme)_4_] (2.076(6) and 2.099(5) Å).10*d* The value of the U–N_nitride_–U angle is larger compared to that found in **4** (106.1(2)°). The two uranium centers in **3** have a distorted square pyramidal geometry and are crystallographically equivalent, being related by an inversion center found at the middle of the UN1N2U′ core. The U–azide bond distances (U1–N2 = 2.492(11) Å, U1′–N2 = 2.578(12) Å) lie in the range of the values reported for 1,1-end-on azides in U(iv)10*f* and U(vi)^18^ complexes (2.453(7)–2.565(2) Å).

Complex **3** is soluble but not stable in toluene at room temperature and slowly decomposes affording a complex mixture of compounds (Fig. S6†), in which, the presence of the diuranium(v) bis-nitride complex [K_2_{[U(OSi(O^*t*^Bu)_3_)_3_]_2_(μ-N)_2_}], **4**,10*i* was identified by ^1^H NMR spectroscopy.

**Scheme 2:**
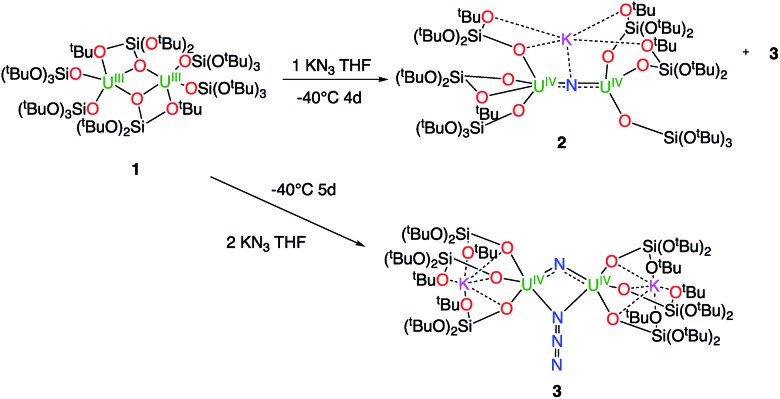
Synthesis of complex [K_2_{[U(OSi(O^*t*^Bu)_3_)_3_]_2_(μ-N)_2_}], **4**.

The decomposition is faster at higher temperatures and, when a solution of complex **3** in toluene is stirred for 24 h at 70 °C, the bis-nitride complex **4** is obtained as major product in 73% yield (Scheme 2). Complex **4** is soluble in THF and toluene and is stable both in solid state and in solution at room temperature and at 70 °C. The solid state structure of **4** was previously reported10*i* by our group. Single crystals of complex **4** were previously10*i* isolated from the reaction of the U(iii) complex [K(18c6)][U(OSi(O^*t*^Bu)_3_)_4_] with cesium azide in THF at –40 °C. However, all attempts to prepare complex **4** pure in significant amounts failed, preventing further characterization and reactivity studies for this complex. Thus, the reaction shown in Scheme 2 provides a convenient and reproducible synthetic route for the complex **4**.

Complex **4** is only the second example of a bis-nitride complex of uranium and shows a diamond shaped arrangement of the two nitride ligands10*i* as found in the anionic U(v)/U(iv) mixed-valent complex, [{K(dme)(calix[4]tetrapyrrole)U}_2_(μ-NK)_2_]-[K(dme)_4_]10*d* obtained from dinitrogen cleavage by a U(iii)/[K(naphth)]/DME system.

Complex **4** provides an attractive system for the study of the reactivity of bis-nitride species that may be formed from dinitrogen reduction.4c,10d,13a Thus, its ability to produce NH_3_ or C–N bonds, highly desirable feature for reduced dinitrogen compounds, was investigated.

**Scheme 3:**
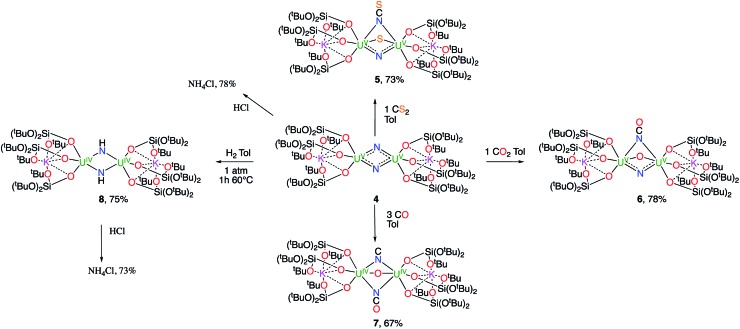
Reactivity of complex **4** towards small molecules.

The reactivity of complex **4** towards small molecules is summarized in Scheme 3.

### Reactivity of [K_2_{[U(OSi(O^*t*^Bu)_3_)_3_]_2_(μ-N)_2_}] towards small molecules

**Fig. 3:**
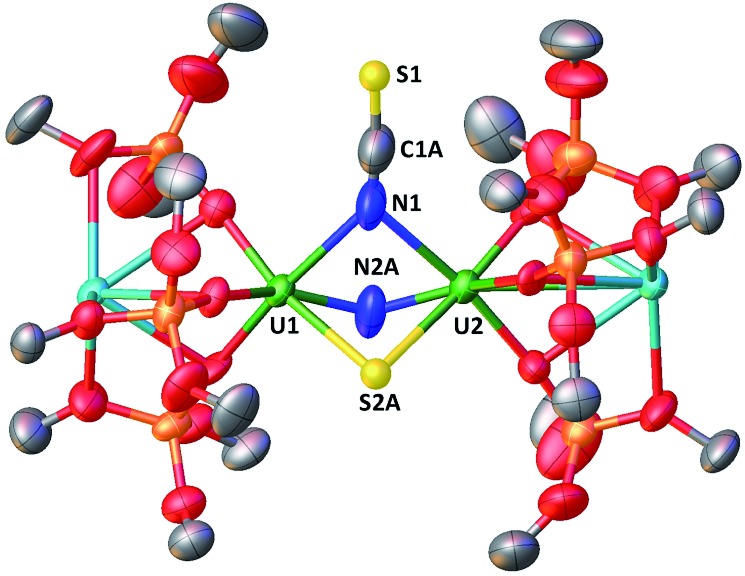
Molecular structure of [K_2_{[U(OSi(O^*t*^Bu)_3_)_3_]_2_(μ-N)(μ-S)(μ-NCS)}]in crystal of **5**·tol shown with 50% probability thermal ellipsoids. Hydrogen atoms, the methyl groups of the *tert*-butyl moieties and lattice solvent have been omitted for clarity.

When one equivalent of CS_2_ is added to a stirring solution of complex **4** in toluene, the colour changes from dark brown to dark orange. The ^1^H NMR spectrum of the reaction mixture over time shows that the reaction of **4** with CS_2_ is complete after a few hours to afford a new species which is stable over time in toluene. From a cold hexane solution, single crystals of the diuranium(v) complex [K_2_{[U(OSi(O^*t*^Bu)_3_)_3_]_2_(μ-N)(μ-S)(μ-NCS)}], **5** were isolated. The solid-state structure of **5** is presented in Fig. 3.

It shows a heterometallic complex (U_2_K_2_) in which the two U(v) cations are held together by three bridging ligands, namely a nitrido, a sulphido and a thiocyanato ligand, and are in a pseudo-octahedral geometry. The other coordination positions are occupied by the siloxide ligands, which provide a O_6_ binding pocket for the coordination of K^+^.

Complex **5** is the first example of a uranium compound with a κN:κN bridging thiocyanato. The mean value of the U–N_NCS_ distances (2.59(3) Å) is longer than the one found in the only reported U(v) thiocyanato complex where the thiocyanate is terminally bound (2.316 Å).^19^ A similar difference is observed between a κ^1^N:κ^1^S thiocyanato bridged diuranium(iv) complex (2.510(15) Å)4*c* and terminal5a,^20^ U(iv) thiocyanato complexes (2.428(4)–2.377(4) Å).

The study of U–S bonds has attracted significant current interest, but most examples of molecular uranium sulphides are sulphide bridged diuranium(iv) complexes with a fewer examples of terminal U(iv) sulphides.5a,15g,21a–l Molecular sulphides containing uranium in higher oxidation state are rare^22^ and only one example of a molecular terminal U(v) sulphide has been reported recently.^23^

Thus, the nucleophilic addition of nitride to carbon disulfide provides access to the first example of a bridging U(v) sulphide. The reaction of **4** with CS_2_ is similar to that reported for the mononitride complex diuranium(iv) [Cs{[U(OSi(O^*t*^Bu)_3_)_3_]_2_(μ-N)}] that reacts with CS_2_ to yield the trithiocarbonate Cs{(μ-NCS)(μ-CS_3_)[U(OSi(O^*t*^Bu)_3_)_3_]_2_}.5*g* A different reactivity was reported for the terminal U(v) nitride [U(Tren^TIPS^)(N)][K(B15C5)_2_] that, upon reaction with CS_2_, forms unstable intermediates that undergo disproportionation reactivity affording a trithiocarbonate complex of U(iv) and potassium thiocyanate.5*a*

The mean value of the U–S distances of 2.67(1) Å is significantly longer than that found in the only reported uranium(v) sulphide complex [K(2.2.2-cryptand)][US{OSi(O^*t*^Bu)_3_}_4_]^23^ where the sulphide is terminally bound (2.396(5) Å), but is close to the value reported ((2.66(2) Å) for the terminal U(v) persulfide complex [(η^2^-S_2_){U((SiMe_2_NPh)_3_-tacn)}]^24^ and for U(v) thiolate complexes (2.7230(13) Å).^25^

The ^13^C NMR spectrum in d_8_-toluene of ^13^C-**5** (obtained from the reaction of complex **4** with ^13^CS_2_) shows a resonance at *∂* = 132.06 ppm assigned to the N^13^CS ligand (Fig. S13†). The IR spectra of complex **5** and ^13^C-**5** show a band at 1977 and 1931 cm^–1^ respectively, which are attributed to the *ν*(CN) stretching (Fig. S48 and S49†). The low frequency of the IR band is in agreement with the presence of an N-bound thiocyanate.^26^

Addition of excess ^13^CS_2_ (10 equiv.) to **4** results in the formation of **5** followed by the slow, partial conversion of **5** into unidentified products. The ^13^C NMR spectrum in d_6_-dmso of the reaction mixture measured after 24 hours shows the presence of increased amounts of the extruded NCS^–^ anion compared to the stoichiometric reaction of **4** with ^13^CS_2_. This suggests that the second nitride also reacts slowly with CS_2_.

**Fig. 4:**
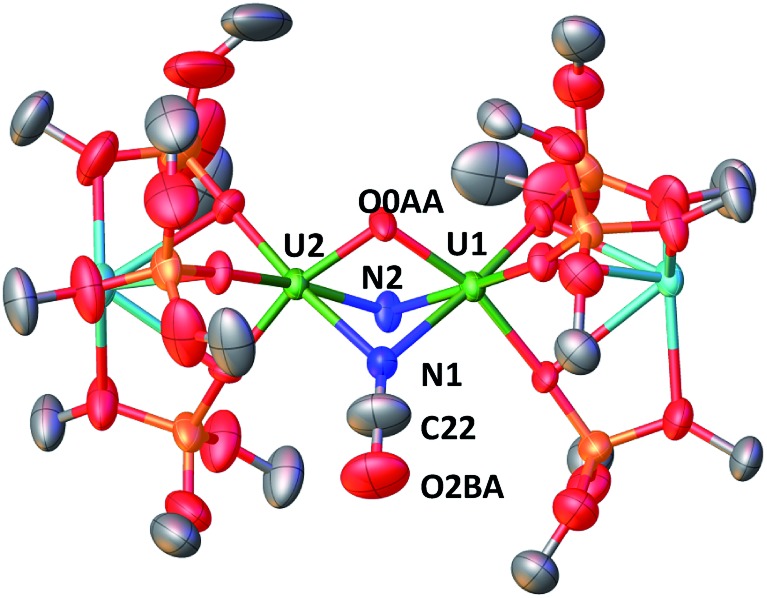
Molecular structure of [K_2_{[U(OSi(O^*t*^Bu)_3_)_3_]_2_(μ-N)(μ-O)(μ-NCO)}] in crystal of **6** shown with 50% probability thermal ellipsoids. Hydrogen atoms and the methyl groups of the *tert*-butyl moieties have been omitted for clarity.

These results show the high nucleophilicity of the nitride ligands in **4** leading to C–S cleavage and N–C bond formation and prompted us to investigate the reactivity of complex **4** with CO_2_. The addition of one equivalent of CO_2_ to a toluene solution of complex **4** immediately leads to a colour change from dark brown to ochre yellow. The ^1^H NMR spectrum of the reaction mixture shows immediate reaction of **4** to afford a new species which is stable over time in toluene. Cooling down the solution to –40 °C affords crystals of the oxo–nitride–cyanate bridged diuranium(v) complex [K_2_{[U(OSi(O^*t*^Bu)_3_)_3_]_2_(μ-N)(μ-O)(μ-NCO)}], **6**, in 78% yield. The ^13^C labelled analogue ^13^C-**6** was also prepared with a similar procedure from ^13^CO_2_. The solid-state structure of **6** is presented in Fig. 4. The structure of **6** shows the presence of a heterodimetallic complex (U_2_K_2_) in which the two U(v) cations are held together by three bridging ligands, namely a nitrido, an oxo and a cyanato ligand and are in a pseudo octahedral geometry. The other coordination positions are occupied by the siloxide ligands, which also provide a O_6_ coordination pocket suitable for the coordination of K^+^.

The average U–N_nitride_ (2.107(41) Å) and U–O_oxo_ (2.103(27) Å) distances are in the range of those previously reported for nitride-bridged (2.0470(3)–2.101(6) Å)4c,10h,i and oxo-bridged diuranium(v) complexes (2.0354(12)–2.1815(11) Å)9b,15e,g,^16^ diuranium complexes. The average U–N_cyanate_ distance of 2.495(12) Å is slightly shorter than the one found in the previously reported diuranium(iv) [Cs{[U(OSi(O^*t*^Bu)_3_)_3_]_2_(μ-O)(μ-NCO)}] (2.559(9) Å) complex.4*c*

The ^13^C NMR spectrum in d_8_-toluene of ^13^C-**6** shows a resonance at *∂* = 143.06 ppm attributed to the N^13^CO ligand (Fig. S20†).

The IR spectra of **6** and ^13^C-**6** show peaks at 2192 and 2133 cm^–1^ respectively (Fig. S50 and S51†), that are in agreement with the values found for previously reported cyanato ligands in uranium complexes.4c,d,^20^,^27^,^28^

Upon addition of 2 equivalents of CO_2_ to a degassed toluene solution of complex **4** the ^1^H NMR spectrum in d_8_-toluene reveals the presence of complex **6**, [U(OSi(O^*t*^Bu)_3_)_4_], and other unidentified products. Upon addition of a larger excess of ^13^CO_2_ (10 equiv.) to a degassed toluene solution of complex **4** the ^1^H NMR spectrum in d_8_-toluene reveals the presence of multiple species that were not identified (Fig. S23†). When the volatiles are removed under vacuum and the resulting solid residue is dissolved in d_6_-dmso, the ^13^C NMR of the reaction mixture shows only the presence of KN^13^CO which is extruded from the complex (Fig. S24†). Thus, the addition of an excess of CO_2_ to complex **4** does not lead to a different type of reactivity but, most probably, only to the reaction of the second nitride with CO_2_ to afford cyanate followed by ligand scrambling.

The highly nucleophilic bridging nitride in the diuranium(v) complex effects the complete cleavage of the C=O double bond of the CO_2_ molecule to afford the cyanate and oxide groups. A carbamate is the probable intermediate that could not be identified. A similar reactivity was also reported for the terminal U(v) nitride [U(Tren^TIPS^)(N)][K(B15C5)_2_].5*a* The reactivity of the [U^V^(μ-N)_2_U^V^] core differs from what reported previously in our group for the diuranium(iv) mononitride [U^IV^(μ-N)U^IV^] that reacts with 1 equivalent of CO_2_ to afford both a dicarbamate ligand bridging the two U(iv) cations and an oxo–cyanate bridged diuranium(iv) complex.5*g* However, in both cases the reactivity does not involve redox changes at the metal centers but is only promoted by the high nucleophilic character of the nitride.

The high nucleophilicity of the bridging nitride prompted us to investigate if C–N bonds could be formed also with other C1 sources, such as CO.

When CO (2–3 equivalents) is added to a degassed toluene solution of complex **4,** the colour immediately changes from dark brown to light green. Cooling the solution down to –40 °C affords the diuranium(iv) complex [K_2_{[U(OSi(O^*t*^Bu)_3_)_3_]_2_(μ-CN)(μ-O)(μ-NCO)}], **7**, as green crystals.

**Fig. 5:**
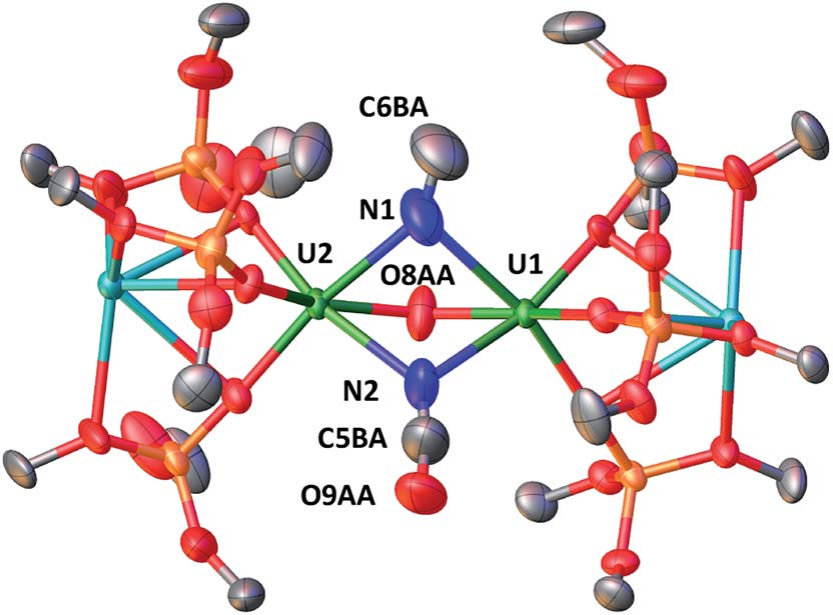
Molecular structure of [K_2_{[U(OSi(O^*t*^Bu)_3_)_3_]_2_(μ-CN)(μ-O)(μ-NCO)}] in crystal of **7**·tol shown with 50% probability thermal ellipsoids. Hydrogen atoms, the methyl groups of the *tert*-butyl moieties, crystallographically disordered atoms and lattice solvent have been omitted for clarity. Selected bond lengths (Å).

The solid-state structure of **7**, presented in Fig. 5, shows an heterodimetallic complex (U_2_K_2_) in which the two U cations are held together by three bridging ligands, namely an oxo, a cyanato and a isocyanido (the cyanato and isocyanido ligands are crystallographically disordered over two positions). The other three coordination positions are occupied by the siloxide ligands, which also provide a O_6_ coordination pocket suitable for the coordination of K^+^. The U–O_oxo_ bond distance (2.145(19) Å) is longer than the one found in complex **6** and is in the range of the reported values for oxo-bridged diuranium(iv) complexes (2.068(2)–2.176(2) Å).4b,5g,9b,^29^ An end-on bridging isocyanido ligand is unprecedented in uranium chemistry, but a side-on bridging isocyanide was previously obtained from the reaction of the diuranium(iv) complex [Cs{[U(OSi(O^*t*^Bu)_3_)_3_]_2_(μ-N)}] with CO.4*b* The preferential N-bonding mode of cyanide anion to U(iv) complexes has been reported previously and corroborated by DFT calculations^30^ showing a better energy matching between the metal and ligand orbitals for the U(iv)–N compared to the U(iv)–C bonding mode.

The ^13^C NMR spectrum of isolated crystals of ^13^C-**7** in toluene shows two peaks at 214.19 and at –158.48 ppm (Fig. S30†) attributed to the uranium-bound cyanato and the isocyanido ligands.

Thus, upon addition of CO to **4** both nitride ligands react with CO, but with different outcome. One nitride cleaves completely the CO triple bond yielding a cyanide and an oxo group, while the second nitride undergoes reductive carbonylation to afford a cyanate bridging ligand with concomitant two electron reduction and formation of a diuranium(iv) complex. No conclusions can be drawn about the order of reactivity, since no intermediate can be isolated. In fact, when one equivalent of CO is added to a toluene solution of complex **4**, only complex **7** and unreacted complex **4** can be identified in the reaction mixture by ^1^H NMR spectroscopy (Fig. S25†). This suggests that the putative intermediate reacts with CO much faster than the starting complex **4.** The observed reactivity suggests that the formation of a U(iii) bridging cyanate is not accessible from bridging U(v) nitrides. This differs from what reported for a terminal U(v) nitride where the reaction with CO leads only to the reductive carbonylation and formation of a U(iii) cyanate complex5*a* rather than to CO cleavage. Such difference is likely to arise from the cooperative binding of CO by the two uranium centers and the high nitride nucleophilic character which remarkably leads to cleavage of the CO triple bond.

### Nitride hydrogenation and protonation

Since nitrides are likely intermediates in the catalytic dinitrogen reduction to ammonia, hydrogen cleavage by metal nitrides is believed to be an important step in the Haber–Bosch process.1a,b In spite of this only a handful of nitrides, bridging or terminal, that can effect H_2_ cleavage have been reported2a–e none of which were bis(μ-nitrido).

Very recently we reported that the diuranium(iv) nitride bridged complex [Cs{[U(OSi(O^*t*^Bu)_3_)_3_]_2_(μ-N)}] promotes the reversible heterolytic activation of H_2_ to afford the hydride, imide bridged diuranium(iv) complex [Cs{U(OSi(O^*t*^Bu)_3_)_3_}_2_(μ-H)(μ-NH)].^6^

A strikingly different reactivity is observed for complex **4** when is exposed to 1 atm of H_2_. The reaction proceeds slowly at room temperature, but, after 12 h, the analysis of the reaction mixture by ^1^H NMR spectroscopy reveals the complete consumption of the starting material and the clean formation of a new species.

**Fig. 6:**
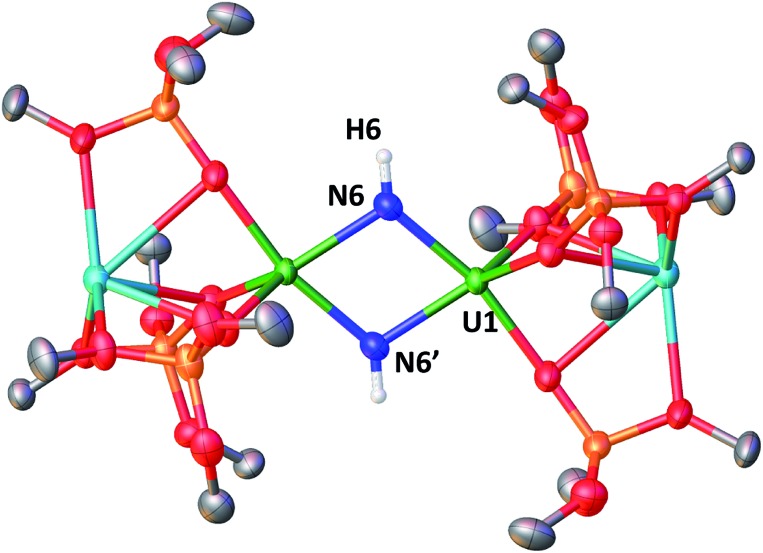
Molecular structure of [K_2_{[U(OSi(O^*t*^Bu)_3_)_3_]_2_(μ-NH)_2_}] in crystal of **8**·tol shown with 50% probability thermal ellipsoids. Hydrogen atoms, the methyl groups of the *tert*-butyl moieties and lattice solvent have been omitted for clarity.

When the reaction mixture is heated up to 60 °C, the reaction is faster and it is complete after 1 h. Cooling the solution at –40 °C allowed the isolation of complex [K_2_{[U(OSi(O^*t*^Bu)_3_)_3_]_2_(μ-NH)_2_}], **8** as a crystalline yellow solid in 75% yield. The ^1^H NMR signal assigned to the two amide protons is found at 176.5 ppm for a solution of **8** in d_8_-toluene and is absent in the ^1^H NMR spectrum of a solution of **8** reacted with D_2_. The solid-state structure of **8** presented in Fig. 6 shows the presence of an heterodimetallic complex (U_2_K_2_) in which the two U cations are held together by two imido (NH^2–^) bridging ligands. An inversion center is found at an equal distance between the U(iv) cations. The protons of the imido ligands can be crystallographically identified, making their assignment unambiguous. The U–N bond distances (2.192(3)–2.273(3) Å) in **8** are elongated with respect to the U–N_nitride_ bond distances in complexes **3** (2.018(12) Å, 2.086(11) Å) and **4** (2.101(6) and 2.022(5) Å), and are similar to those found in the only other reported bridging parent imido–U(iv) linkage in the complex [Cs{U(OSi(O^*t*^Bu)_3_)_3_}_2_(μ-H)(μ-NH)^6^ (U1–N1A = 2.231(7) Å and U2–N1A = 2.288(8), Å). The UN[combining circumflex]U angle (106.08(13)°) is the same as found present in complex **4** but the coordination geometry of the two uranium centers changes from distorted square pyramidal in **4** to distorted trigonal bipyramidal geometry in **8**. In spite of their importance as intermediates in dinitrogen conversion to ammonia only three examples of parent imido complexes of uranium were previously reported.4c,^6^,10m These results show that that the bis-nitride complex **4** promotes oxidative H_2_ cleavage affording a reduced imido-bridged U(iv) complex. The reaction pathway probably does not involve H_2_ coordination to the metal center but direct attack of H_2_ to the two nitrides as previously reported for a terminal iridium complex.2*a* The reactivity of complex **4** with H_2_ is remarkable and provides a model for the probable intermediates formed during the conversion of N_2_ and H_2_ into ammonia promoted by uranium nitrides in the Haber–Bosch process. Notably the reactivity of complex **4** shows that uranium bis-nitride complexes can activate dihydrogen in ambient conditions to form NH bonds.

When complex **4** is reacted with H_2_ at higher pressure (5.5 atm) ^1^H NMR spectroscopy shows the formation of a mixture of species among which **8** was not identified. However, the formation of ammonia could not be detected in this reaction.

The important role of nitride protonation in the biological formation of NH_3_ from N_2_ continues to motivate the study of the reaction of nitrides with proton donors.1c,2f–k,4c

^1^H NMR studies show that the protonation of both **4** and **8** with 1 equiv. of PyHOTf proceeds slowly in toluene solution and leads to a complicate mixture of species that could not be identified (see Fig. S44 and S45†).

The reactivity of complex **4** and complex **8** with excess acid was also investigated in order to gain insight into the possible ammonia formation from these U(v)–N containing species. The reactivity is shown in Scheme 3. After treating complexes **4** and **8** with an excess of a 2 M solution of HCl in diethyl ether a green solution was obtained. ^1^H NMR analysis of the products in d_6_-dmso (dimethylsulfone was added as an internal standard for quantification) showed that NH_4_Cl is formed in both cases in 78% and 73% conversion for **4** and **8** respectively. When complex **4** is reacted with an excess of water (30 equivalents) in THF, ^1^H NMR analysis shows that NH_4_Cl is formed in 18(1)% yield. Lower yields in ammonia were reported for a terminal U(v) nitride upon water addition (10%).10*l*

### Magnetic properties

**Fig. 7:**
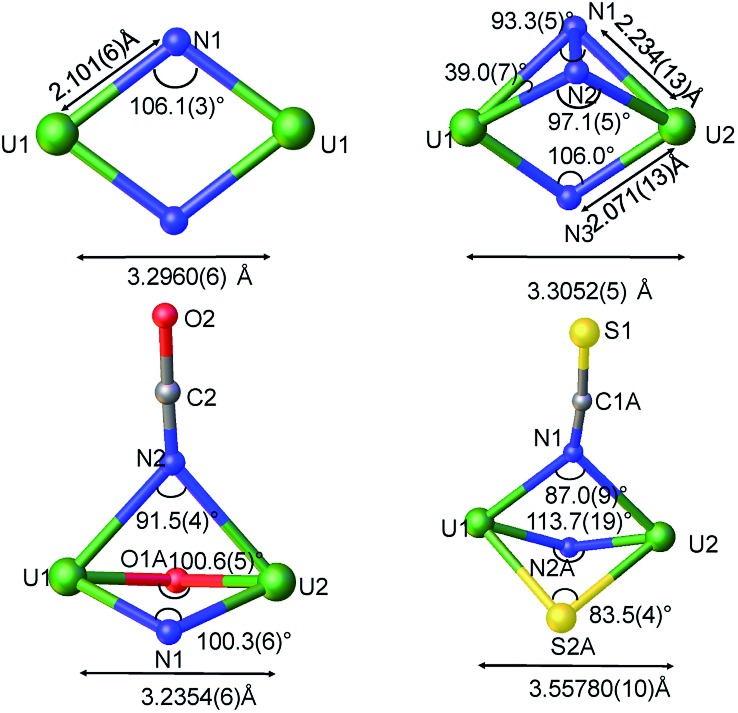
Ortep diagram of the core showing the metrical parameters for the bridging atoms in complexes **4**, **6**, **5,** and [K_3_{[U(OSi(O^*t*^Bu)_3_)_3_]_2_(μ-N)(μ-N_2_)}].4*c*

Examples of unambiguous magnetic coupling are rare in uranium chemistry, but RN-, N- or O- bridged U(v) compounds have shown antiferromagnetic interactions with values of the Neel temperature (*T*_N_, defined as the temperature at which the magnetic susceptibility reaches a maximum) ranging from 5 to 20 K.9b,15a–f The highest value of *T*_N_ at 110 K was reported by Cummins and Diaconescu for a arene-bridged U(iii) dimer.15h,i However, unusually strong antiferromagnetic exchange interactions with *T*_N_ of 70 K were reported for a bis-oxide bridged diuranium(v) complex with a diamond shaped [U^V^(μ-O)_2_U^V^] core.^16^ In view of the strong coupling exhibited by the diamond core shaped bis-oxo complex we decided to investigate if a strong exchange could be promoted by nitride ligands in a diamond shaped geometry (Fig. 7).

**Fig. 8:**
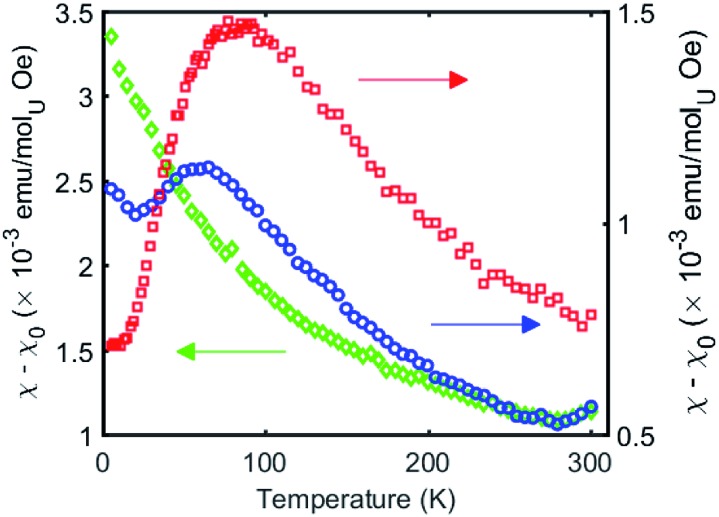
Temperature dependence of the magnetic susceptibility for complexes **4** (red), **5** (green), and **6** (blue) at 1000 Oe.

The *χ versus T* plot for complex **4** (Fig. 8) shows the magnetic behaviour of an antiferromagnetically coupled dinuclear complex with a *T*_N_ of approximately 77 K. Thus the diamond core [U^V^(μ-N)_2_U^V^] exhibits a strong coupling between the two f^1^ ions, slightly higher than that found in the bis-oxide complex [{((^nP,Me^ArO)_3_ tacn)U^V^}_2_(μ-O)_2_ ]^16^ (tacn = triazacyclononane, nP = neopentyl). A significantly weaker coupling with a *T*_N_ value of 10 K had been reported for the diuranium(v) nitride-, hydrazido bridged complex [K_3_{[U(OSi(O^*t*^Bu)_3_)_3_]_2_(μ-N)(μ-N_2_)}]^16^ where the two uranium cations are 3.3052(5) Å apart and present a [U^V^(μ-N_2_)(μ-N)(U^V^] diamond core. No EPR signal was observed for **4** in the solid state on in a 4.6 mM toluene solution. The absence of an EPR spectrum is indicative of a ±3/2 magnetic ground state.15*a*

In view of these differences we also investigated the magnetic properties of the other diuranium complexes presented here.

In complex **6** where one nitride in the [U^V^(μ-N)_2_U^V^] core is replaced by an oxide and a cyanate ligand the strong antiferromagnetic exchange is retained, with a slightly lower *T*_N_ of 60 K, as shown in Fig. 8. In contrast, the *χ versus T* plot for complex **5** (Fig. 8) does not show a clear maximum even if the presence of antiferromagnetic coupling cannot completely be ruled out.

Even in the latter case the magnetic coupling would be significantly less strong in the nitride, sulphide, thiocyanate bridged complex compared to complexes **4** and **6** probably as a result of the increased U–U distance (Fig. 8) and of a decreased orbital overlap. The presented results suggest that the nature of bridging atoms in dinuclear uranium complexes plays a key role in magnetic communication between uranium centers.

In spite of the fact that well characterized U(iv) complexes are much more common than their U(v) counterpart, only two examples of unambiguous magnetic coupling between two U(iv) ions have been reported.15g,^31^ Here we have investigated the diuranium(iv) complexes **2**, **3**, **7,** and **8** (see Fig. S55–S58 in ESI†). In none of these complexes the presence of unambiguous magnetic coupling could be identified. For complex **7** and for the bis-imido diuranium(iv) complex **8** the *χ versus T* plot shows a continuous increase until it reaches a plateau (at 10 K for **8** and at 90 K for **7**). While the possibility of antiferromagnetic coupling cannot be completely ruled out in these complexes the observed behaviour could be ascribed to the presence of temperature independent paramagnetism (TIP) due to low lying excited states. This behaviour is characteristic of magnetically isolated U(iv) complexes.10f,15g,^16^,^32^

The UV/vis/NIR electronic absorption spectra of complexes **3–8** were measured in toluene at different concentrations from 200 to 2500 nm at 25 °C (Fig. S60 and S61†). The NIR region (Fig. S61†) is dominated by metal-based f–f transitions with varying intensities. Well resolved absorptions with average molar extinction coefficients of ∼50 to 120 M^–1^ cm^–1^ can be observed for the U(v) complexes **4**, **5** and **6** in the 450–2250 nm region and are comparable to bands observed for bis-oxo diuranium(v) complexes^16^ and terminal U(v) oxo and nitride complexes.9a,^33^ In contrast complexes **3**, **7**, and **8** show several poorly resolved bands with low molar extinction coefficients of 10–50 M^–1^ cm^–1^ as expected for U(iv) complexes.^16^

Particularly remarkable is the difference in the magnetic properties between the [U^IV^(μ-NH)_2_U^IV^] and [U^V^(μ-N)_2_U^V^] cores, that present similar geometry and coordination environment although significantly larger U–U separation is found in the imido core (3.5687(2) Å) compared to the nitride one (3.2960(6) Å). This may be interpreted in term of a decreased covalency in the U(iv)–NH bonding compared to U(v)–N bonds and of a lower orbital overlap of the imido compared to the nitride ligand resulting in the absence of magnetic interaction. Notably, recent computational work on related systems showed the presence of a markedly covalent character of the U–N–U bond in nitride bridged diuranium(iii) and diuranium(v) complexes while no orbital overlap was found between the bridging oxide and the two uranium centers in analogous oxide-bridged diuranium complexes.9*b*

It should however be noted that unambiguous magnetic exchange coupling between uranium(iv) centers is more rarely observed15g,^31^ than for uranium(v) and may be masked by other phenomena^34^ rendering difficult the comparison between U(v) and U(iv) systems.

## Concluding remarks

In conclusion, we reported a reproducible route for the synthesis of a diuranium(v) bis nitride bridged complex and the first reactivity study of a [U^V^(μ-N)_2_U^V^] core. The [U^V^(μ-N)_2_U^V^] complex reacts readily with 1 equivalent of CO_2_ and CS_2_ in ambient conditions leading to C–O and C–S bond cleavage and N–C bond formation. Thus, the nucleophilic attack of one nitride to the heteroallenes occurs without redox changes at the metal center leading to chalcogenido- and cyanato-bridged diuranium(v) complexes. The cleavage of the C–O bond appears favoured by the [U^V^(μ-N)_2_U^V^] core, compared to the analogous diuranium(iv) nitride which undergoes preferentially addition of two CO_2_ molecules to afford a dicarbamate.5*g* The reaction with CS_2_ afford the first example of sulphide bridged diuranium(v) complex which shows high stability against disproportionation. The reaction of the [U^V^(μ-N)_2_U^V^] core with CO results in a different concomitant reactivity for the two bridging nitrides that leads both to the complete CO cleavage and transfer of one nitride to form CN^–^ and to the reductive carbonylation of a nitride to afford a cyanate bridged diuranium(iv) complex. The reported reactivity demonstrated the high nucleophilicity of the two nitride groups in a diamond [U^V^(μ-N)_2_U^V^] core suggesting that such species can provide novel routes to the use of easily available C1 substrates like CO and CO_2_ in the synthesis of valuable chemicals. The [U^V^(μ-N)_2_U^V^] core can also irreversibly effect the oxidative cleavage of H_2_ in ambient conditions affording a bis-imido bridged diuranium(iv) species that is relevant as a model for intermediates in the uranium mediated transformation of N_2_ and H_2_ into ammonia. The conversion of the nitrides into ammonia was not achieved with H_2_ but ammonia could be obtained readily in nearly quantitative yield from the addition of a strong acid to the nitride. Beside the relevance of diuranium complexes reported here in N-transfer reactivity, they also provided a platform for interrogating electronic communication between uranium centers. No unambiguous magnetic coupling was identified for the reported diuranium(iv) complexes including the bis-imido [U^IV^(μ-NH)_2_U^IV^] species. In contrast, the magnetic data measured for the [U^V^(μ-N)_2_U^V^] complex show an unusually strong antiferromagnetic coupling between the uranium ions. The coupling is maintained in the nitride-oxo cyanate bridged diuranium(v) complex, but absent in the analogous nitride–sulphide thiocyanate compound probably as a result of the increased U–U distance and decreased orbital overlap. In summary uranium bis-nitrides not only provide competent models for species involved in dinitrogen activation, but demonstrated to be attractive for promoting N-transfer reactivity and implement magnetic interaction between uranium centers.

## Experimental

### General procedures

Unless otherwise noted, all manipulations were carried out at ambient temperature under an inert atmosphere using Schlenk techniques and/or an MBraun glovebox equipped with a purifier unit. The water and oxygen levels were always kept lower than 0.1 ppm. Glassware was dried at 150 °C prior to use.

**Caution!** Depleted uranium (primary isotope ^238^U) is a weak α-emitter (4.197 MeV) with a half-life of 4.47 × 10^9^ years. Manipulations and reactions should be performed in monitored fumehoods or in an inert atmosphere glovebox in a radiation laboratory equipped with α-counting equipment.

### Starting materials

Unless otherwise noted, reagents were purchased from commercial suppliers and used without further purification. The solvents were purchased from Aldrich or Cortecnet (deuterated solvents) in their anhydrous form, conditioned under argon and vacuum distilled from K/benzophenone (toluene and tetrahydrofuran (THF)). DMSO-d_6_ was freeze-degassed and stored over activated 3 Å molecular sieves. The complex [U(OSi(O^*t*^Bu)_3_)_3_]_2,_**1** was prepared according to the published procedure.^17^ Carbon monoxide (N47 Bt-S 10/200) was purchased from Carbagas and transferred prior to use in a flask in equipped with a Young valve and containing activated 3 Å molecular sieves. ^13^CO (93.13% ^13^C) was purchased from Cortecnet and transferred prior to use in a flask equipped with a Young valve and containing activated 3 Å molecular sieves. Hydrogen gas (99.9999% purity) and carbon dioxide (99.9999% purity) were purchased from Carbagas. ^13^CO_2_ (93.13% ^13^C) and D_2_ (99.8% D) were purchased from Cortecnet and transferred prior to use in a flask in equipped with a Young valve and containing activated 3 Å molecular sieves. KN_3_ was purchased from Sigma and dried under high vacuum prior to use.

### NMR, UV and IR spectroscopy

NMR spectra were performed in J. Young tubes. ^1^H and ^13^C NMR spectra were recorded on a Bruker 400 MHz spectrometer. Quantitative ^13^C NMR were recorded on a Bruker 600 MHz. NMR chemical shifts are reported in ppm and were referenced to the residual ^1^H and ^13^C signals of the deuterated solvents.

IR spectra were recorded with a Perkin Elmer 1600 Series FTIR spectrophotometer flushed with argon. The UV-Vis-NIR spectra were performed with a Perkin Elmer Lambda 750 instrument. The spectra were recorded at 25 °C in 1 mm cells adapted with J. Young valves for 3.8–8.3 mM toluene solutions of complexes **3–8**.

### Elemental analyses

Samples were analysed under nitrogen by the elemental analyses department of the EPFL using a Thermo Scientific Flash 2000 Organic Elemental Analyzer.

### Magnetic data

Magnetic measurements were performed using a Quantum Design MPMS-5T superconducting quantum interference device (SQUID) magnetometer in a temperature range 2–300 K. The powder sample was enclosed in an evacuated quartz capsule and placed inside a plastic straw.

### X-ray analyses

Bragg-intensities of 2, 3, and 7 were measured at low temperature (see Table 1 in ESI†) using MoKα radiation on a Bruker APEX II CCD kappa diffractometer. The datasets were reduced by EvalCCD^35^ and corrected for absorption by modelling an empirical transmission surface as sampled by multiple symmetry-equivalent and/or azimuth rotation-equivalent intensity measurements by real spherical harmonic functions of even order.^36^

The X-ray diffraction data of **5**, **6** and **8** were collected at low temperature (see Table 1 in ESI†) using CuKα radiation for **5** and **6**, and MoKα radiation for **8**. A Rigaku SuperNova dual system diffractometer with an Atlas S2 CCD detector was used for compounds **5** and **6**, and one equipped with an Atlas CCD detector for compound **8**. The datasets were reduced and corrected for absorption, with the help of a set of faces enclosing the crystals as snugly as possible, with CrysAlis^Pro^.^37^

The solutions and refinements for the structures were performed by the latest available version of ShelXT^38^ and ShelXL.^39^ All non-hydrogen atoms were refined anisotropically using full-matrix least-squares based on |*F*|^2^. The hydrogen atoms were placed at calculated positions by means of the “riding” model where each H-atom was assigned a fixed isotropic displacement parameter with a value equal to 1.2 U_eq_ of its parent C-atom (1.5 U_eq_ for the methyl groups), except the hydrogen atom of the bridging imido ligand in **8**, which was found in a difference map and refined freely. Crystallographic and refinement data are summarized in Table 1 in ESI.† In the structures of **2**, **3**, **5** and **6**, one disordered toluene solvent molecule per formula unit was removed with the help of the solvent-masking program in OLEX2.^40^ In the structure of **3**, both bridging ligands, namely nitrido and azido bridges were disordered over two positions found in a difference Fourier map and refined anisotropically yielding an occupancy of one half each. RIGU restraint was applied to the displacement parameters of all atoms. In case of **5**, the bridging ligands, namely a nitride and a sulphido ligand, were disordered over two orientations found in a difference map. The major and minor parts were refined anisotropically, but distance restraint (SADI) had to be applied to the nitrido ligand for a convergent least-square refinement, yielding site occupancy ratios of 0.67(2)/0.33(2). SIMU restraint was applied to the displacement parameters of all light atoms. The structure of **6** was refined as a two-component inversion twin. The core of this complex was not disordered, but RIGU and SIMU restrains were applied to the displacement parameters of all atoms and all light atoms, respectively. In the structure of **7**, the oxygen atom of the cyanate bridging ligand was disordered over two positions found in a difference Fourier map and refined anisotropically yielding an occupancy of one half each. The distance restraints such as DFIX and SADI were used for a convergent least-squares refinement of the disordered isocyanido ligand. RIGU and SIMU restrains were applied to the displacement parameters of all atoms and all light atoms, respectively.

### Synthesis of [K{[U(OSi(O^*t*^Bu)_3_)_3_]_2_(μ-N)}], **2**

A cold (–40 °C) solution of, **1** (182.9 mg, 0.089 mmol, 1 equiv.) in 3 mL of THF was added onto cold (–40 °C) KN_3_ (7.2 mg, 0.089 mmol, 1 equiv.), and the reaction mixture was vigorously stirred with a glass-coated stirring bar for 4 d at –40 °C. ^1^H NMR analysis of the reaction mixture revealed the presence of [K_2_{[U(OSi(O^*t*^Bu)_3_)_3_]_2_(μ-N)(μ-N_3_)}], **3**, starting material, and [K{[U(OSi(O^*t*^Bu)_3_)_3_]_2_(μ-N)}], **2**. Successive recrystallizations in THF at –40 °C afforded the complex [K{[U(OSi(O^*t*^Bu)_3_)_3_]_2_(μ-N)}], **2** as a purple-brown crystalline solid in 21% yield (40 mg, 0.019 mmol). ^1^H NMR (400 MHz, d_8_-THF, 298 K): *δ* = –0.67 (s, 162H, CH_3_, terminal siloxide). Anal. calcd for **2** C_72_H_162_KNO_24_Si_6_U_2_: C: 40.99%; H: 7.74%; N: 0.66%. Found: C: 41.03%; H: 8.05%; N: 0.78%.

### Synthesis of [K_2_{[U(OSi(O^*t*^Bu)_3_)_3_]_2_(μ-N)(μ-N_3_)}], **3**

A cold (–40 °C) dark brown solution of **1** (99.2 mg, 0.048 mmol, 1 equiv.) in 3 mL of THF was added onto cold (–40 °C) KN_3_ (7.8 mg, 0.097 mmol, 2 equiv.), and the reaction mixture was vigorously stirred with a glass-coated stir bar for 5 d at –40 °C. The resulting brown solution was filtered on a microfilter and volatiles were removed under vacuum. The residue was dissolved in 1 mL of toluene and left standing for 3 d at –40 °C, affording the complex [K_2_{[U(OSi(O^*t*^Bu)_3_)_3_]_2_(μ-N)(μ-N_3_)}], **3** as a brown crystalline solid (81.8 mg, 0.035 mmol, 72%). ^1^H NMR (400 MHz, d_8_-THF, 298 K): *δ* = –1.59 ppm (s, 162H, CH_3_, terminal siloxide), ^1^H NMR (400 MHz, d_8_-toluene, 298 K): *δ* = –1.39 ppm (s, 162H, CH_3_, terminal siloxide).

Anal. calcd for **3**·1.5 toluene C_72_H_162_N_4_O_24_K_2_Si_6_U_2_·(C_7_H_8_)_1.5_: C: 42.55%; H: 7.53%; N: 2.41%. Found: C: 42.38%; H: 7.94%; N: 2.01%.

### Synthesis of [K_2_{[U(OSi(O^*t*^Bu)_3_)_3_]_2_(μ-N)_2_}], **4**

A dark brown solution of **3** (197.3 mg, 0.09 mmol) in 4.5 mL of toluene was degassed and heated up to 70 °C for 24 h. N_2_ bubbling was observed. The solution was then evaporated (2 mL) and left standing overnight at –40 °C, affording the complex [K_2_{[U(OSi(O^*t*^Bu)_3_)_3_]_2_(μ-N)_2_}], **4** as a dark brown crystalline solid (142.8 mg, 0.066 mmol, 73% yield). ^1^H NMR (400 MHz, d_8_-toluene, 298 K): *δ* = –1.76 ppm (s, 162H, CH_3_, terminal siloxide).

Anal. calcd for **4**·0.3 toluene C_72_H_162_N_2_O_24_K_2_Si_6_U_2_·(C_7_H_8_)_0.3_: C: 40.63%; H: 7.57%; N: 1.28%. Found: C: 40.60%; H: 7.40%; N: 1.08%.

### Synthesis of [K_2_{[U(OSi(O^*t*^Bu)_3_)_3_]_2_(μ-N)(μ-S)(μ-NCS)}], **5**

1.3 μL of CS_2_ were added to a dark brown solution of **4** (47.0 mg, 0.0217 mmol, 1 equiv.) in 3 mL of toluene. The reaction mixture was stirred at room temperature overnight. The reaction mixture was then evaporated (1 mL) and left standing overnight at –40 °C, affording the precipitation of complex [K_2_{[U(OSi(O^*t*^Bu)_3_)_3_]_2_(μ-N)(μ-S)(μ-NCS)}], **5** as an orange-brown crystalline solid (35.2 mg, 0.0158 mmol, 73% yield). The ^13^C labelled complex ^13^C-**5** was prepared with the same procedure from ^13^CS_2_. ^1^H NMR (400 MHz, d_8_-toluene, 298 K): *δ* = 0.39 ppm (s, 162H, CH_3_, terminal siloxide). ^13^C NMR of ^13^C-**5** (400 MHz, d_8_-toluene, 298 K): *δ*(ppm) = 132.06 (N^13^CS), 73.18 (*C*(CH_3_)_3_), 31.96 (C(*C*H_3_)_3_). The ^13^C NMR spectrum in d_6_-dmso shows a resonance at 129.41 ppm, which is assigned to KN^13^CS and confirms that the thiocyanate ligand is present in the complex **5** and is released in dmso (Fig. S14†).

IR of **5**: 1977 cm^–1^ (*ν*_CN_); IR of ^13^C-**5**: 1931 cm^–1^ (*ν*_CN_). Anal. calcd for **5**·1.3 toluene C_73_H_162_N_2_O_24_K_2_S_2_Si_6_U_2_·(C_7_H_8_)_1.3_ C: 41.81%; H: 7.37%; N: 1.19%. Found C: 41.85%; H: 7.57%; N: 1.47%.

### Synthesis of [K_2_{[U(OSi(O^*t*^Bu)_3_)_3_]_2_(μ-N)(μ-O)(μ-NCO)}], **6**

A dark brown solution of **4** (42.7 mg, 0.0197 mmol, 1 equiv.) in 3 mL of toluene was degassed by freeze-pump-thawing three times and CO_2_ (1 equiv.) was added inside the reaction tube. The solution was brought back to room temperature and became ochre yellow. The solution was then evaporated (1.5 mL) and left standing overnight at –40 °C, to yield the complex [K_2_{[U(OSi(O^*t*^Bu)_3_)_3_]_2_(μ-N)(μ-O)(μ-NCO)}], **6** as an ochre yellow crystalline solid (33.5 mg, 0.015 mmol, 78% yield). The ^13^C labelled complex ^13^C-**6** was prepared with the same procedure from ^13^CO_2_.

^1^H NMR (400 MHz, d_8_-toluene, 298 K): *δ* = 0.18 ppm (s, 162H, CH_3_, terminal siloxide). ^13^C NMR of ^13^C-**6** (400 MHz, d_8_-toluene, 298 K): *δ*(ppm) = 143.06 (N^13^CO), 72.74 (*C*(CH_3_)_3_), 30.98 (C(*C*H_3_)_3_).

The ^13^C NMR spectrum in D_2_O shows a resonance at 129.93 ppm. This confirms the presence in the complex of the cyanate ligand, which is extruded in D_2_O as KN^13^CO.

IR of **6**: 2192 cm^–1^ (*ν*_CN_); IR of ^13^C-**6**: 2133 cm^–1^ (*ν*_CN_). Anal. calcd for **6** C_73_H_162_N_2_O_26_K_2_Si_6_U_2_ C: 39.73%; H: 7.40%; N: 1.27%; S: 2.86% found C: 40.04%; H: 7.45%; N: 1.26%; S: 2.59%.

### Synthesis of [K_2_{[U(OSi(O^*t*^Bu)_3_)_3_]_2_(μ-CN)(μ-O)(μ-NCO)}], **7**

A dark brown solution of **4** (42.2 mg, 0.0192 mmol, 1 equiv.) in 2 mL of toluene was degassed by freeze-pump-thawing three times and CO (3 equiv.) was added inside the reaction tube. The solution immediately became light green. The solution was then evaporated (1 mL) and left standing at –40 °C overnight, affording the precipitation of complex [K_2_{[U(OSi(O^*t*^Bu)_3_)_3_]_2_(μ-CN)(μ-O)(μ-NCO)}], **7** as a light green crystalline solid (28.4 mg, 0.0127 mmol, 67% yield). The ^13^C labelled complex ^13^C-**7** was prepared with the same procedure from ^13^CO. ^1^H NMR (400 MHz, d_8_-toluene, 298 K): *δ* = 0.77 ppm (s, 162H, CH_3_, terminal siloxide). ^13^C NMR of ^13^C-**7** (400 MHz, d_8_-toluene, 298 K): *δ*(ppm) = 214.19 (N^13^CO or ^13^CN), 73.12 (*C*(CH_3_)_3_), 30.72 (C(*C*H_3_)_3_), –158.48 ((N^13^CO or ^13^CN). Anal. calcd for **7** C_74_H_162_N_2_O_26_K_2_Si_6_U_2_ C: 40.06%; H: 7.36%; N: 1.26% found C: 40.02%; H: 7.67%; N: 1.18%

The quantitative ^13^C-NMR spectrum of isolated crystals of ^13^C-**6** in d_6_-dmso shows two peaks at 166.94 and 126.60 in a 1 : 1 ratio, assigned respectively, to K^13^CN and KN^13^CO released from the complex, confirming the presence and the nature of the two bridging ligands.

### Synthesis of [K_2_{[U(OSi(O^*t*^Bu)_3_)_3_]_2_(μ-NH)_2_}], **8**

A dark brown solution of **4** (49.9 mg, 0.023 mmol, 1 equiv.) in 3 mL of toluene was degassed by freeze-pump-thawing three times. 1 atm of H_2_ was added. The reaction mixture was kept at 60 °C for 1 h. The solution became yellow. The solution was then evaporated (1 mL) and left standing at –40 °C overnight affording the complex [K_2_{[U(OSi(O^*t*^Bu)_3_)_3_]_2_(μ-NH)_2_}], **8** as a yellow crystalline solid (37.4 mg, 0.017 mmol, 75% yield).

^1^H NMR (400 MHz, d_8_-toluene, 298 K): *δ* = –0.87 ppm (s, 162H, CH_3_, terminal siloxide), 176.46 (s, 2H, N*H*).

^13^C NMR of **8** (400 MHz, d_8_-toluene, 298 K): *δ*(ppm) = 71.00 (*C*(CH_3_)_3_), 22.71 (C(*C*H_3_)_3_). Anal. calcd for **8** C_72_H_164_N_2_O_24_K_2_Si_6_U_2_ C: 39.95%; H: 7.64%; N: 1.29% found C: 40.32%; H: 7.47%; N: 1.34%.

Complex **4** also reacts slowly with Me_3_Si–SiMe_3_ but the reaction leads to multiple products (Fig. S47†) that were not further characterized.

### Addition of excess HCl(Et_2_O) to [K_2_{[U(OSi(O^*t*^Bu)_3_)_3_]_2_(μ-N)_2_}], **4**

200 μL of a 2 M solution of HCl in Et_2_O were added to **4** (5.1 mg, 0.00236 mmol, 1 equiv.). The solution turned immediately light green and a light green/white precipitate was formed. After 10 minutes, volatiles were removed under vacuum. The resulting solid was dissolved in d_6_-dmso and dimethylsulfone was added as an internal standard for the quantitative NH_4_Cl detection. NH_4_Cl is formed in 78% yield (complete transformation of both nitrides in ammonia being 100% conversion).

### Addition of excess HCl(Et_2_O) to [K_2_{[U(OSi(O^*t*^Bu)_3_)_3_]_2_(μ-NH)_2_}], **8**

200 μL of a 2 M solution of HCl in Et_2_O were added to **8** (6.9 mg, 0.00319 mmol, 1 equiv.). The solution turned immediately light green and a light green/white precipitate was formed. After 10 minutes, the volatiles were removed under vacuum. The resulting solid was dissolved in d_6_-dmso and dimethylsulfone was added as an internal standard for the quantitative NH_4_Cl detection. NH_4_Cl is formed with 72% yield.

### Addition of excess HCl(Et_2_O) to [K_2_{[U(OSi(O^*t*^Bu)_3_)_3_]_2_(μ-N)_2_}], **4** in presence of KC_8_

A suspension of KC_8_ (10.3 mg, 0.075 mmol, 10 equiv.) in 0.5 mL of toluene was added to a solution of **4** (16.2 mg, 0.0075 mmol, 1 equiv.). The reaction mixture was stirred for 10 minutes at RT. The volatiles were removed under vacuum and 400 μL of a 2 M solution of HCl in Et_2_O were added to the solid residue. The reaction mixture was stirred for 10 minutes.

Volatiles were removed under vacuum. The resulting solid was dissolved in d_6_-dmso and dimethylsulfone was added as an internal standard for the quantitative NH_4_Cl detection. NH_4_Cl is formed with 75% yield.

### Addition of H_2_ to [K_2_{[U(OSi(O^*t*^Bu)_3_)_3_]_2_(μ-N)_2_}], **4**

5.5 bar of H_2_ were added to dark brown solution of **4** (12.2 mg, 0.006 mmol, 1 equiv.) in 0.5 mL of toluene. The reaction mixture immediately became yellow. The reaction mixture was left stirring at RT for 3 h. The headspace and the volatiles were vacuum transferred onto a frozen solution of 2 M HCl in Et_2_O. The resulting solution was allowed to thaw and volatiles were removed *in vacuo*. The residue was dissolved in d_6_-dmso but no NH_4_Cl could be detected by ^1^H NMR spectroscopy.

### Reactivity of [K_2_{[U(OSi(O^*t*^Bu)_3_)_3_]_2_(μ-N)_2_}], **4** with 30 equivalents of H_2_O

A cold (–40 °C) solution **4** (7.9 mg, 0.0037 mmol, 1 equiv.) in 1.5 mL of THF was added to a frozen 0.5 M solution of H_2_O in THF (220 μL, 0.11 mmol, 30 equiv.). The reaction mixture was brought to room temperature and was left stirring for 2 d. The headspace and the volatiles were vacuum transferred onto 1 mL of a frozen 2 M solution of HCl in Et_2_O. The solution was thawed and stirred at room temperature for 2 h. The formation of a white precipitate of NH_4_Cl was observed. The volatiles were removed *in vacuo* and the resulting solid residue was dissolved in 0.5 mL of d_6_-dmso and dimethylsulfone was added as an internal standard for the quantitative NH_4_Cl detection. NH_4_Cl is formed with 18(1)% yield.

## Conflicts of interest

There are no conflicts to declare.

## Supplementary Material

Supplementary information

Crystal structure data
