# Correction: Facile N-functionalization and strong magnetic communication in a diuranium(v) bis-nitride complex

**DOI:** 10.1039/c9sc90049g

**Published:** 2019-03-06

**Authors:** Luciano Barluzzi, Lucile Chatelain, Farzaneh Fadaei-Tirani, Ivica Zivkovic, Marinella Mazzanti

**Affiliations:** a Institut des Sciences et Ingénierie Chimiques , Ecole Polytechnique Fédérale de Lausanne (EPFL) , CH-1015 Lausanne , Switzerland . Email: marinella.mazzanti@epfl.ch; b Laboratory for Quantum Magnetism , Institute of Physics , Ecole Polytechnique Fédérale de Lausanne (EPFL) , CH-1015 Lausanne , Switzerland

## Abstract

Correction for ‘Facile N-functionalization and strong magnetic communication in a diuranium(v) bis-nitride complex’ by Luciano Barluzzi *et al.*, *Chem. Sci.*, 2019, DOI: ; 10.1039/c8sc05721d.



## 

The authors regret that Scheme 2 is incorrect in the original manuscript. The correct scheme is displayed below.

**Scheme 2:**
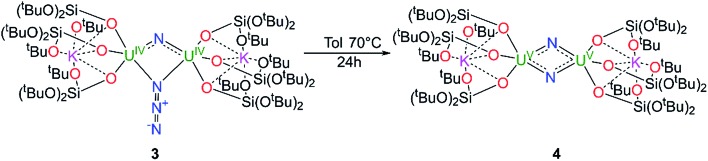
Synthesis of complex [K_2_{[U(OSi(O^*t*^Bu)_3_)_3_]_2_(μ-N)_2_}], **4**.

The Royal Society of Chemistry apologises for these errors and any consequent inconvenience to authors and readers.

