# Supplementary material for: Facile N-functionalization and strong magnetic communication in a diuranium(v) bis-nitride complex
Source: Chem Sci. 2019 Feb 18;10(12):3543–55. doi: 10.1039/c8sc05721d (PMC6438153; doi:10.1039/c8sc05721d)
Supplement: Supplementary file 1 [file SC-010-C8SC05721D-s001.pdf]

Electronic Supplementary Material (ESI) for Chemical Science.
This journal is © The Royal Society of Chemistry 2019

Supporting information for the manuscript

Facile N-functionalization and strong magnetic communication in a diuranium(V) bis-nitride complex

Luciano Barluzzi[a], Lucile Chatelain[a], Farzaneh Fadaei-Tirani[a], Ivica Zivkovic[b] and Marinella Mazzanti*[a]

[a]Institut des Sciences et Ingénierie Chimiques, Ecole Polytechnique Fédérale de Lausanne (EPFL), CH-1015 Lausanne, Switzerland.
[b]Laboratory for Quantum Magnetism, Institute of Physics, Ecole Polytechnique Fédérale de Lausanne (EPFL), CH-1015 Lausanne, Switzerland.

Contents

A) NMR SPECTRA 3

B) IR SPECTRA 26

C) X-RAY CRYSTALLOGRAPHIC DATA 28

D) MAGNETIC DATA 30

E) UV-VIS DATA 35

A) NMR spectra

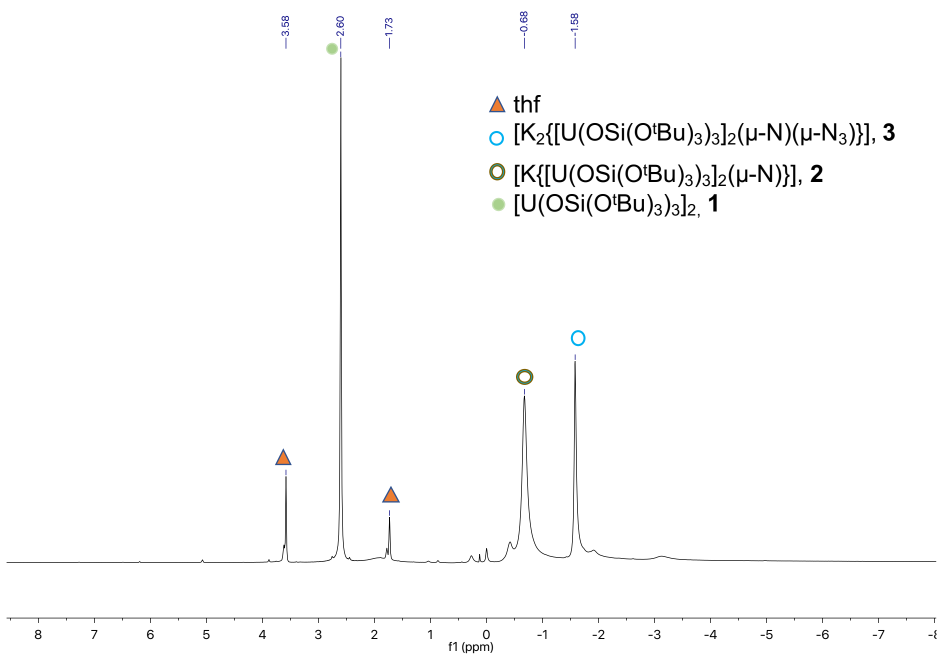

***Fig. S1:** ^{1}H-NMR (400 MHz) at 298 K in d_8-thf of the crude reaction mixture between $[U(OSi(O^tBu)_3)_3]_2$, 1 and one equivalent of KN_3 in thf after 4 days at -40°C leading to a mixture of complex $[K\{[U(OSi(O^tBu)_3)_3]_2(\mu\text{-}N)\}]$, 2 and complex $[K_2\{[U(OSi(O^tBu)_3)_3]_2(\mu\text{-}N)(\mu\text{-}N_3)\}]$, 3*

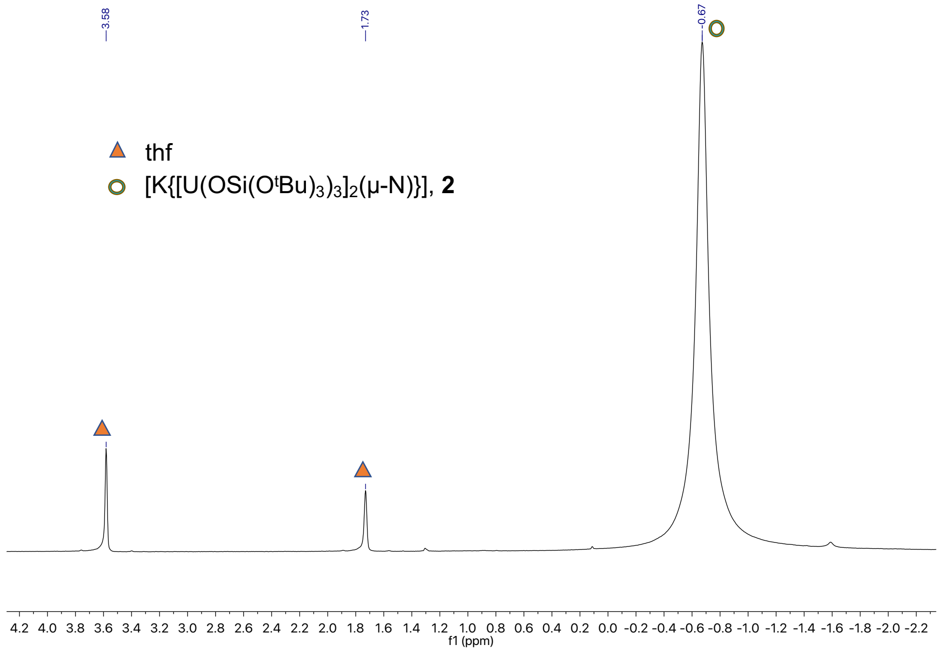

***Fig. S2:** ^{1}H-NMR (400 MHz) at 298 K in d_8-thf of isolated $[K\{[U(OSi(O^tBu)_3)_3]_2(\mu\text{-}N)\}]$, 2*

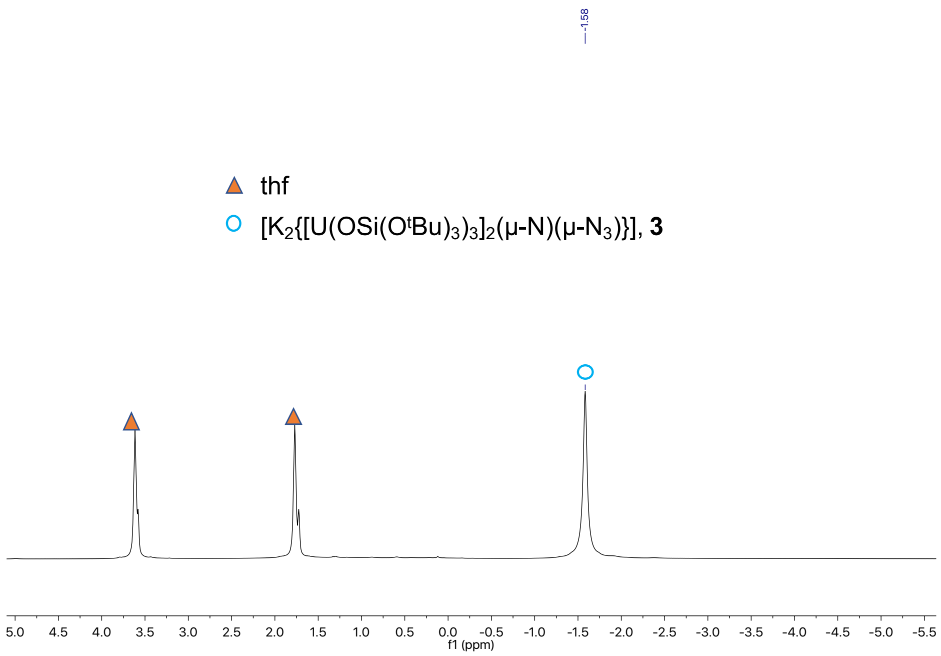

Fig. S3: ^{1}H-NMR (400 MHz) at 298 K in d_8-thf of the reaction mixture between [U(OSi(O^{t}Bu)$_3$)$_3$]$_2$, 1 and two equivalents of KN_3 in thf at -40°C for 5 days forming complex [K$_2${[U(OSi(O^{t}Bu)$_3$)$_3$]$_2$(μ-N)(μ-N$_3$)}], 3

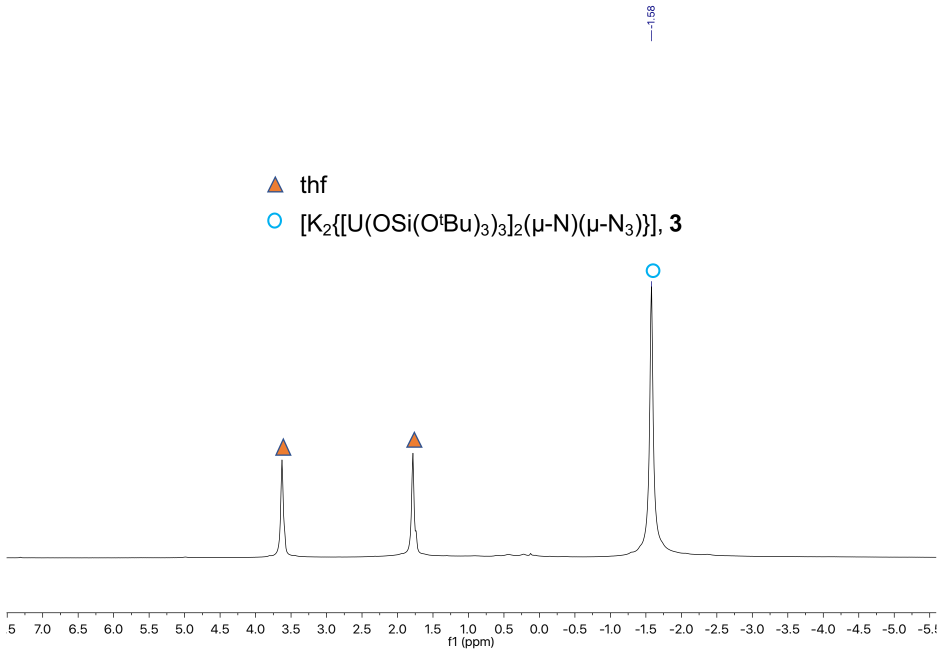

Fig. S4: ^{1}H-NMR (400 MHz) at 298 K in d_8-thf of isolated [K$_2${[U(OSi(O^{t}Bu)$_3$)$_3$]$_2$(μ-N)(μ-N$_3$)}], 3

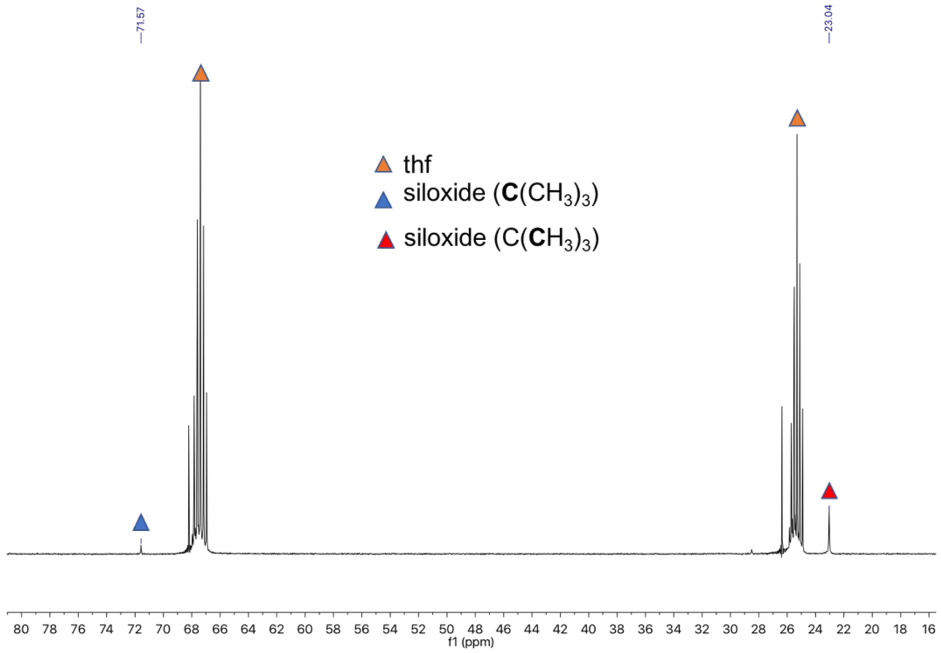

Fig. S5: ^{13}C-NMR (400 MHz) at 298 K in d_8-thf of isolated [K_2{[U(OSi(O^{t}Bu)$_3$)$_3$]$_2$(μ-N)(μ-N_3)}], 3

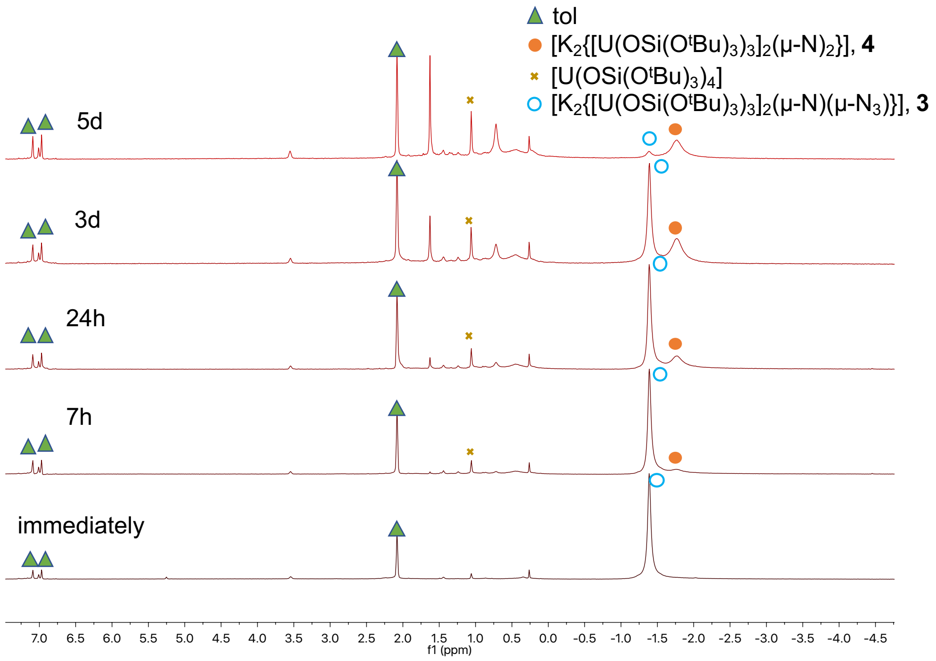

Fig. S6: Evolution of the ^{1}H-NMR (400 MHz) at 298 K in d_8-tol of isolated [K_2{[U(OSi(O^{t}Bu)$_3$)$_3$]$_2$(μ-N)(μ-N_3)}], 3 at room temperature

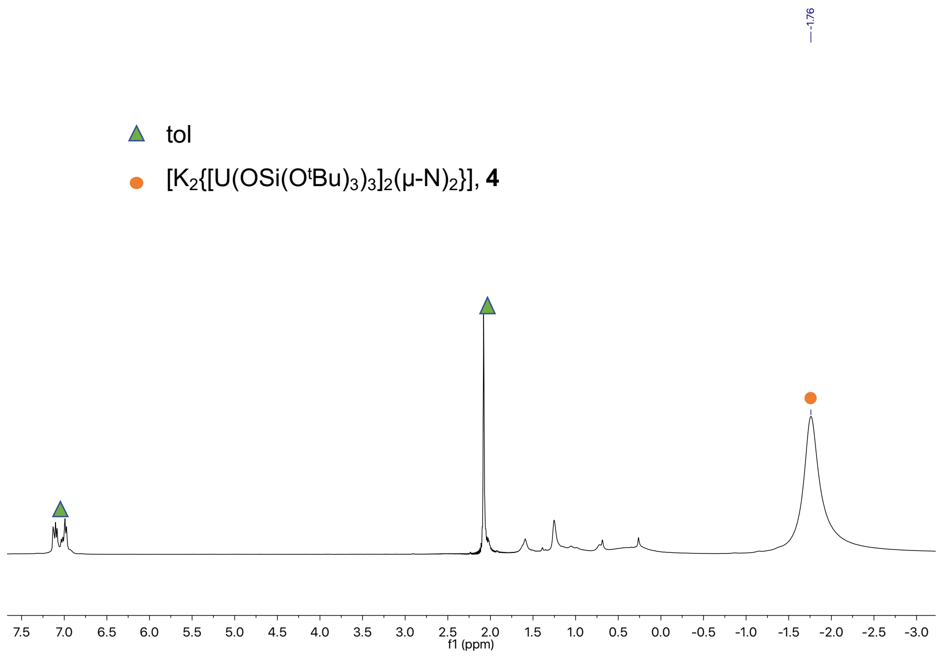

***Fig. S7:** ^{1}H-NMR (400 MHz) at 298 K in d_8-tol of isolated [K_2{[U(OSi($O^tBu)_3)_3]_2$(μ-N)(μ-N_3)}], 3 after 24h at 70°C affording complex [K_2{[U(OSi($O^tBu)_3)_3]_2$(μ-N)$_2$}], 4*

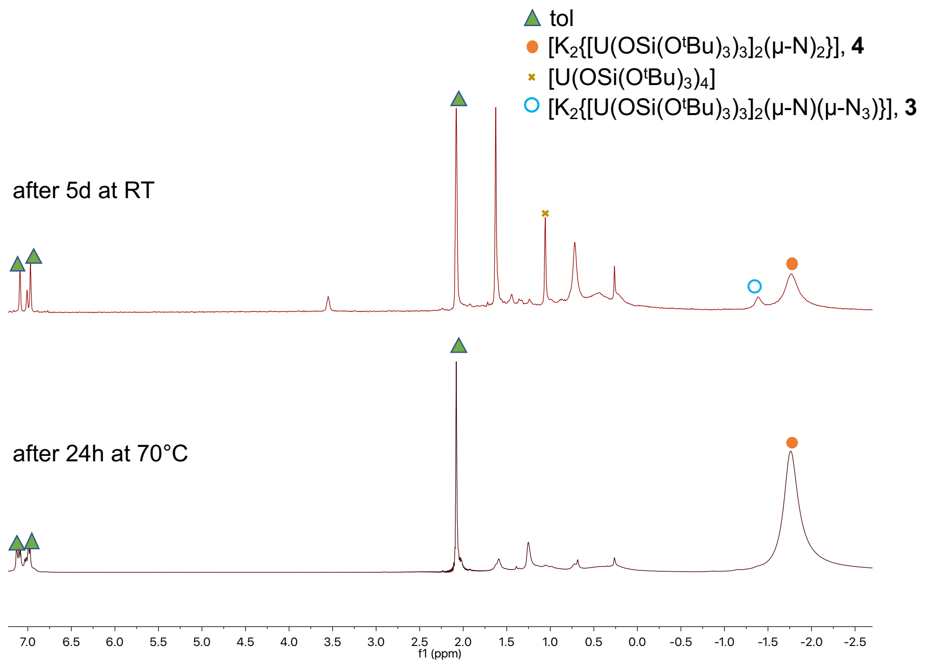

***Fig. S8:** ^{1}H-NMR (400 MHz) at 298 K in d_8-tol of [K_2{[U(OSi($O^tBu)_3)_3]_2$(μ-N)(μ-N_3)}], 3, after 5 days at RT (top) and after 24h at 70°C (bottom).*

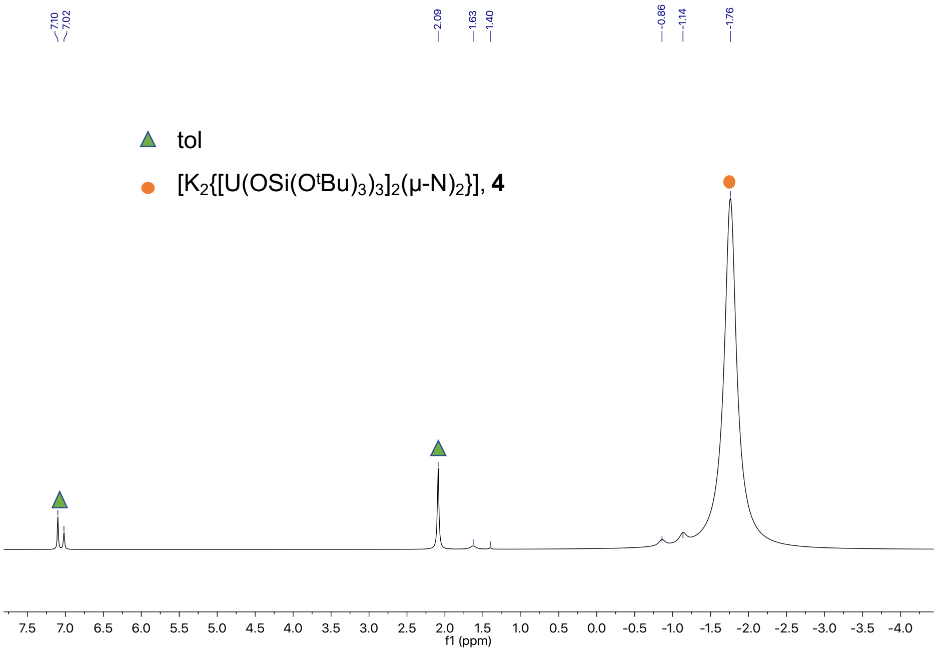

Fig. S9: ^{1}H-NMR (400 MHz) at 298 K in d_8-tol of isolated [K_2{[U(OSi(O^tBu)$_3$)$_3$]$_2$(μ-N)$_2$}], 4

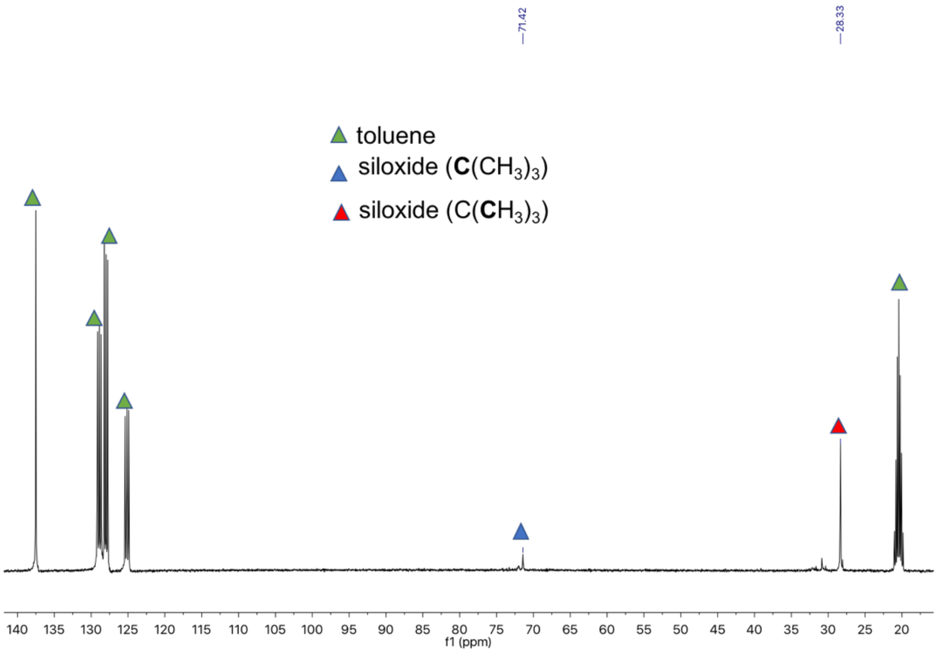

Fig. S10: ^{13}C-NMR (400 MHz) at 298 K in d_8-tol of isolated [K_2{[U(OSi(O^tBu)$_3$)$_3$]$_2$(μ-N)$_2$}], 4

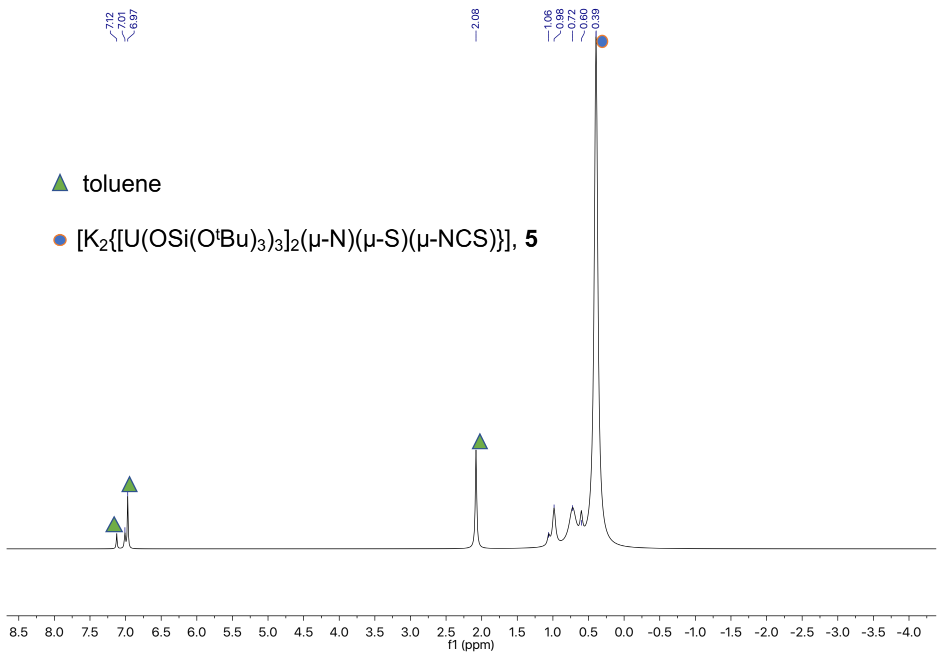

Fig. S11: ^{1}H-NMR (400 MHz) at 298 K in d_8-tol of the reaction mixture 6h after the addition of one equivalent of CS_2 to $[K_2\{[U(OSi(O^tBu)_3)_3]_2(\mu\text{-}N)_2\}]$, 4 affording complex $[K_2\{[U(OSi(O^tBu)_3)_3]_2(\mu\text{-}N)(\mu\text{-}S)(\mu\text{-}NCS)\}]$, 5

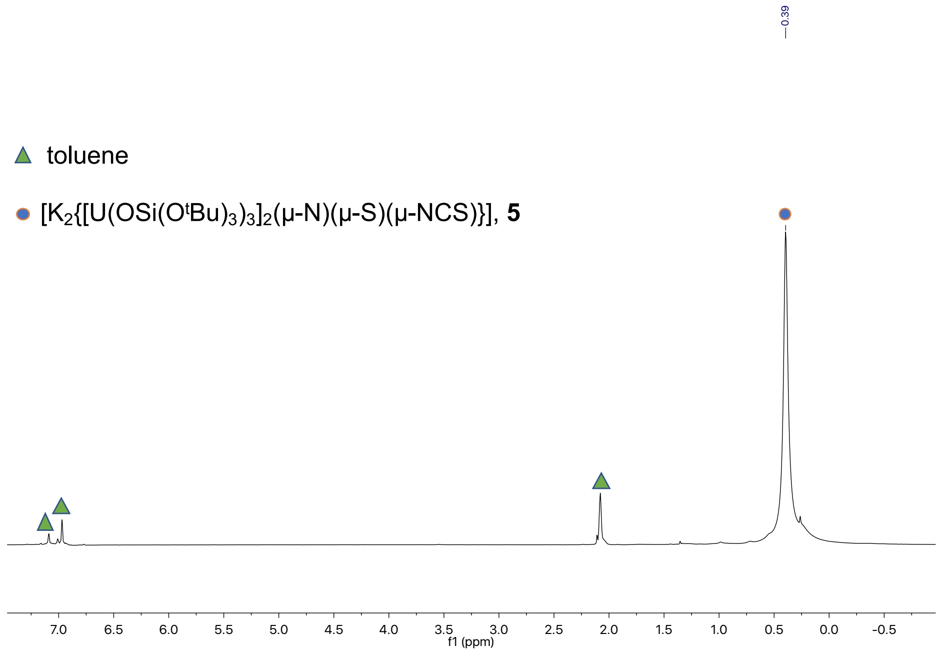

Fig. S12: ^{1}H-NMR (400 MHz) at 298 K in d_8-tol of isolated $[K_2\{[U(OSi(O^tBu)_3)_3]_2(\mu\text{-}N)(\mu\text{-}S)(\mu\text{-}NCS)\}]$, 5

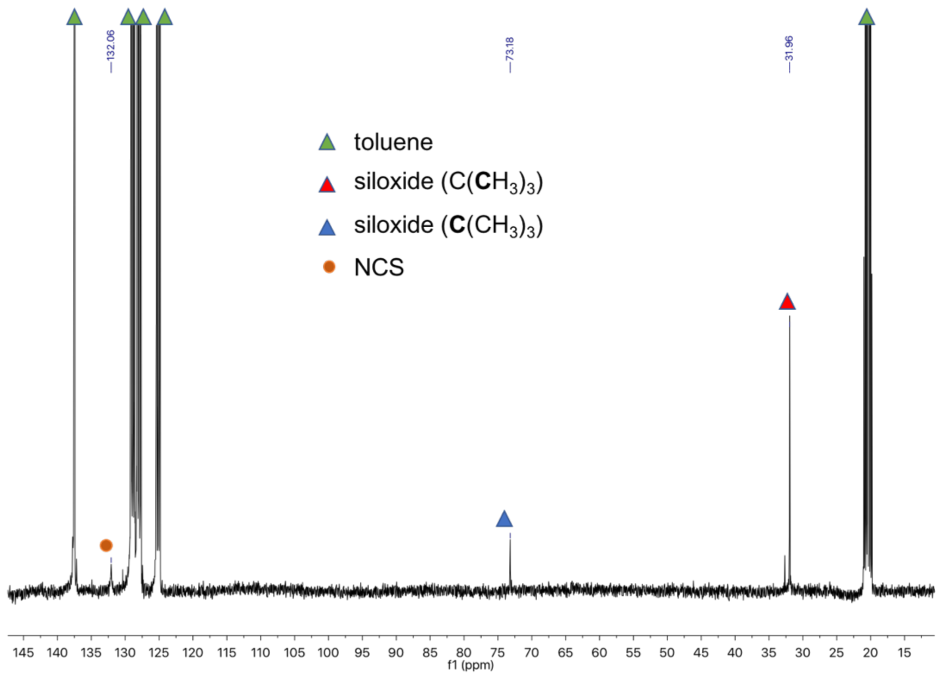

Fig. S13: ^{13}C-NMR (400 MHz) at 298 K in d_8-tol of isolated [K_2{[U(OSi(O^{t}Bu)$_3$)$_3$]$_2$(μ-N)(μ-S)(μ-N^{13}CS)}], ^{13}C-5

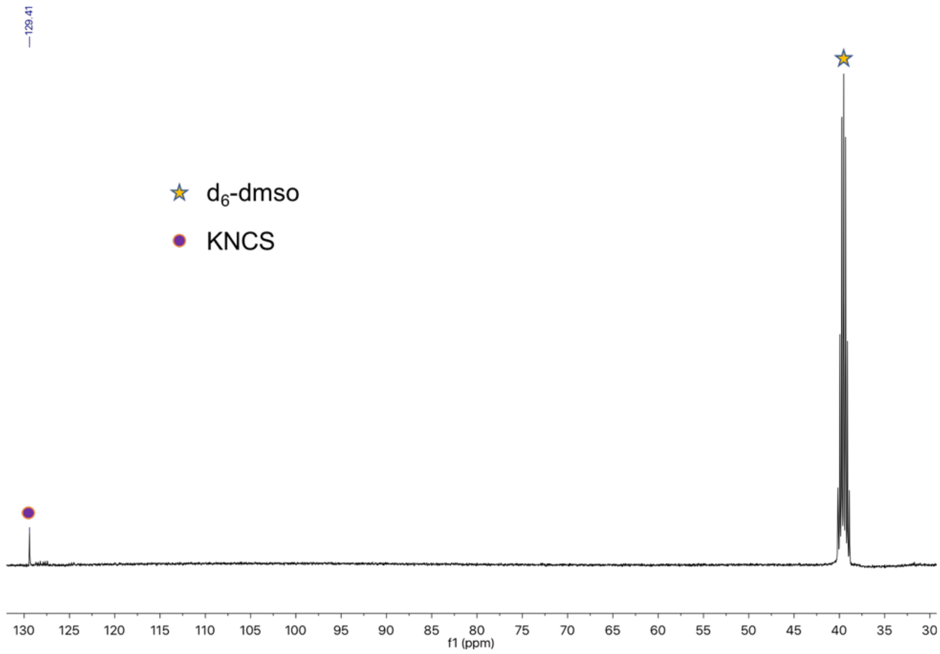

Fig. S14: ^{13}C-NMR (400MHz) at 298 K in d_6-dmso of isolated [K_2{[U(OSi(O^{t}Bu)$_3$)$_3$]$_2$(μ-N)(μ-S)(μ-N^{13}CS)}], ^{13}C-5

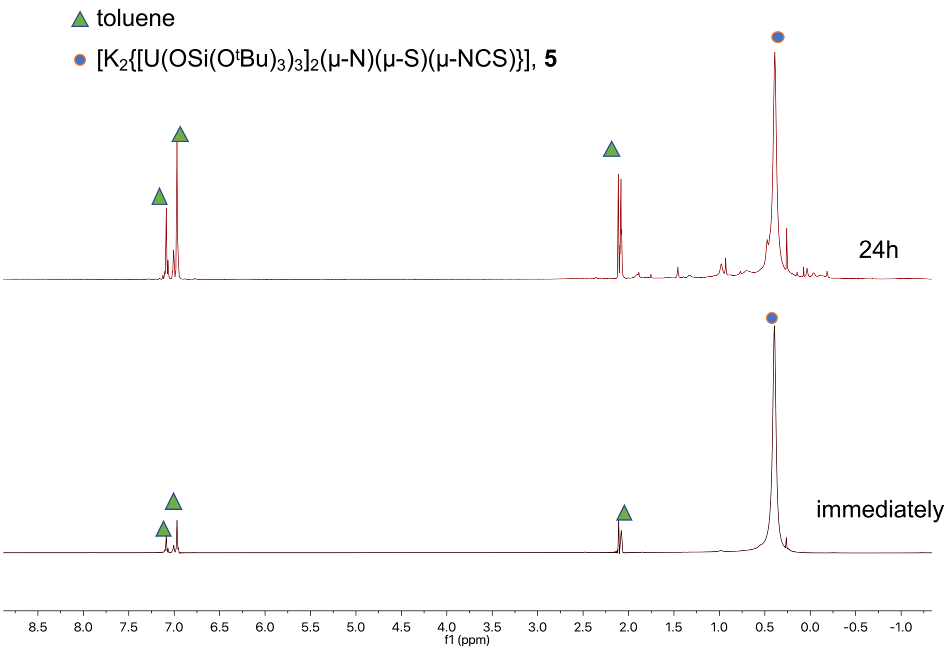

Fig. S15: ***Evolution of the ^{1}H-NMR (400 MHz) at 298 K in d_8-tol of the reaction mixture between $[K_2\{[U(OSi(O^tBu)_3)_3]_2(\mu\text{-}N)_2\}]$, 4 and 10 equivalents of $^{13}CS_2$ affording complex $[K_2\{[U(OSi(O^tBu)_3)_3]_2(\mu\text{-}N)(\mu\text{-}S)(\mu\text{-}N^{13}CS)\}]$, ^{13}C-5 and an unidentified precipitate***

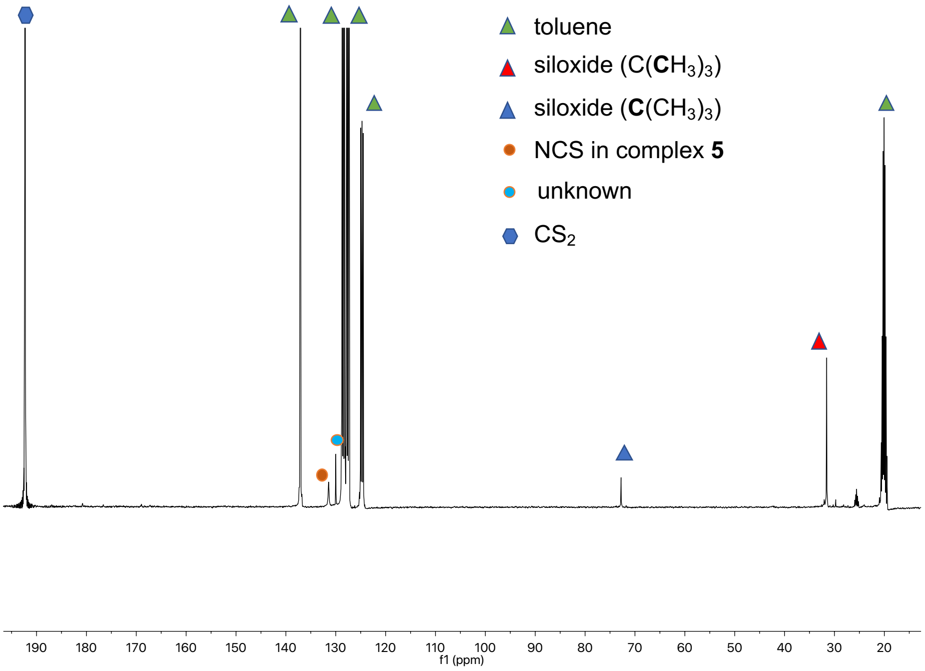

Fig. S16: ***^{13}C-NMR (400 MHz) at 298 K in d_8-tol of the reaction mixture between $[K_2\{[U(OSi(O^tBu)_3)_3]_2(\mu\text{-}N)_2\}]$, 4 and 10 equivalents of $^{13}CS_2$***

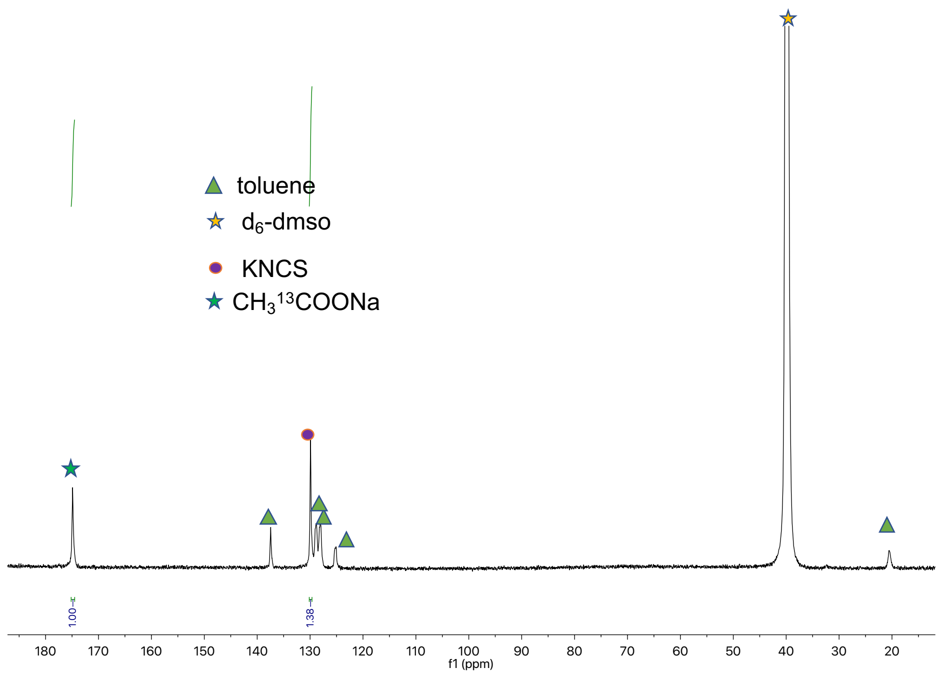

Fig. S17: ***Quantitative*** ^{13}***C-NMR (600 MHz) at 298 K in*** d_6***-dmso of the reaction mixture between*** $[K_2\{[U(OSi(O^tBu)_3)_3]_2(\mu\text{-}N)_2\}]$***, 4 and 10 equivalents of*** $^{13}CS_2$

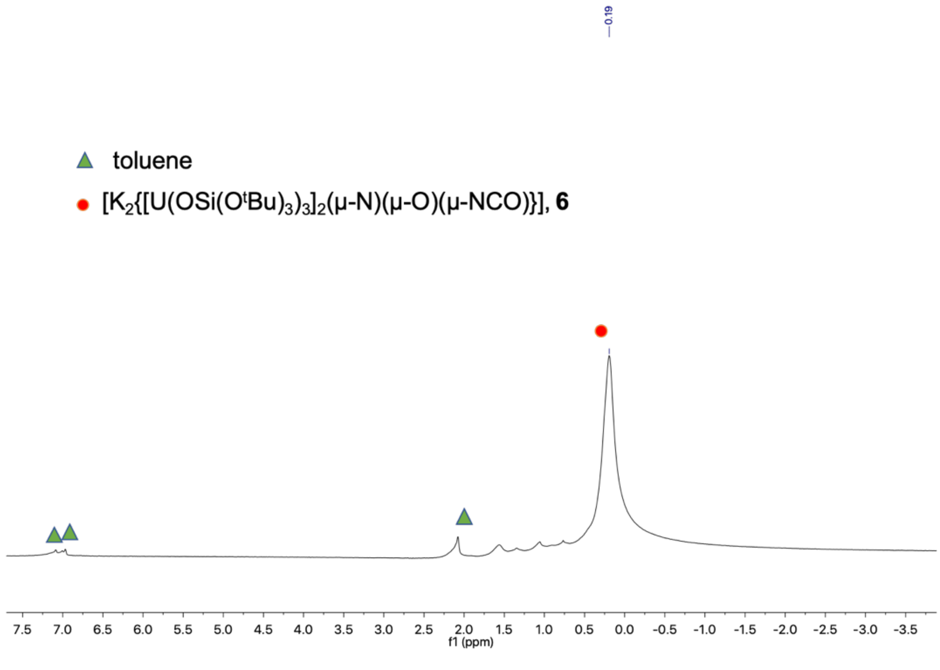

Fig. S18: ^{1}***H-NMR (400 MHz) at 298 K in*** d_8***-tol of the reaction mixture immediately after the addition of one equivalent of*** CO_2 ***to*** $[K_2\{[U(OSi(O^tBu)_3)_3]_2(\mu\text{-}N)_2\}]$***, 4 affording complex*** $[K_2\{[U(OSi(O^tBu)_3)_3]_2(\mu\text{-}N)(\mu\text{-}O)(\mu\text{-}NCO)\}]$***, 6***

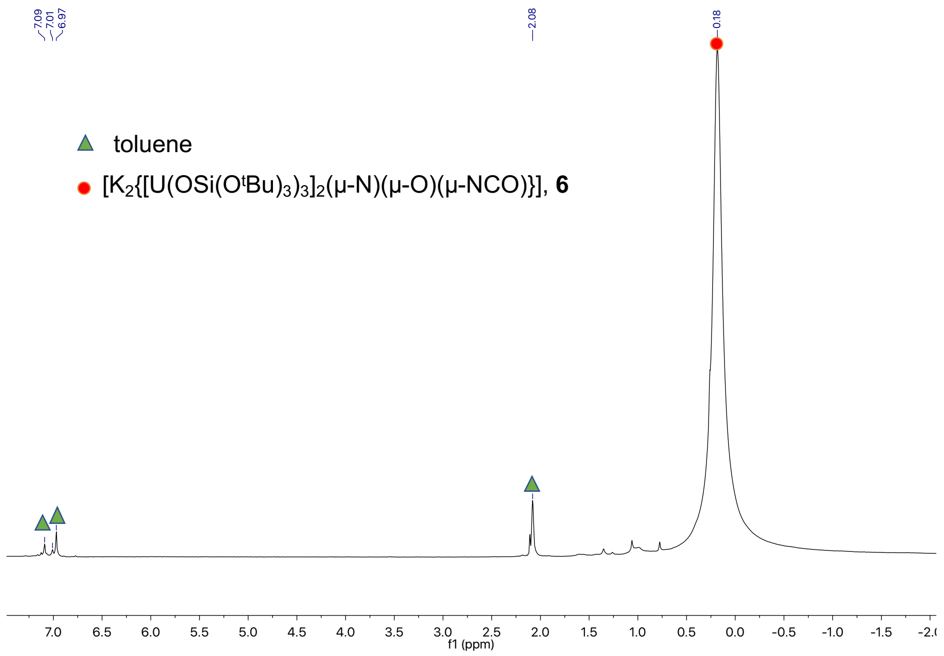

***Fig. S19:** ¹H-NMR (400 MHz) at 298 K in d_8-tol of isolated [K_2{[U(OSi($O^tBu)_3)_3]_2$(μ-N)(μ-O)(μ-NCO)}], 6*

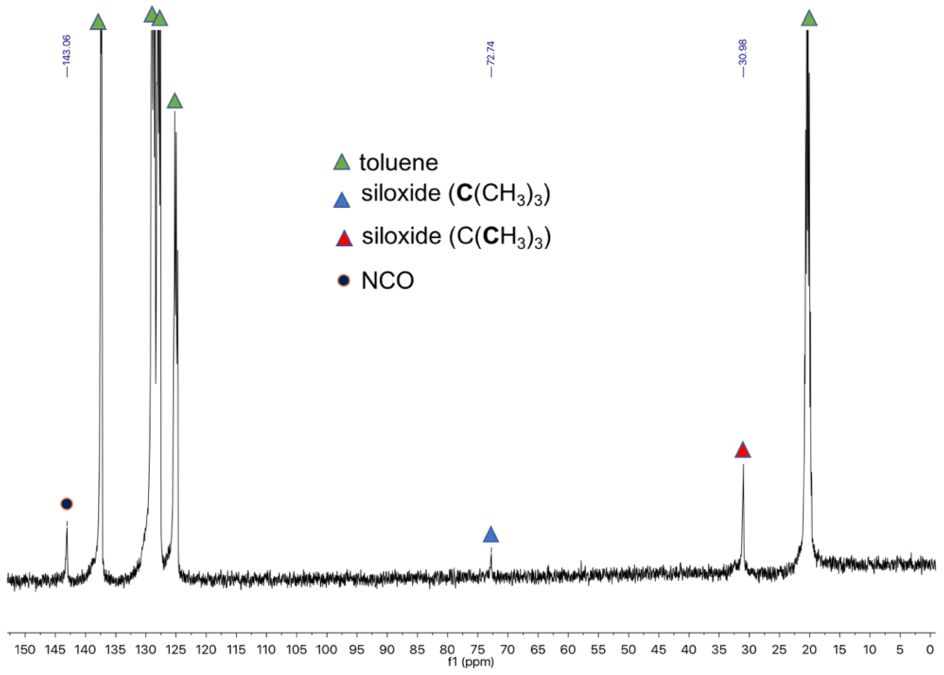

***Fig. S20:** ¹³C-NMR (400 MHz) at 298 K in d_8-tol of isolated [K_2{[U(OSi($O^tBu)_3)_3]_2$(μ-N)(μ-O)(μ-$N^{13}CO$)}], ¹³C-6*

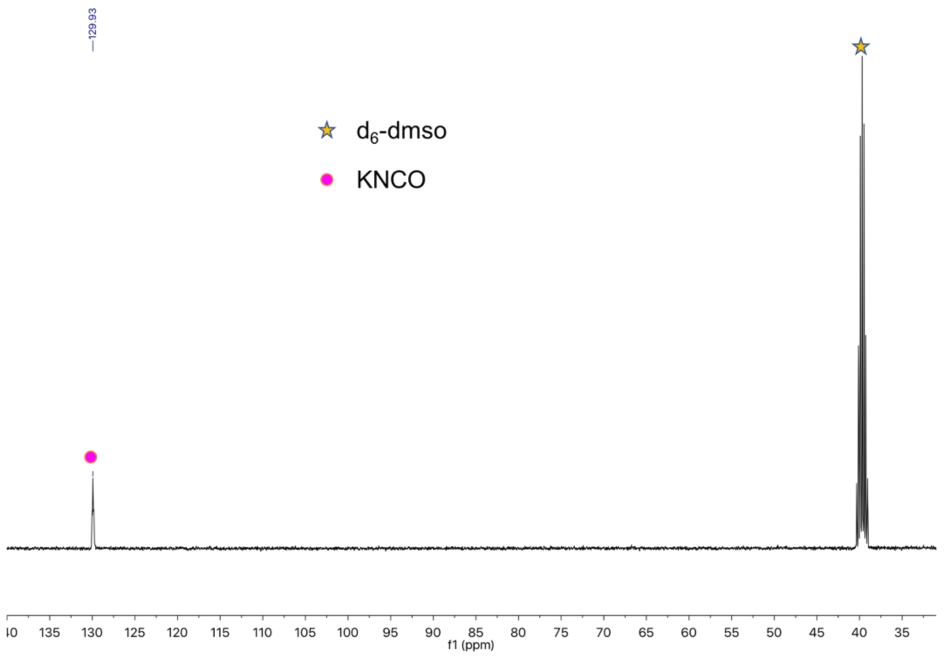

Fig. S21: ^{13}C-NMR (400 MHz) at 298 K in D_2O using d_6-dmso as a reference of isolated [K_2{[U(OSi(O^{t}Bu)$_3$)$_3$]$_2$(μ-N)(μ-O)(μ-N^{13}CO)}], ^{13}C-6

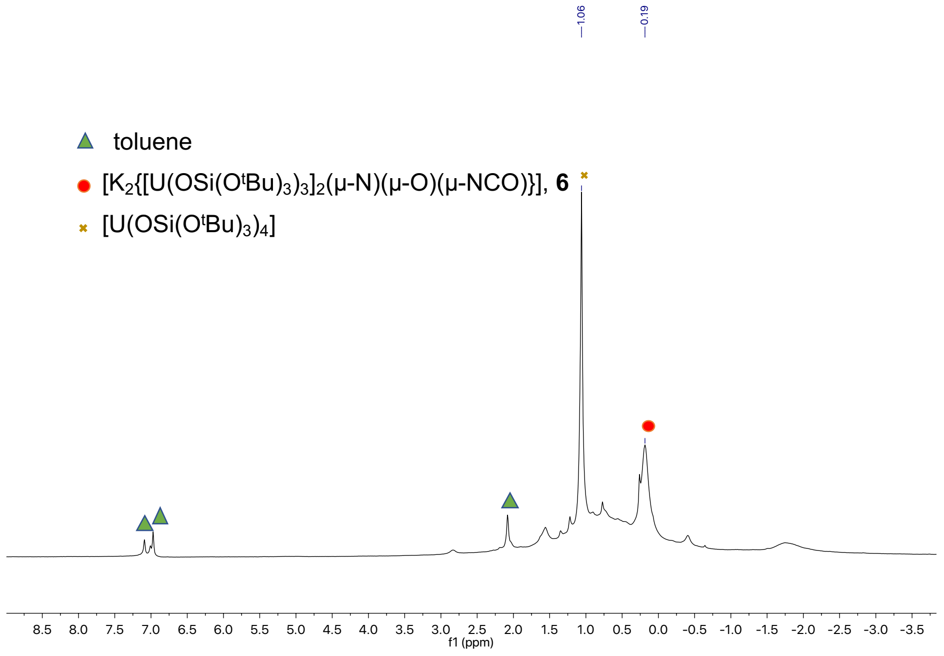

Fig. S22: ^{1}H-NMR (400 MHz) at 298 K in d_8-tol of the reaction mixture immediately after the addition of 2 equivalents of CO_2 to complex [K_2{[U(OSi(O^{t}Bu)$_3$)$_3$]$_2$(μ-N)$_2$}], 4

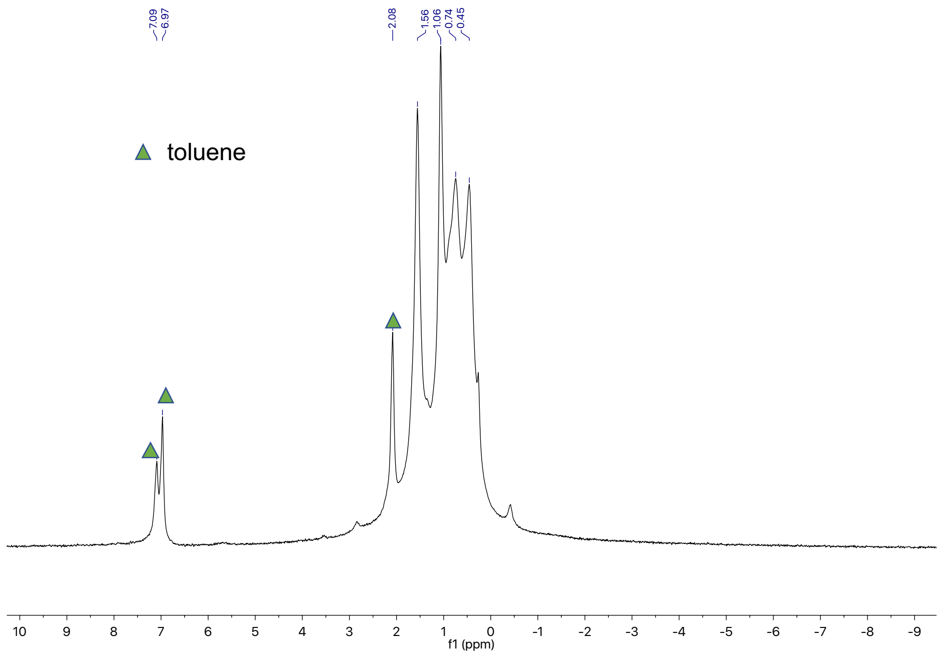

Fig. S23: ^{1}H-NMR (400 MHz) at 298 K in d_8-tol of the reaction mixture immediately after the addition of 10 equivalents of $^{13}CO_2$ to complex $[K_2\{[U(OSi(O^tBu)_3)_3]_2(\mu\text{-}N)_2\}]$, 4

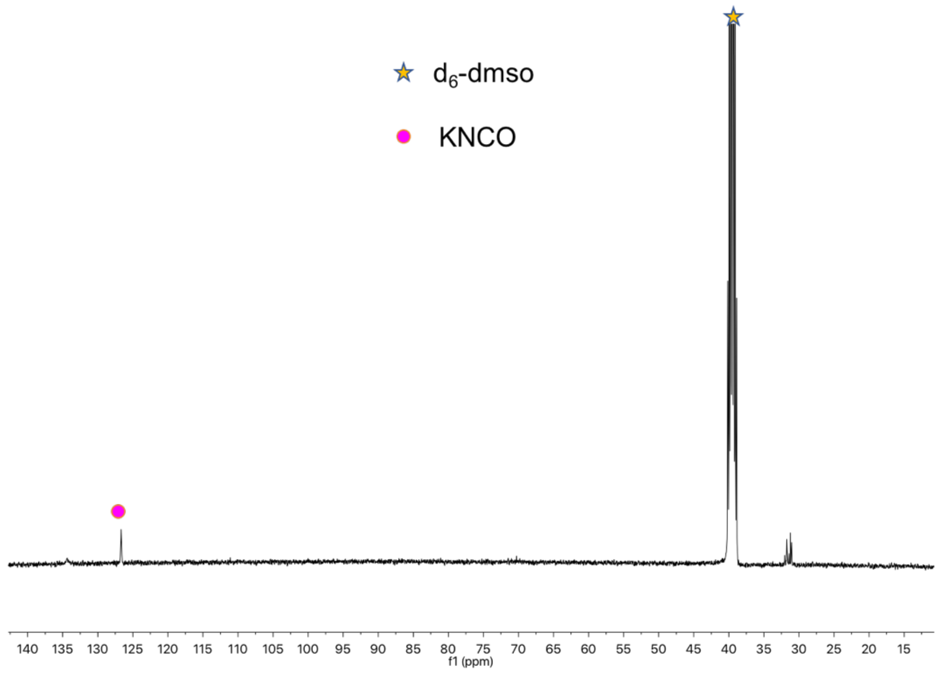

Fig. S24: ^{13}C-NMR (400 MHz) at 298 K in d_6-dmso of the reaction mixture after the addition of 10 eqvs of $^{13}CO_2$ to complex $[K_2\{[U(OSi(O^tBu)_3)_3]_2(\mu\text{-}N)_2\}]$, 4

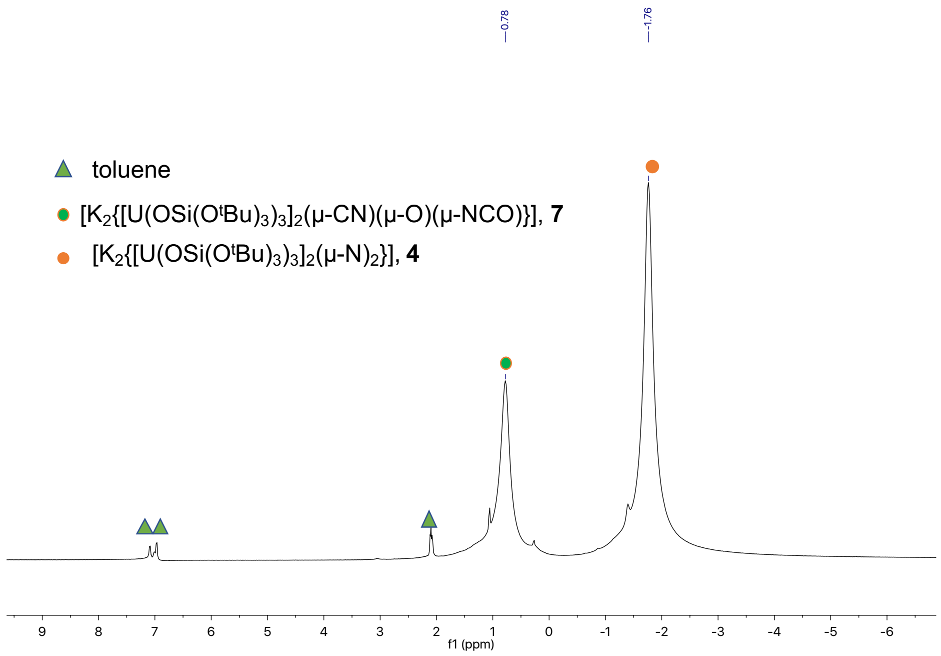

***Fig. S25:** ^{1}H-NMR (400 MHz) at 298 K in d_8-tol of the reaction mixture immediately after the addition of 1 eqv of CO to complex $[K_2\{[U(OSi(O^tBu)_3)_3]_2(\mu\text{-}N)_2\}]$, 4*

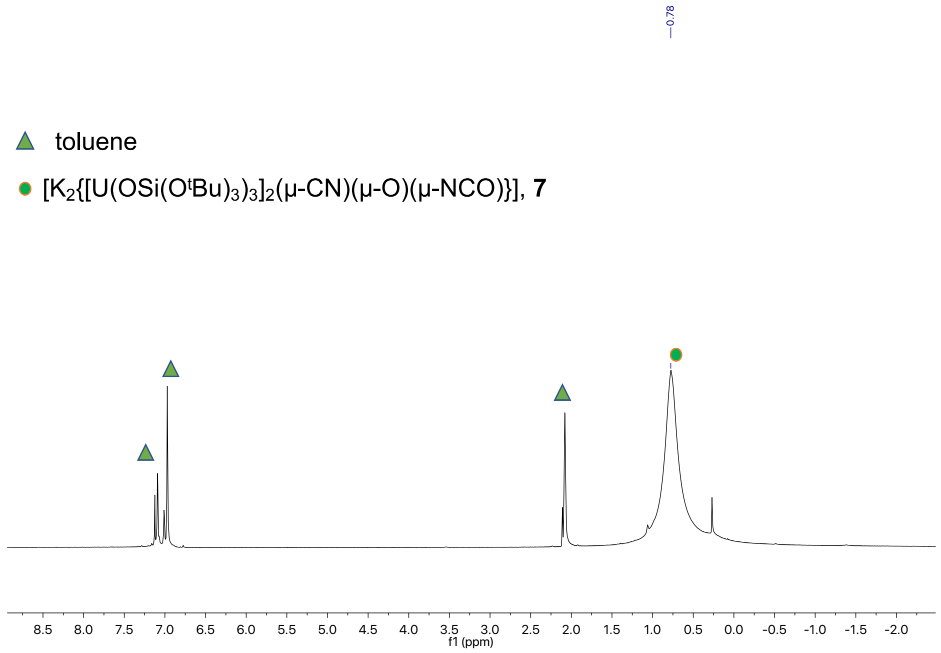

***Fig. S26:** ^{1}H-NMR (400 MHz) at 298 K in d_8-tol of the reaction mixture immediately after the addition of 3 eqvs of CO to complex $[K_2\{[U(OSi(O^tBu)_3)_3]_2(\mu\text{-}N)_2\}]$, 4 affording complex $[K_2\{[U(OSi(O^tBu)_3)_3]_2(\mu\text{-}CN)(\mu\text{-}O)(\mu\text{-}NCO)\}]$, 7*

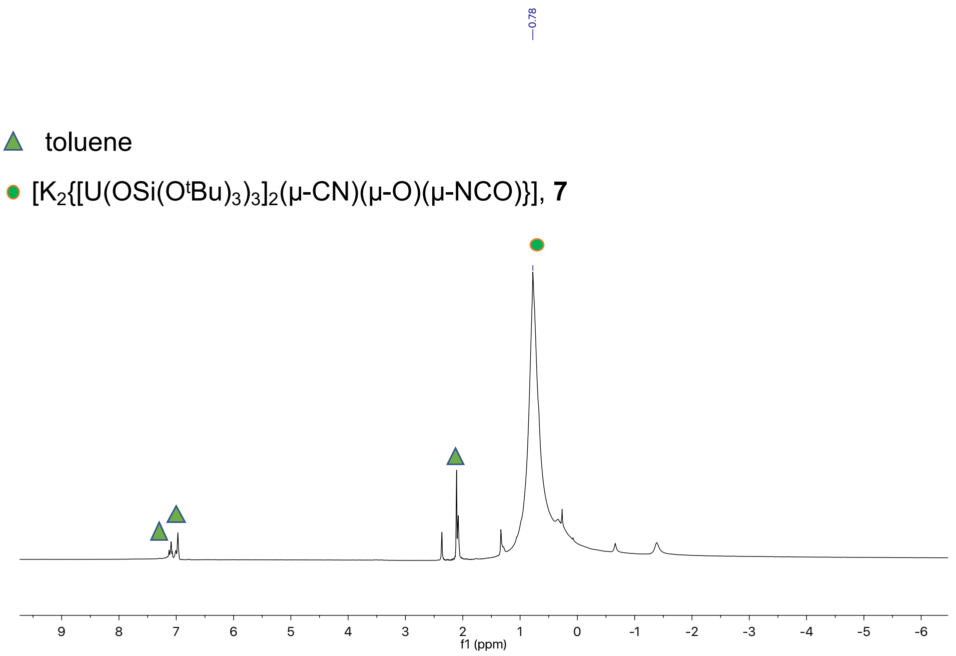

Fig. S27: ***^{1}H-NMR (400 MHz) at 298 K in d_8-tol of the reaction mixture immediately after the addition of 1 atm of CO to complex $[K_2\{[U(OSi(O^tBu)_3)_3]_2(\mu\text{-}N)_2\}]$, 4 affording complex $[K_2\{[U(OSi(O^tBu)_3)_3]_2(\mu\text{-}CN)(\mu\text{-}O)(\mu\text{-}NCO)\}]$, 7***

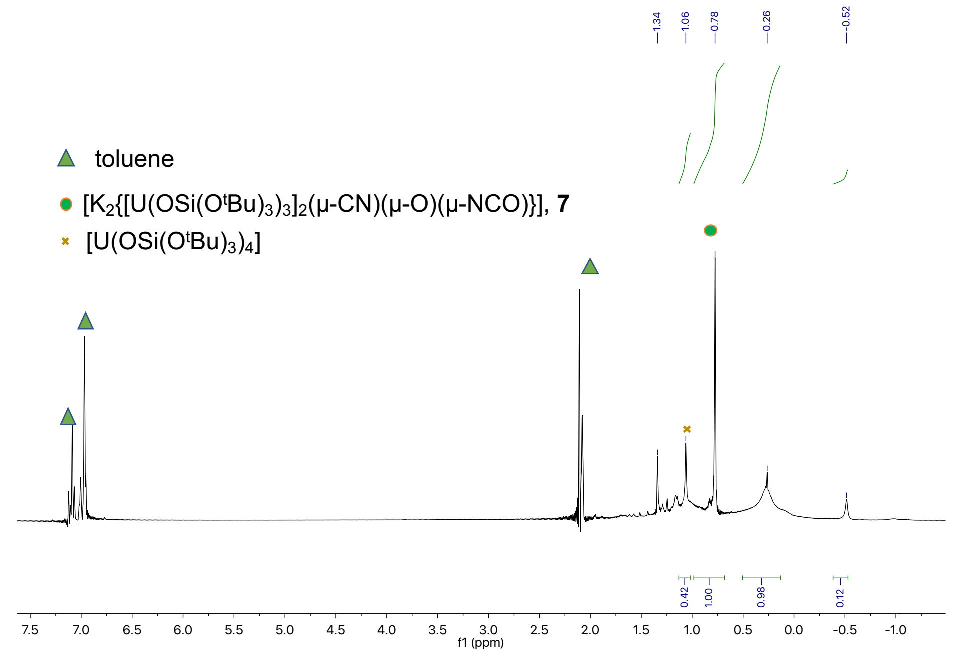

Fig. S28: ***^{1}H-NMR (400 MHz) at 298 K in d_8-tol of the reaction mixture immediately after the addition of 6 atm of CO to complex $[K_2\{[U(OSi(O^tBu)_3)_3]_2(\mu\text{-}N)_2\}]$, 4***

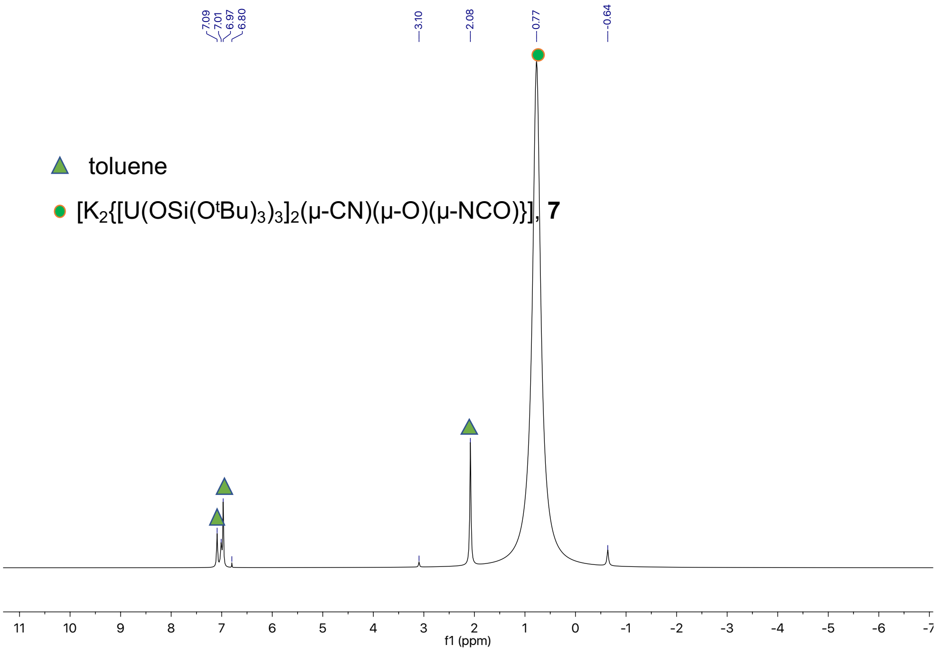

Fig. S29: ^{1}H-NMR (400 MHz) at 298 K in d_8-tol of isolated [K_2{[U(OSi(O^{t}Bu)$_3$)$_3$]$_2$(μ-CN)(μ-O)(μ-NCO)}], 7

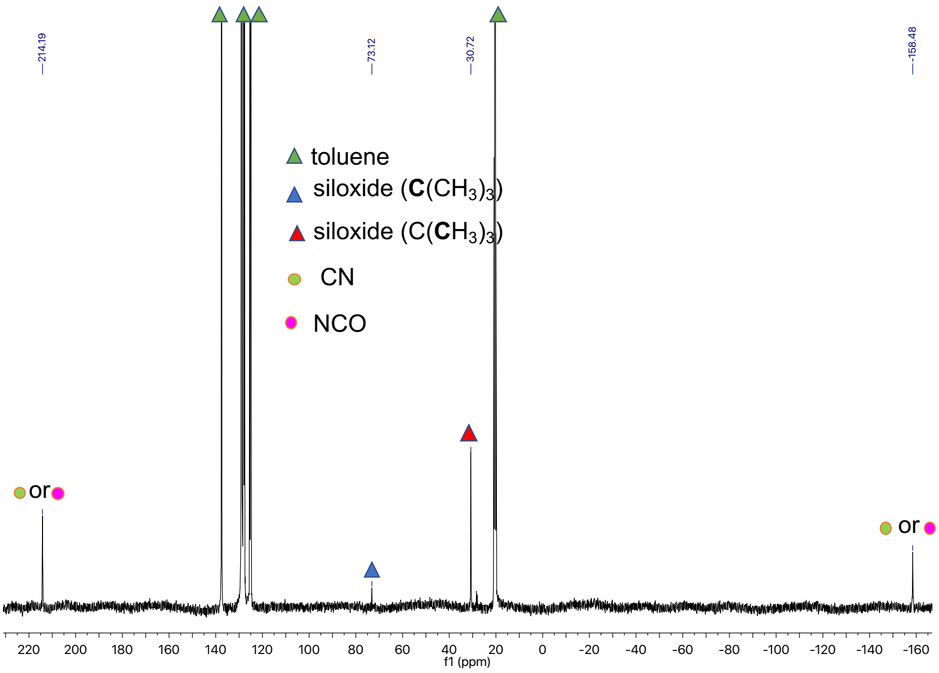

Fig. S30: ^{13}C-NMR (400 MHz) at 298 K in d_8-tol of isolated [K_2{[U(OSi(O^{t}Bu)$_3$)$_3$]$_2$(μ-^{13}CN)(μ-O)(μ-N^{13}CO)}], ^{13}C-7

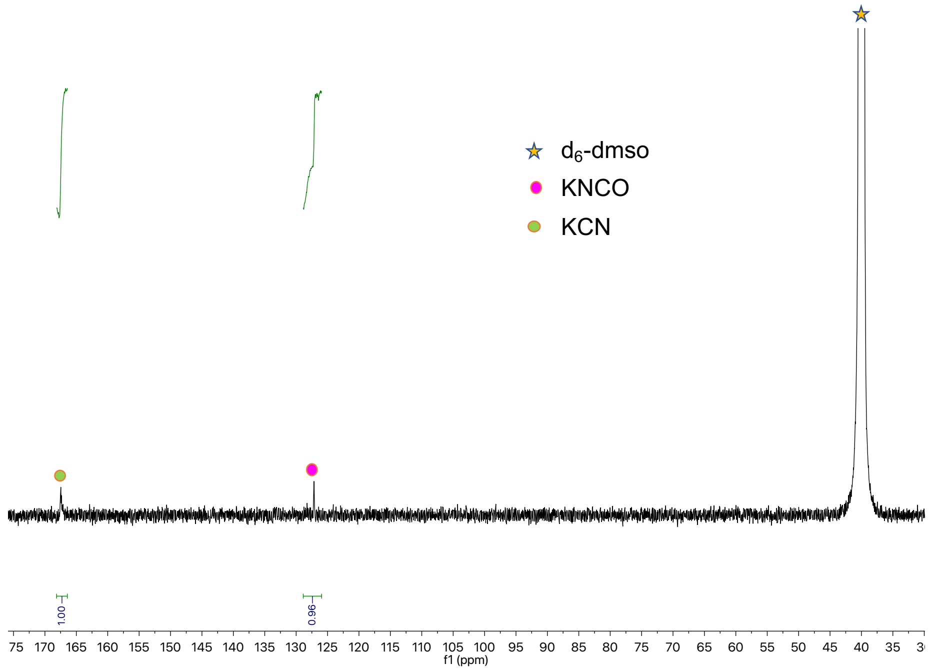

Fig. S31: Quantitative ^{13}C-NMR (600 MHz) at 298 K in d_6-dmso of isolated $[K_2\{[U(OSi(O^tBu)_3)_3]_2(\mu\text{-}^{13}CN)(\mu\text{-}O)(\mu\text{-}N^{13}CO)\}]$, ^{13}C-7

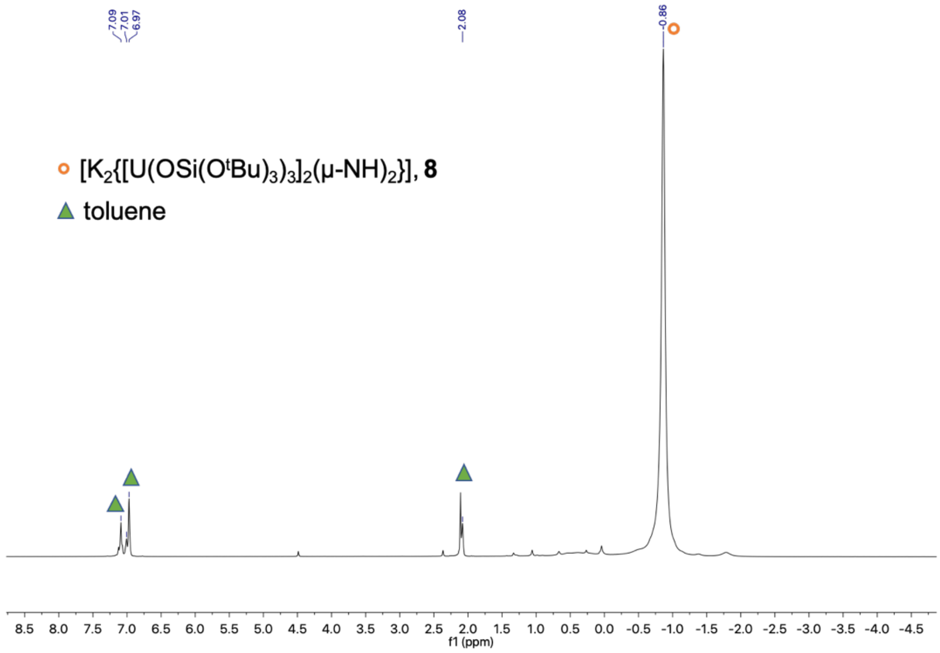

Fig. S32: ^{1}H-NMR (400 MHz) at 298 K in d_8-tol of the reaction between complex $[K_2\{[U(OSi(O^tBu)_3)_3]_2(\mu\text{-}N)_2\}]$, 4 and 1 atm of H_2 heated up at 60°C for 1h affording complex $[K_2\{[U(OSi(O^tBu)_3)_3]_2(\mu\text{-}NH)_2\}]$, 8

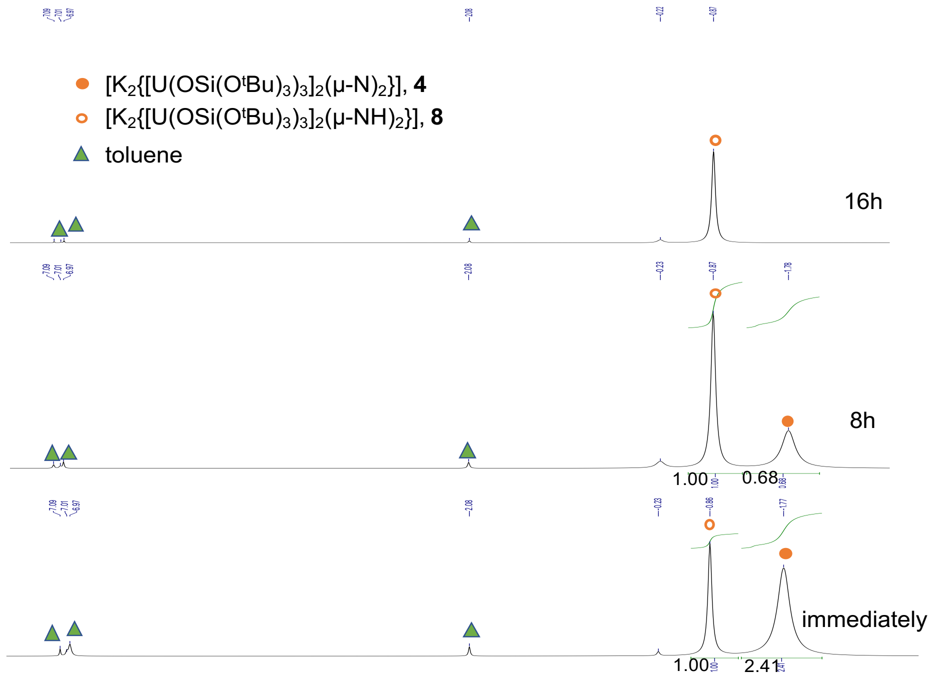

Fig. S33: Evolution of the ^{1}H-NMR (400 MHz) at 298 K in d_8-tol of the reaction mixture between [K_2{[U(OSi(O^{t}Bu)$_3$)$_3$]$_2$(μ-N)$_2$}], 4 and 1 atm of H_2 affording complex [K_2{[U(OSi(O^{t}Bu)$_3$)$_3$]$_2$(μ-NH)$_2$}], 8

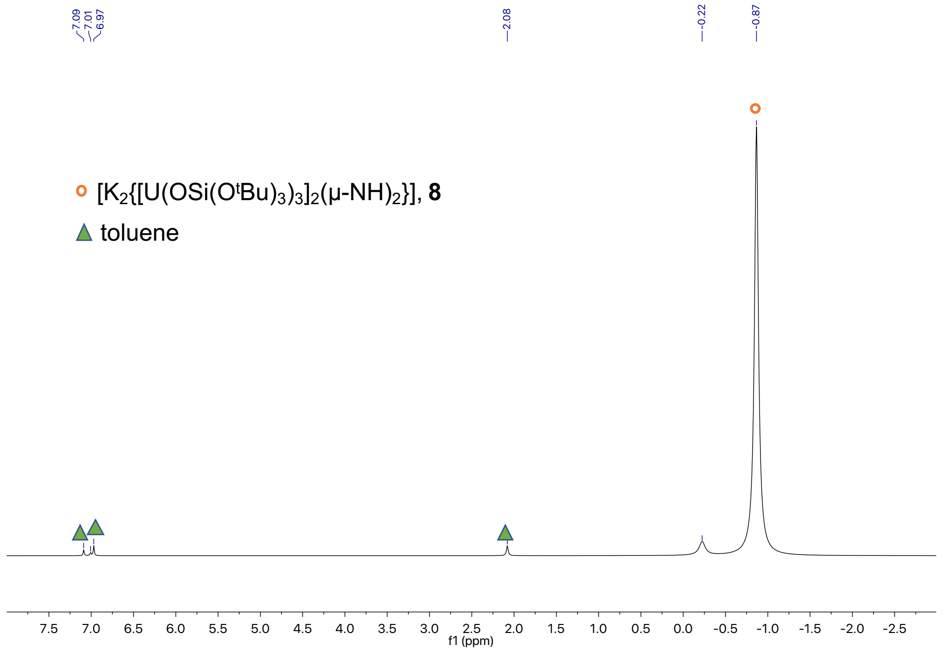

Fig. S34: ^{1}H-NMR (400 MHz) at 298 K in d_8-tol of the reaction between complex [K_2{[U(OSi(O^{t}Bu)$_3$)$_3$]$_2$(μ-N)$_2$}], 4 and 1 atm of H_2 after 16h at RT affording complex [K_2{[U(OSi(O^{t}Bu)$_3$)$_3$]$_2$(μ-NH)$_2$}], 8

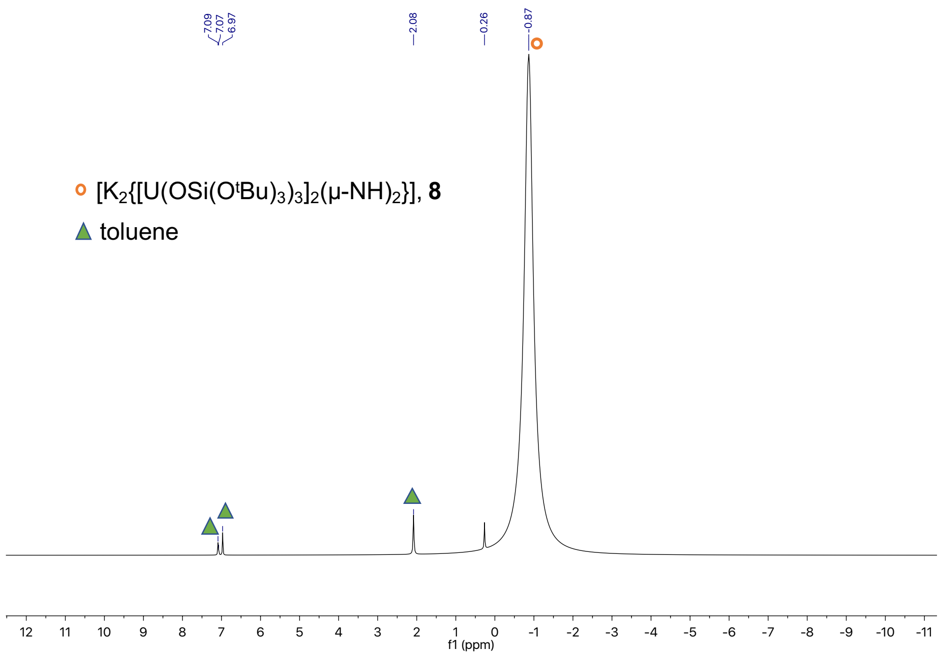

Fig. S35: ^{1}H-NMR (400 MHz) at 298 K in d_8-tol of isolated [K_2{[U(OSi(O^{t}Bu)$_3$)$_3$]$_2$(μ-NH)$_2$}], 8

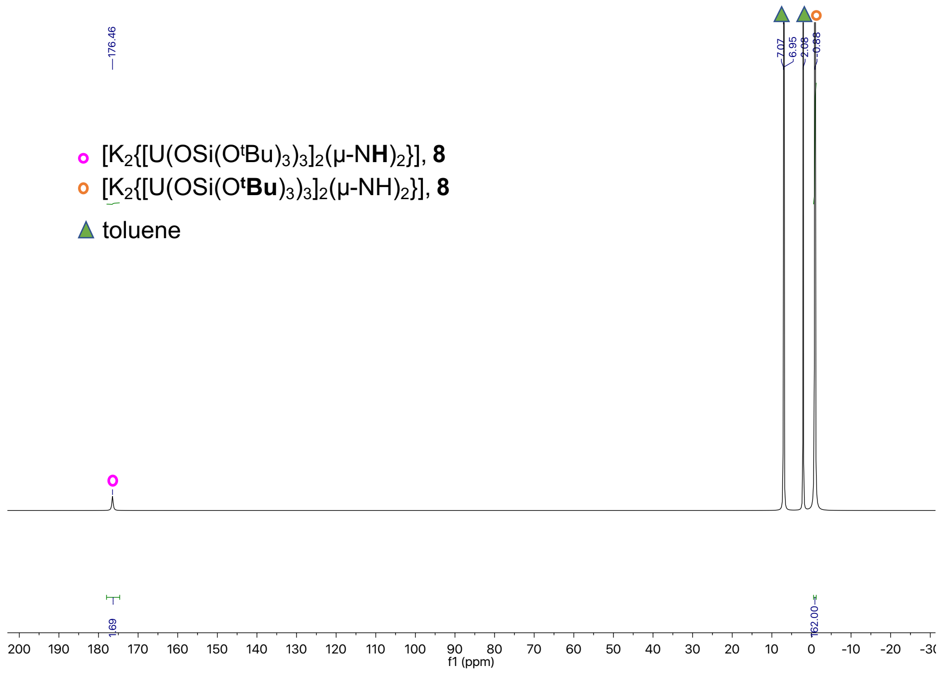

Fig. S36: ^{1}H-NMR (400 MHz) at 298 K in d_8-tol of isolated [K_2{[U(OSi(O^{t}Bu)$_3$)$_3$]$_2$(μ-NH)$_2$}], 8 on a broader spectral window

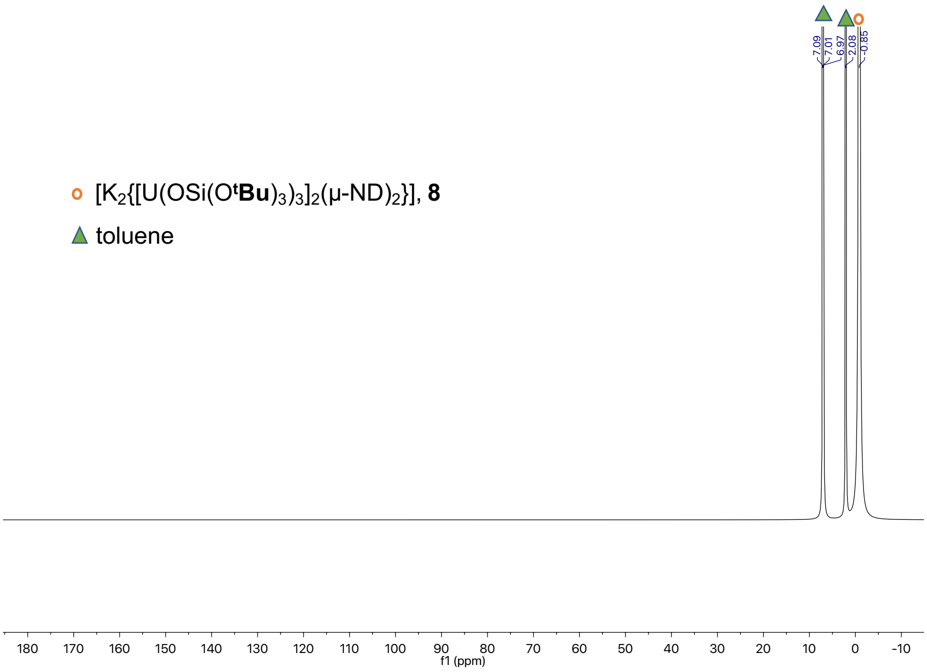

Fig. S37: ***^{1}H-NMR (400 MHz) at 298 K in d_8-tol of the reaction between complex [K_2{[U(OSi(O^{t}Bu)$_3$)$_3$]$_2$(μ-N)$_2$}], 4 and D_2 heated up at 60°C for 1h affording complex [K_2{[U(OSi(O^{t}Bu)$_3$)$_3$]$_2$(μ-ND)$_2$}], 8***

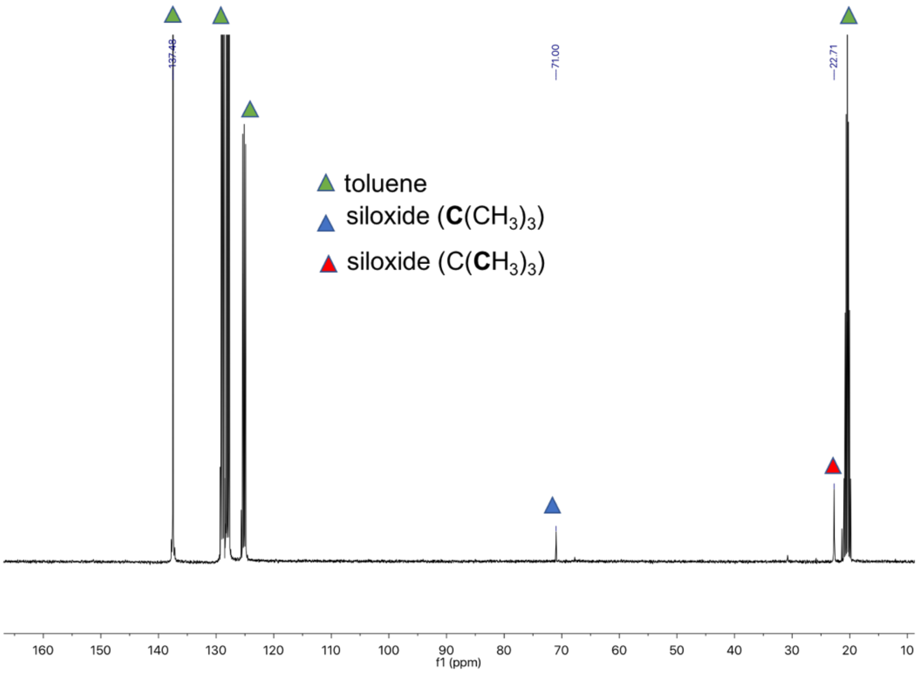

Fig. S38: ***^{13}C-NMR (400 MHz) at 298 K in d_8-tol of isolated [K_2{[U(OSi(O^{t}Bu)$_3$)$_3$]$_2$(μ-NH)$_2$}], 8***

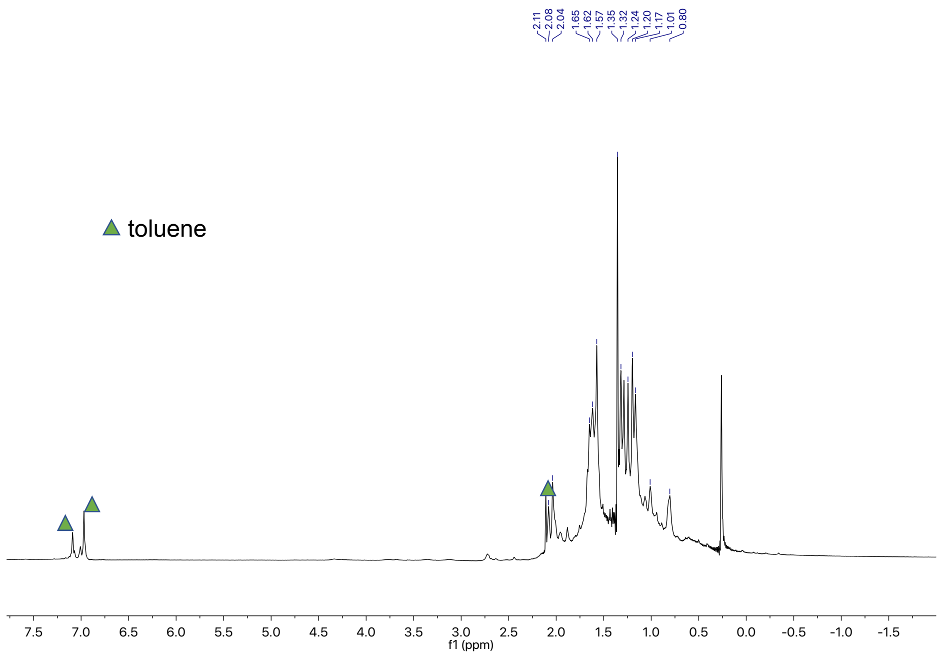

Fig. S39: ***^{1}H-NMR (400 MHz) at 298 K in d_8-tol of the reaction between complex $[K_2\{[U(OSi(O^tBu)_3)_3]_2(\mu\text{-}N)_2\}]$, 4 and 5.5 atm of H_2 after 3h at RT***

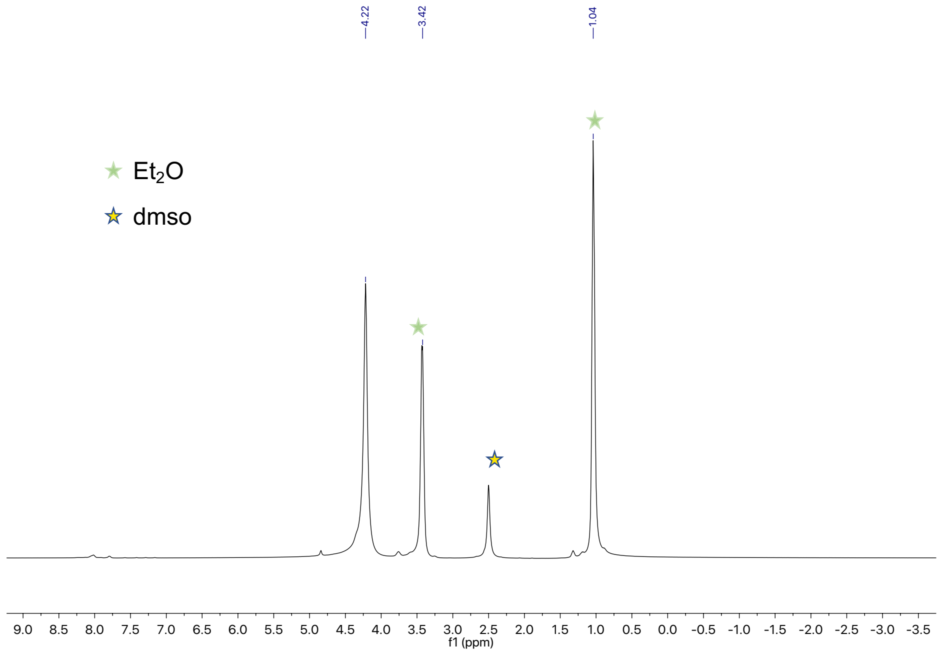

Fig. S40: ***^{1}H-NMR (400 MHz) at 298 K in d_6-dmso of the volatiles and the headspace of the reaction between complex $[K_2\{[U(OSi(O^tBu)_3)_3]_2(\mu\text{-}N)_2\}]$, 4 and 5.5 atm of H_2 after 3h at RT collected in a frozen 2M solution of HCl in Et_2O***

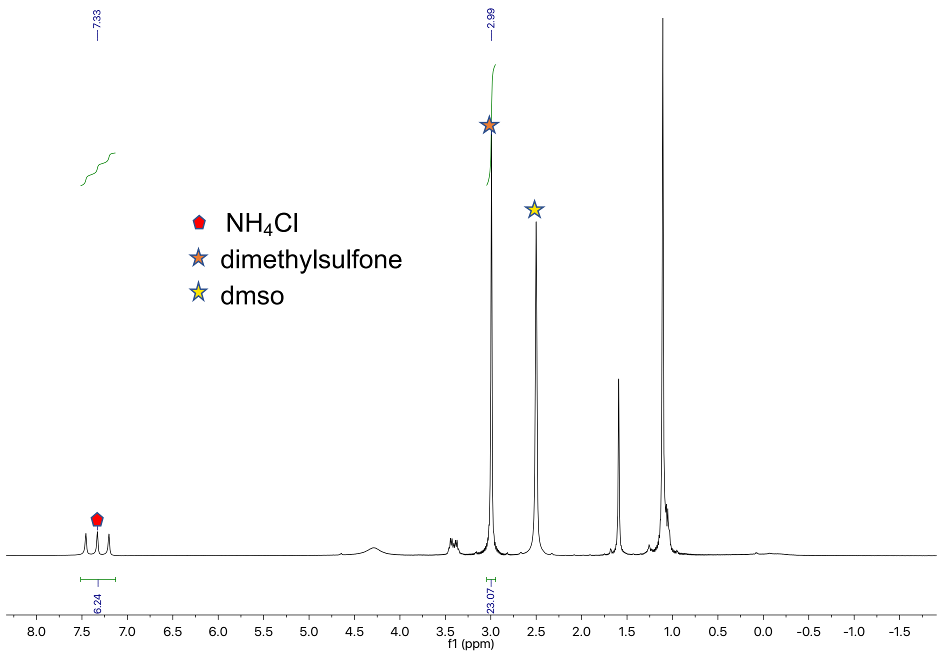

Fig. S41: ^{1}H-NMR (400 MHz) at 298 K in d_6-dmso of the reaction mixture between solid complex 4 and an excess of a 2M solution of HCl in Et_2O

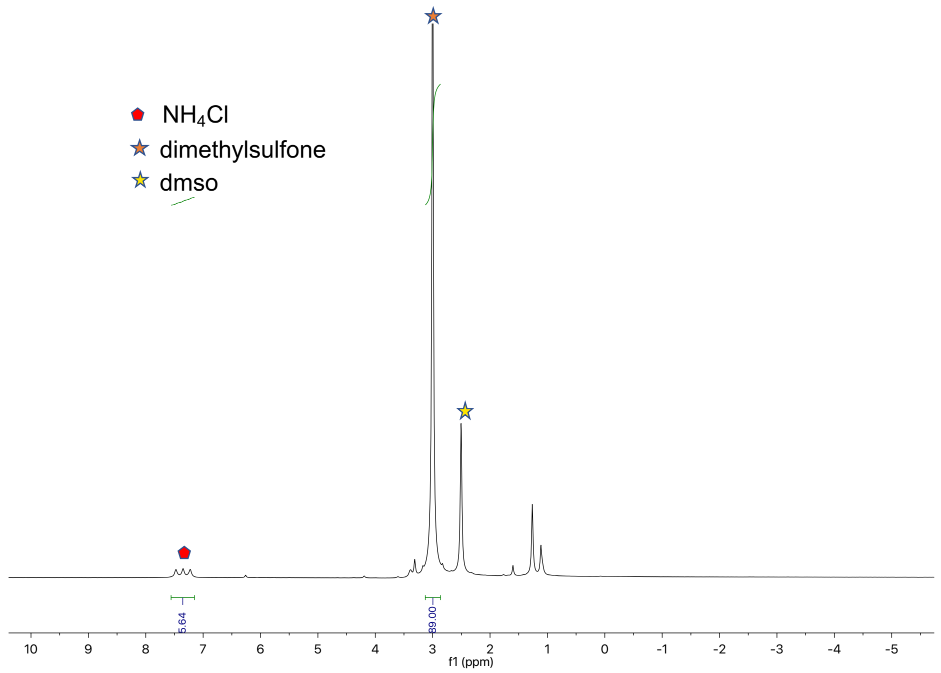

Fig. S42: ^{1}H-NMR (400 MHz) at 298 K in d_6-dmso of the reaction mixture between solid complex 8 and an excess of a 2M solution of HCl in Et_2O

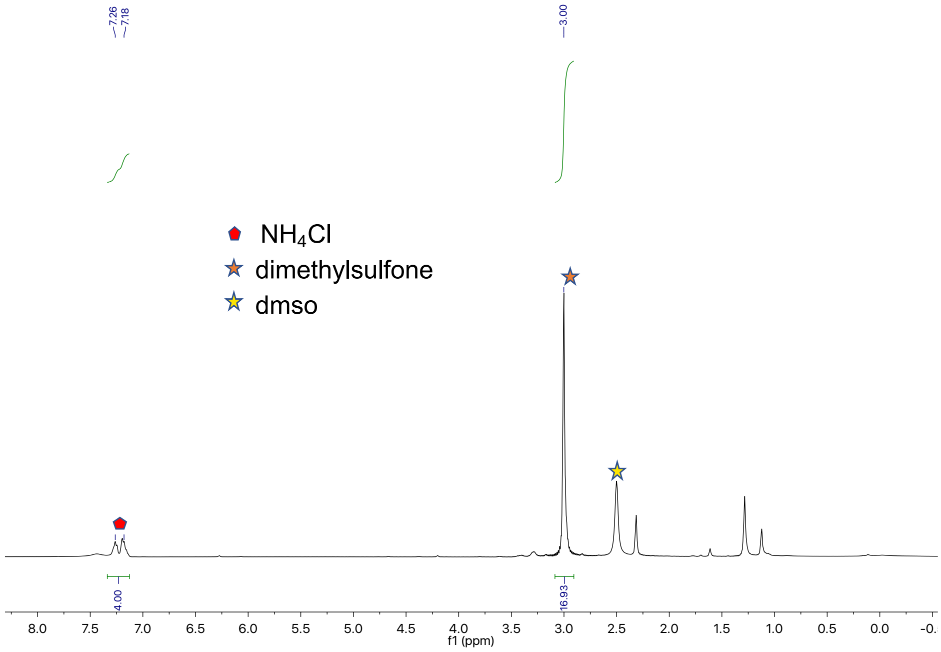

***Fig. S43:** ^{1}H-NMR (400 MHz) at 298 K in d_6-dmso of the reaction mixture between solid complex 8 and an excess of a 2M solution of HCl in Et_2O in presence of excess KC_8*

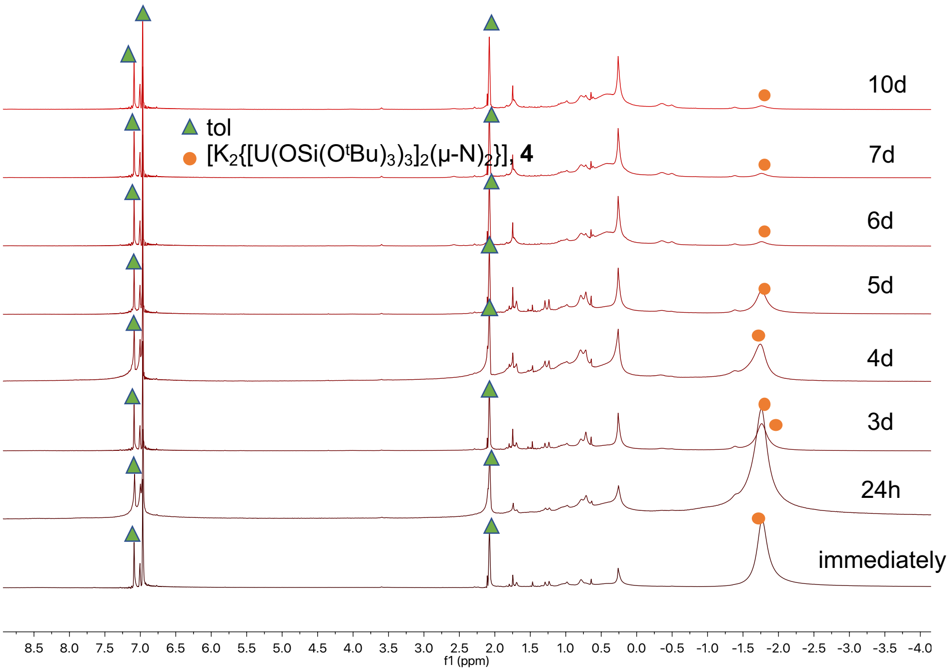

***Fig. S44:** Evolution of the ^{1}H-NMR (400 MHz) at 298 K in d_8-tol of the reaction mixture between $[K_2\{[U(OSi(O^tBu)_3)_3]_2(\mu\text{-}N)_2\}]$, 4 and 1 eq of PyHOTf*

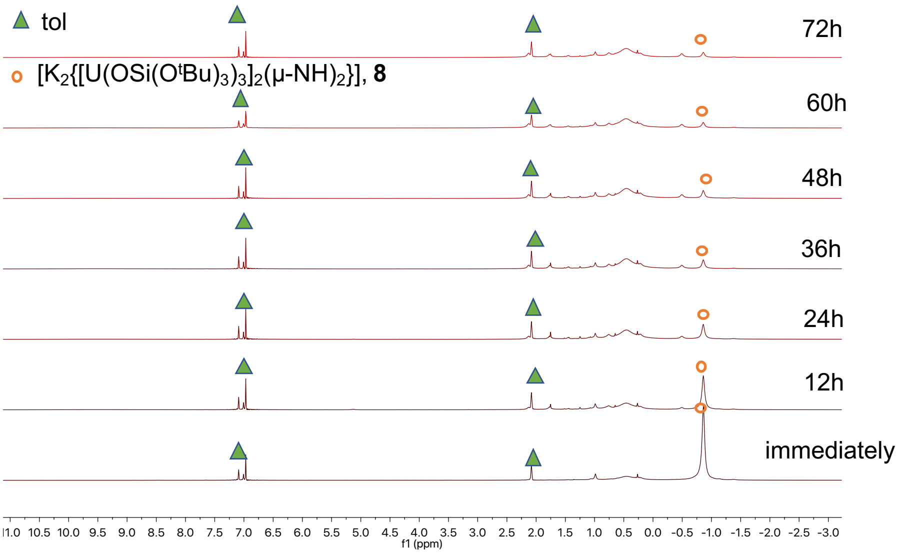

*Fig. S45: Evolution of the ^{1}H-NMR (400 MHz) at 298 K in d_8-tol of the reaction mixture between $[K_2\{[U(OSi(O^tBu)_3)_3]_2(\mu\text{-}NH)_2\}]$, **8** and 1 eq of PyHOTf*

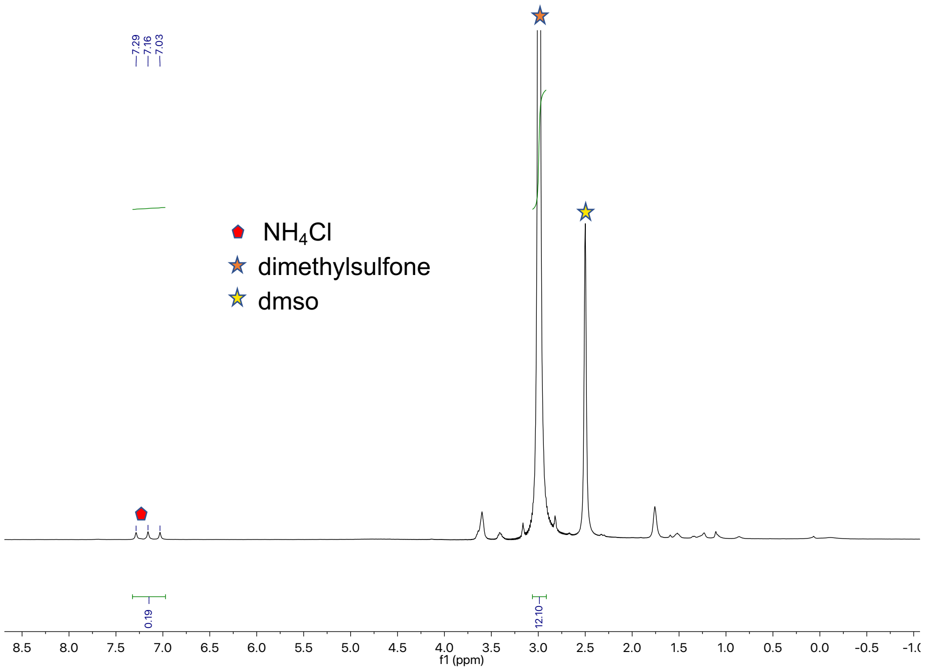

*Fig. S46: ^{1}H-NMR (400 MHz) at 298 K in d_6-dmso of the volatiles and the headspace of the reaction mixture between between $[K_2\{[U(OSi(O^tBu)_3)_3]_2(\mu\text{-}N)_2\}]$, **4** and 30 eq of H_2O in thf collected in a frozen 2M solution of HCl in Et_2O*

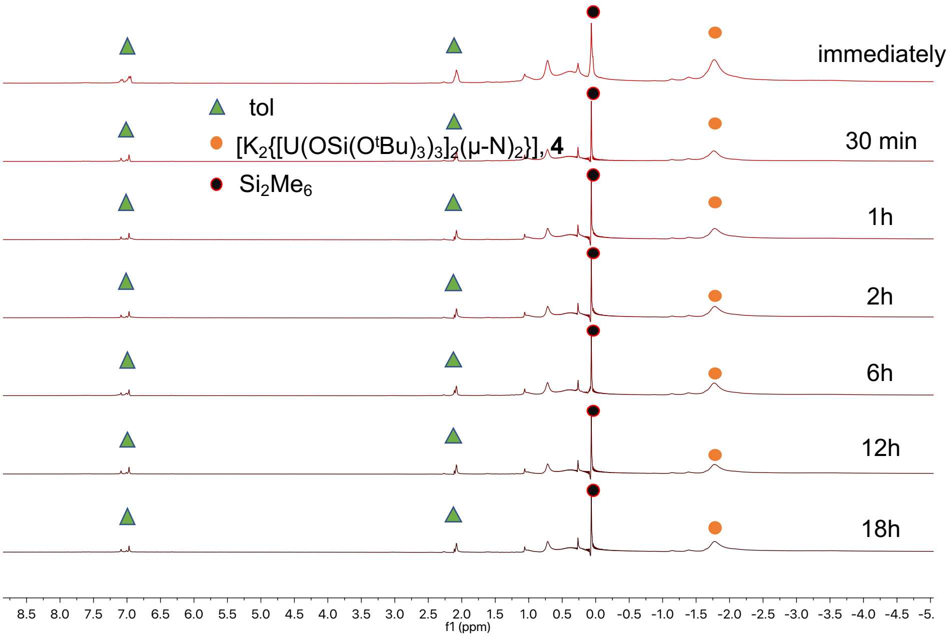

Fig. S47: Evolution of the ^{1}H-NMR (400 MHz) at 298 K in d_8-tol of the reaction mixture between [K_2{[U(OSi($O^tBu)_3)_3]_2$(μ-N)$_2$}], 4 and 1 eq of Si_2Me_6. The formation of an unidentified yellow precipitate is also observed.

B) IR spectra

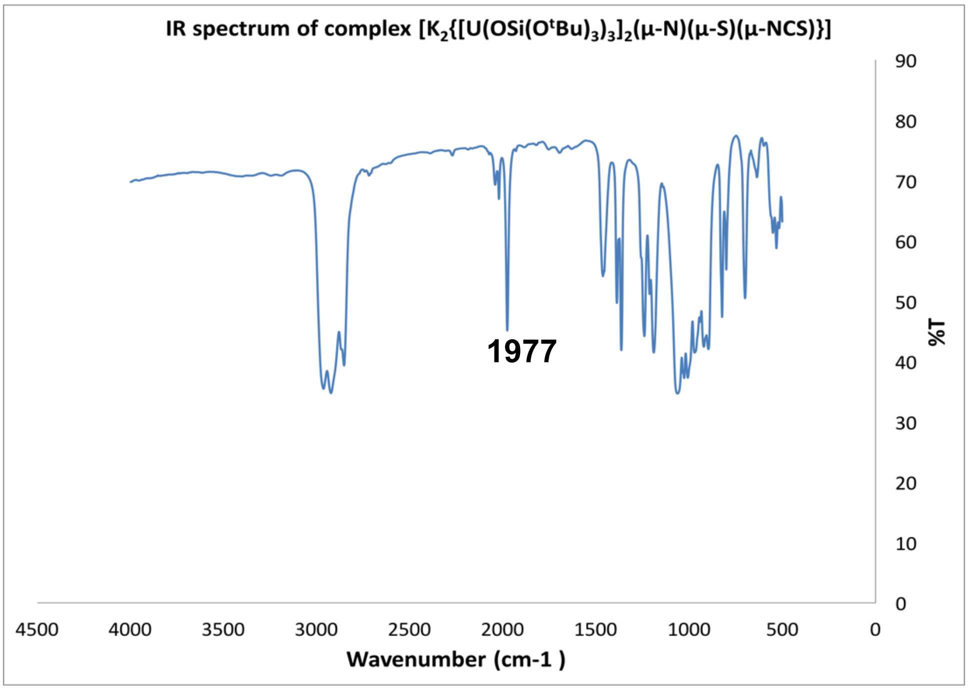

Fig. S48: IR spectrum of isolated complex [K_2{[U(OSi($O^tBu)_3)_3]_2$(μ-S)(μ-N)(μ-NCS)}], 5 in a Nujol suspension measured on KBr windows.

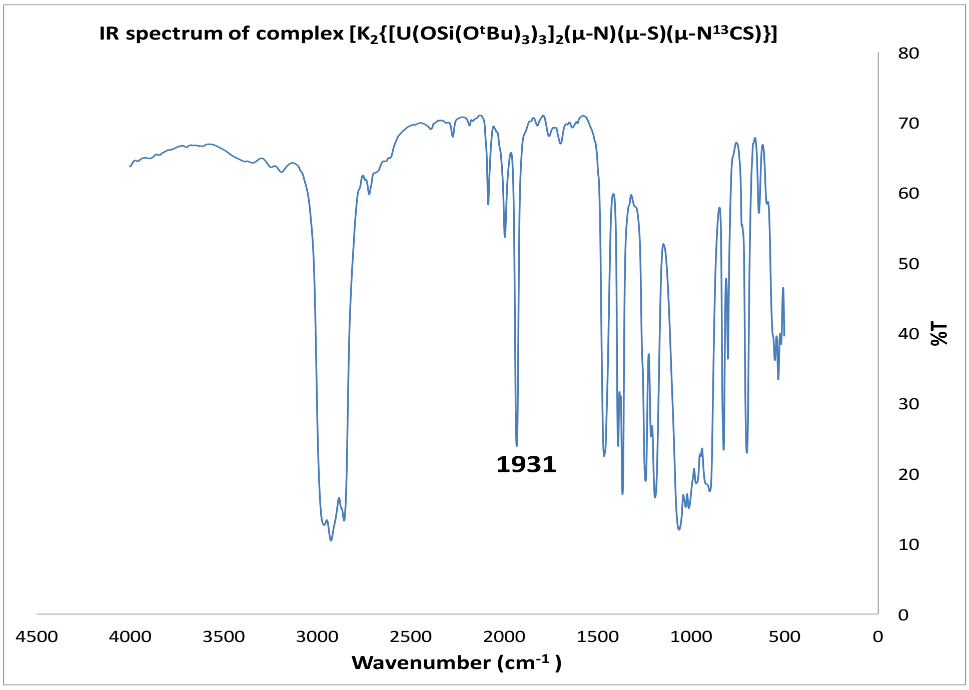

Fig. S49: IR spectrum of isolated complex [$K_2\{[U(OSi(O^tBu)_3)_3]_2$(μ-O)(μ-N)(μ-$N^{13}CS$)}], 5 in a Nujol suspension measured on KBr windows.

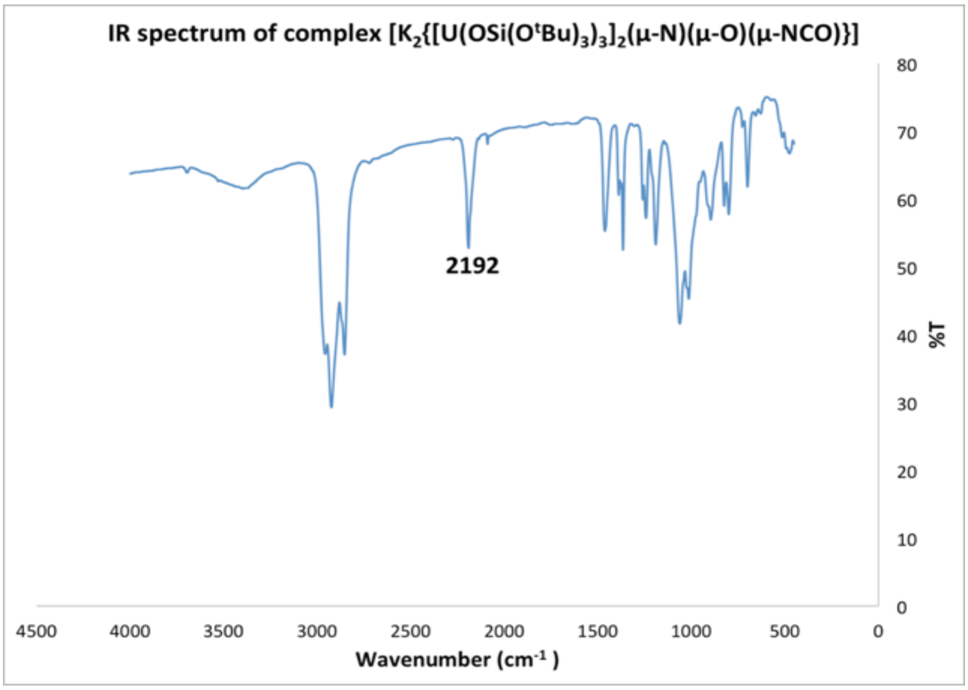

Fig. S50: IR spectrum of isolated complex [$K_2\{[U(OSi(O^tBu)_3)_3]_2$(μ-O)(μ-N)(μ-NCO)}], 6 in a Nujol suspension measured on KBr windows.

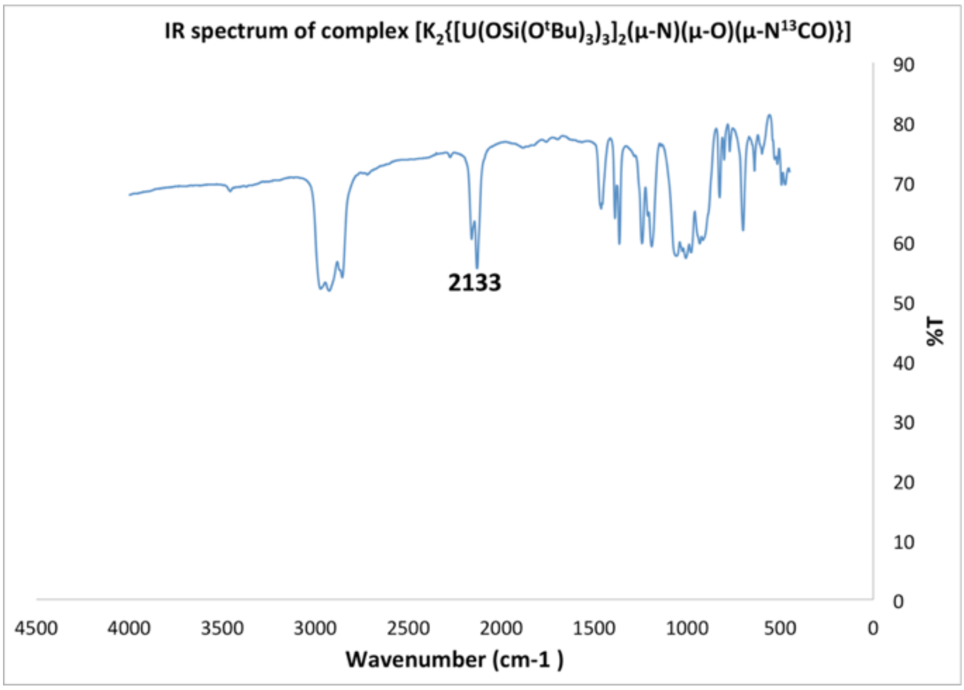

Fig. S51: IR spectrum of isolated complex $[K_2\{[U(OSi(O^tBu)_3)_3]_2(\mu\text{-}O)(\mu\text{-}N)(\mu\text{-}N^{13}CO)\}]$, ^{13}C-6 in a Nujol suspension measured on KBr windows.

C) X-Ray crystallographic data

	**2**	**3**	**5**
Formula	$C_{72}H_{162}KNO_{24}Si_6U_2$	$C_{72}H_{162}K_2N_4O_{24}Si_6U_2$	$C_{73}H_{162}K_2N_2O_{24}S_2Si_6U_2$
Crystal size (mm^3)	0.642 x 0.503 x 0.419	0.48×0.31×0.26	0.12×0.06×0.04
crystal system	Monoclinic	Monoclinic	monoclinic
space group	$P2_1/c$	$P2_1/n$	$P2_1$
volume (Å^3)	10803(2)	5538.4(16)	5619.3(4)
a (Å)	14.210(3)	14.382(2)	13.9065(4)
b (Å)	28.1537(19)	17.661(3)	18.2338(7)
c (Å)	27.910(3)	21.819(4)	22.1810(9)
α (deg)	90	90	90
β (deg)	104.632(8)	92.012(9)	92.450(4)
γ (deg)	90	90	90
Z	4	2	2
formula weight (g/mol)	2109.72	2190.85	2238.96
density (g cm^{-3})	1.297	1.314	1.323
absorption coefficient (mm^{-1})	3.156	3.118	10.139
F(000)	4320	2240	2288
temp (K)	100(2)	100(2)	139.99(10)
total no. reflections	115428	97679	38004
unique reflections [R(int)]	24309 [0.1089]	19106 [0.0447]	13621 [0.0881]

Final R indices [I > 2σ(I)]	R1 = 0.0686, wR2 = 0.1330	R1 = 0.0592, wR2 = 0.1241	R1 = 0.0772, wR2 = 0.1898
Largest diff. peak and hole (e.A^{-3})	2.689 and -2.107	4.561 and -2.717	3.583 and -4.163
GOOF	1.111	1.135	1.074

	**6**	**7**	**8**
Formula	$C_{73}H_{162}K_2N_2O_{26}Si_6U_2$	$C_{81}H_{170}K_2N_2O_{26}Si_6U_2$	$C_{79}H_{172}K_2N_2O_{24}Si_6U_2$
Crystal size (mm^3)	0.10×0.06×0.04	0.19×0.09×0.08	0.76×0.42×0.35
cryst syst	Monoclinic	Monoclinic	Monoclinic
space group	$P2_1$	$P2_1$	$P2_1/n$
volume (Å^3)	5510.8(3)	5673.2(9)	5536.5(3)
a (Å)	13.9377(5)	13.9927(15)	14.4207(4)
b (Å)	17.9175(5)	18.2874(12)	17.5321(5)
c (Å)	22.0849(6)	22.208(2)	21.9098(6)
α (deg)	90	90	90
β (deg)	92.306(3)	93.321(7)	91.840(3)
γ (deg)	90	90	90
Z	2	2	2
formula weight (g/mol)	2206.84	2310.98	2256.98
density (g cm^{-3})	1.330	1.353	1.354
absorption coefficient (mm^{-1})	10.000	3.049	3.121
F(000)	2256	2368	2316
temp (K)	100.00(10)	120(2)	140.01(10)
total no. reflections	39631	67143	73741
unique reflections [R(int)]	17996 [0.0796]	20671 [0.0523]	19069 [0.0618]
Final R indices [I > 2σ(I)]	R1 = 0.0640, wR2 = 0.1592	R1 = 0.0511, wR2 = 0.1029	R1 = 0.0390, wR2 = 0.0824
Largest diff. peak and hole (e.A^{-3})	2.543 and -3.689	1.503 and -0.802	2.543 and -1.684
GOOF	1.017	1.067	1.060

D) Magnetic data

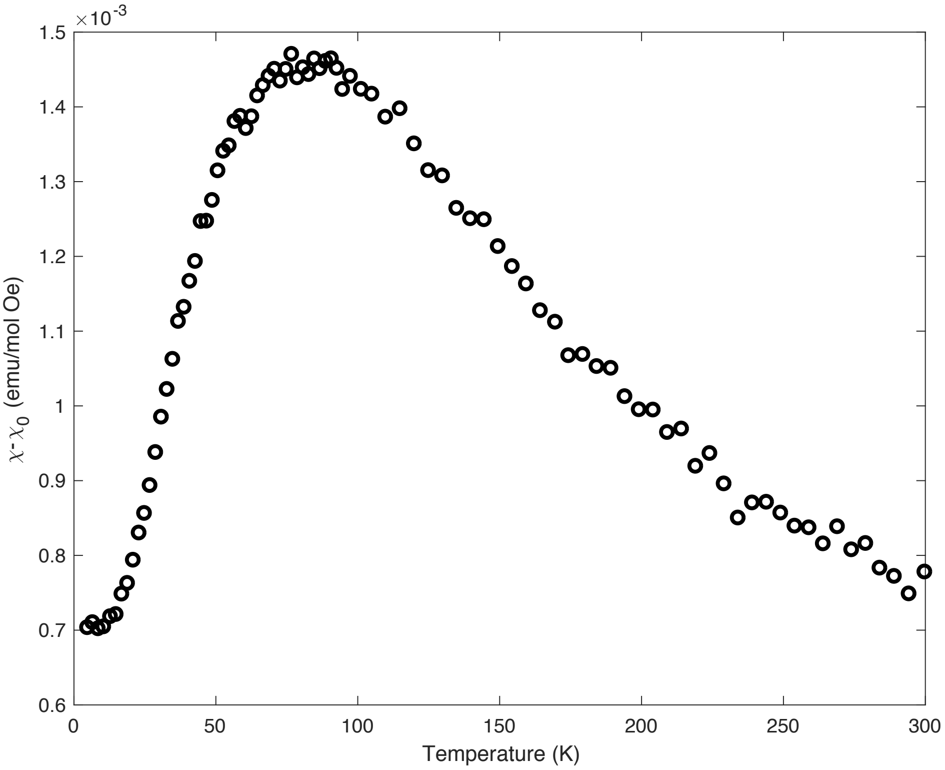

Fig. S52: *χ vs T plot for complex $[K_2\{[U(OSi(O^tBu)_3)_3]_2(\mu\text{-}N)_2\}]$, 4*

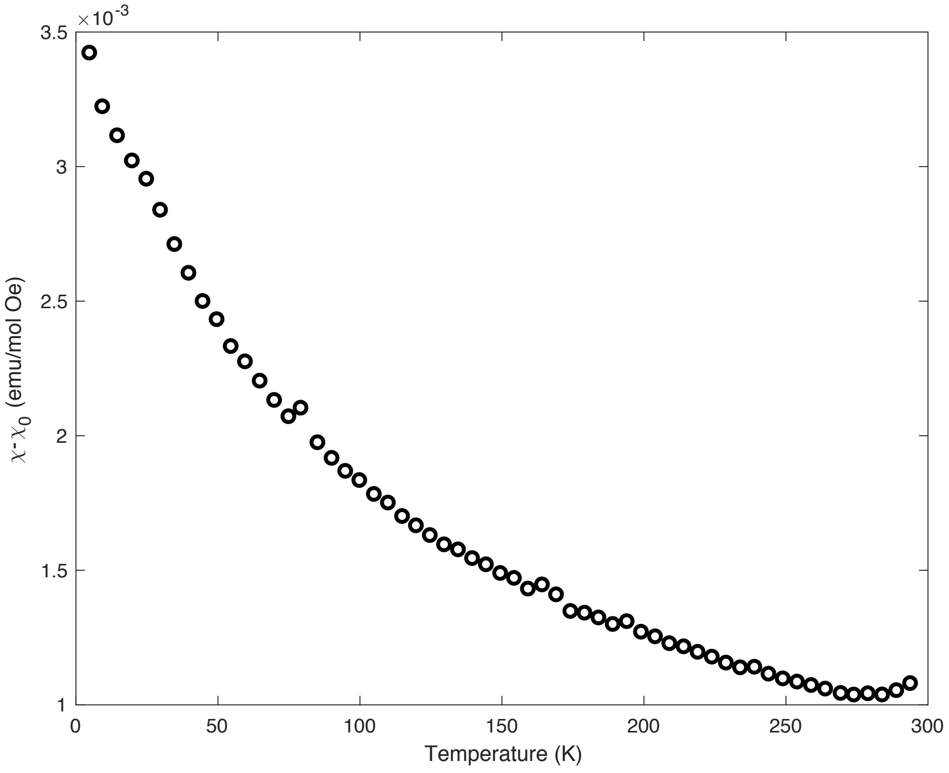

Fig. S53: χ vs T plot for complex [K_2{[U(OSi(O^tBu)$_3$)$_3$]$_2$(μ-S)(μ-N)(μ-NCS)}], 5

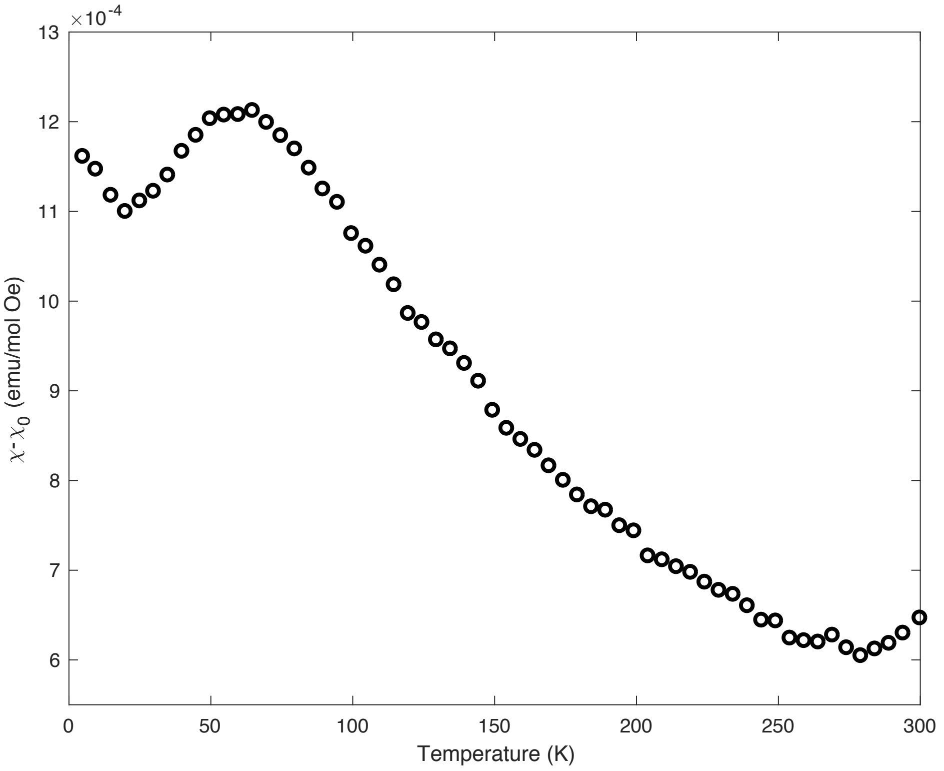

Fig. S54: χ vs T plot for complex [K_2{[U(OSi(O^tBu)$_3$)$_3$]$_2$(μ-O)(μ-N)(μ-NCO)}], 6

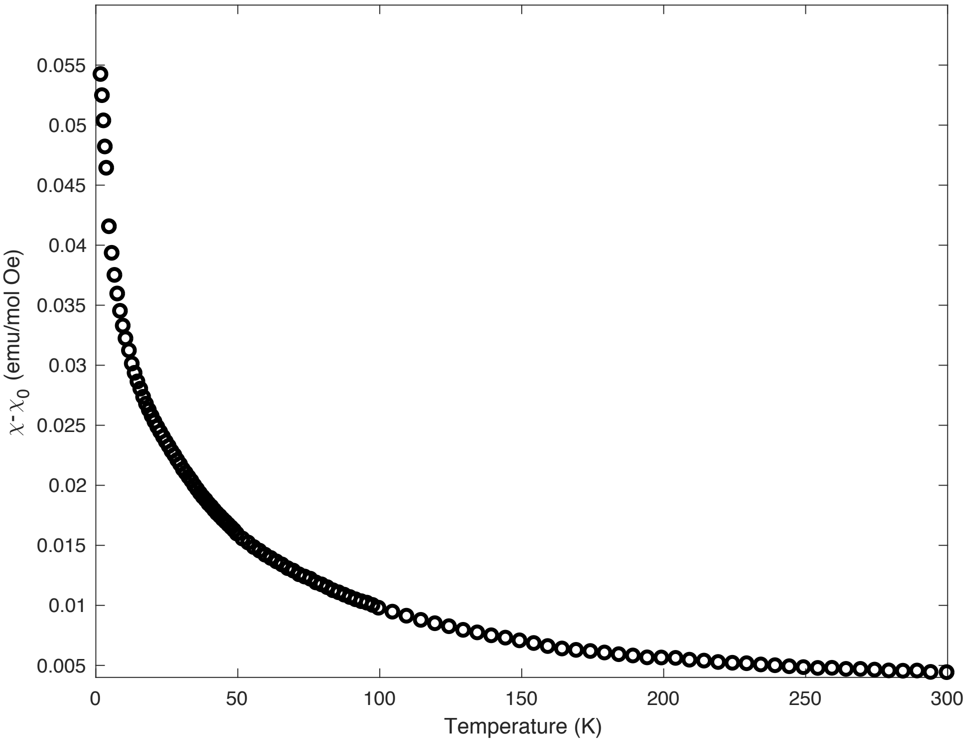

Fig. S55: χ vs T plot for complex [K{[U(OSi(O^{t}Bu)$_3$)$_3$]$_2$(μ-N)}], 2

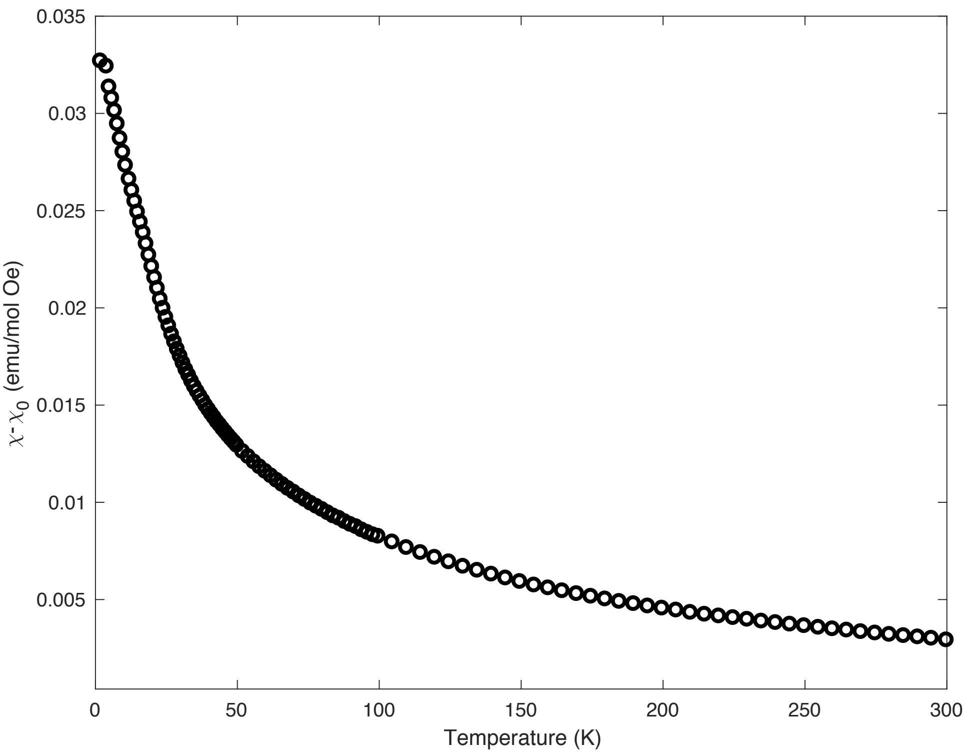

Fig. S56: χ ***vs T plot for complex*** $[K_2\{[U(OSi(O^tBu)_3)_3]_2(\mu\text{-}N)(\mu\text{-}N_3)\}]$***, 3***

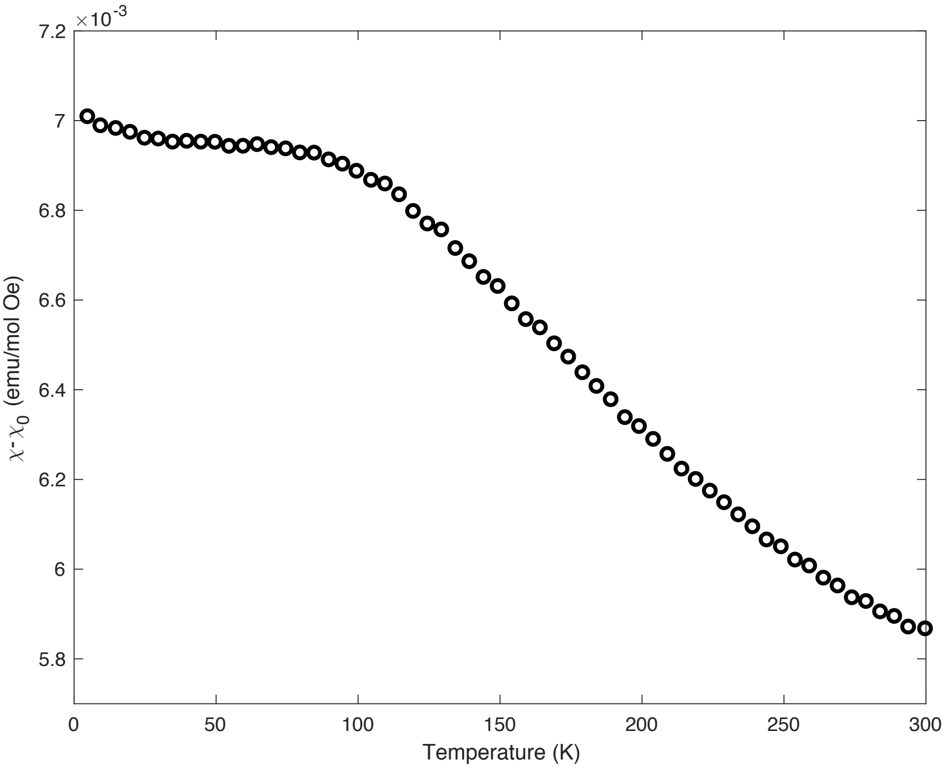

Fig. S57: χ ***vs T plot for complex*** $[K_2\{[U(OSi(O^tBu)_3)_3]_2(\mu\text{-}CN)(\mu\text{-}O)(\mu\text{-}NCO)\}]$***, 7***

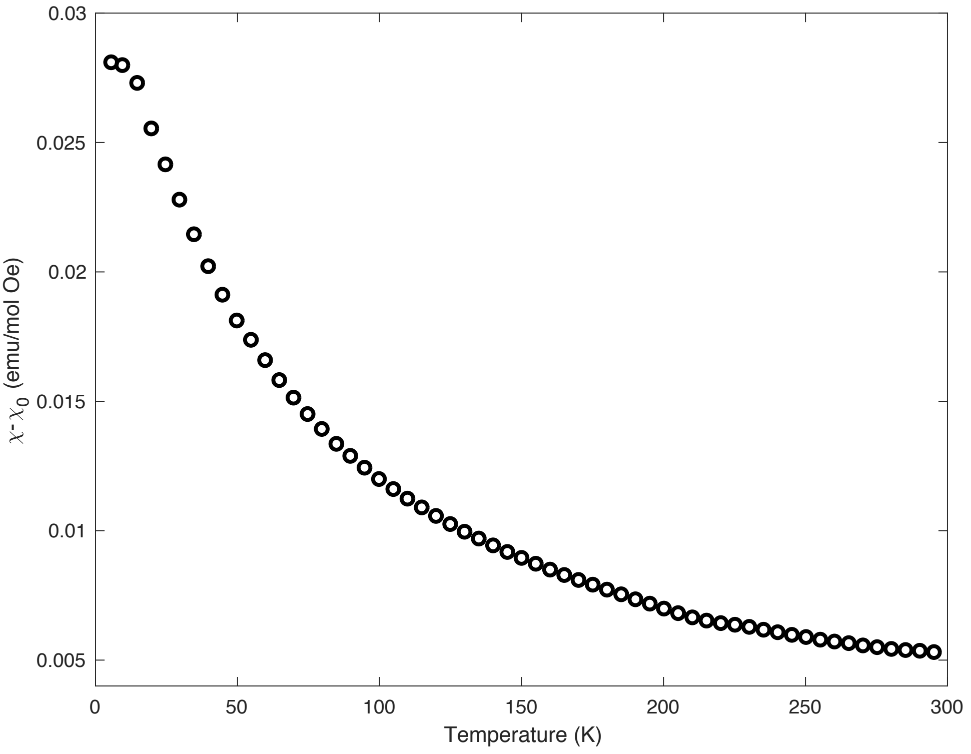

***Fig. S58:* χ *vs T plot for complex* [K_2{[U(OSi(O^tBu)$_3$)$_3$]$_2$(μ-NH)$_2$}], 8**

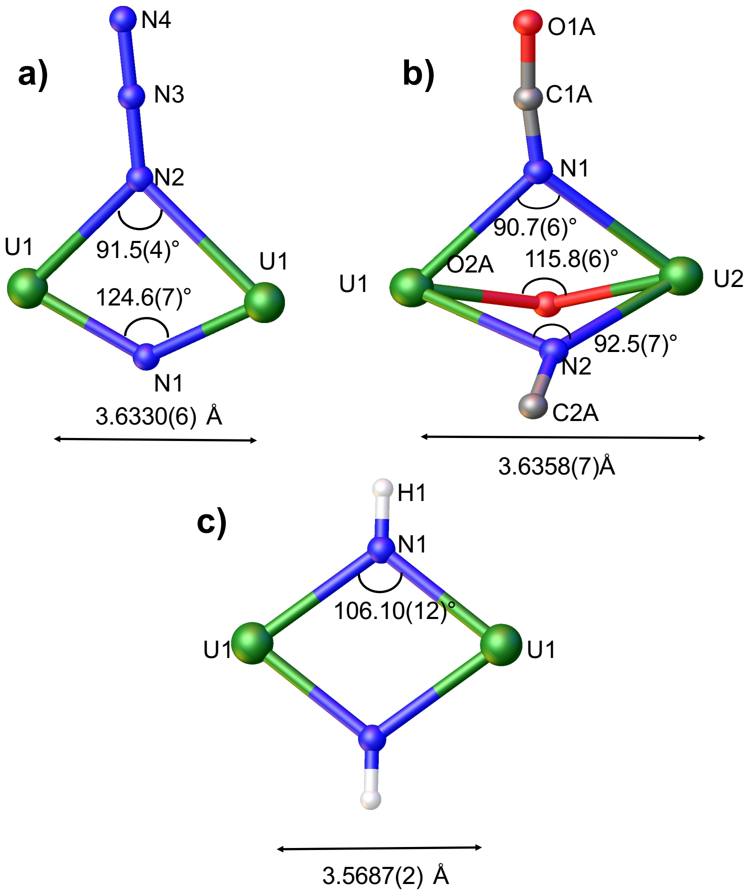

Fig. S59: Ortep diagram of the core showing the metrical parameters for the bridging atoms in complexes a) [K_2{[U(OSi(O^{t}Bu)$_3$)$_3$]$_2$(μ-N)(μ-N_3)}], 3; b) [K_2{[U(OSi(O^{t}Bu)$_3$)$_3$]$_2$(μ-CN)(μ-O)(μ-NCO)}], 7; c) [K_2{[U(OSi(O^{t}Bu)$_3$)$_3$]$_2$(μ-NH)$_2$}], 8

E) UV-Vis data

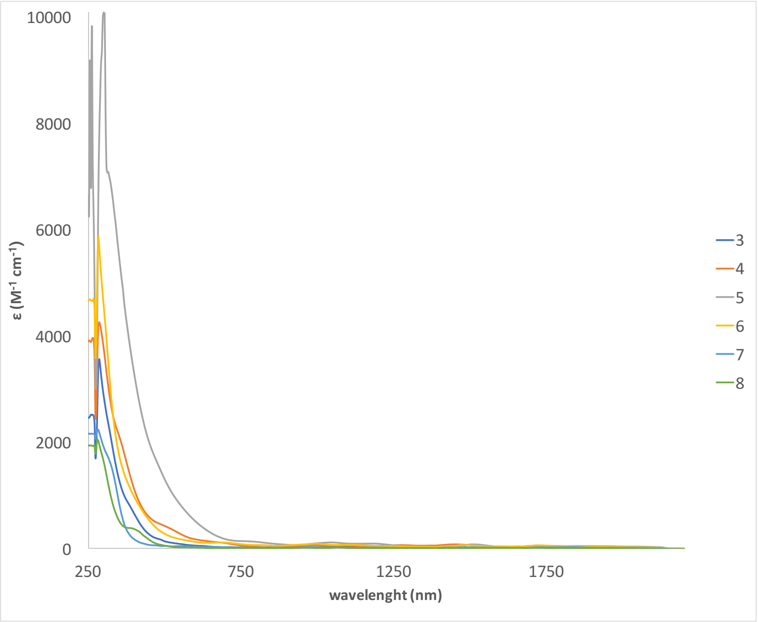

Fig. S60: UV-Vis absorption spectra in toluene solution of complexes 3-8.

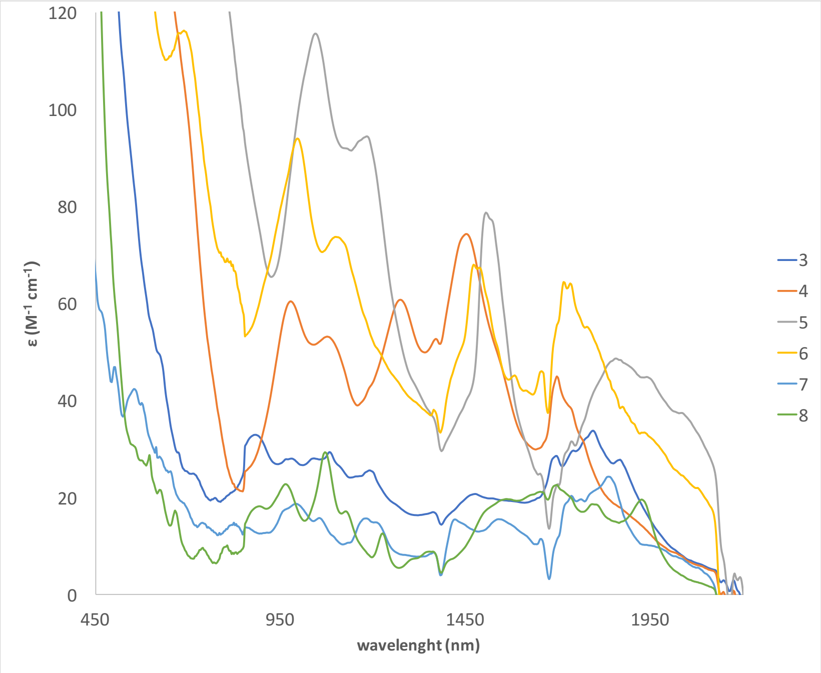

Fig. S61: NIR absorption spectra in toluene solution of complexes 3-8.
